# Room‐Temperature Multiple Phosphorescence from Functionalized Corannulenes: Temperature Sensing and Afterglow Organic Light‐Emitting Diode**

**DOI:** 10.1002/anie.202309718

**Published:** 2023-09-15

**Authors:** Changfeng Si, Tao Wang, Abhishek Kumar Gupta, David B. Cordes, Alexandra M. Z. Slawin, Jay S. Siegel, Eli Zysman‐Colman

**Affiliations:** ^1^ Organic Semiconductor Centre EaStCHEM School of Chemistry University of St Andrews St. Andrews KY16 9ST UK; ^2^ School of Pharmaceutical Science and Technology Tianjin University Tianjin 300072 P. R. China; ^3^ Institute of Organic Chemistry Albert Ludwig University of Freiburg Albertstr. 21 79104 Freiburg

**Keywords:** Afterglow Organic Light-Emitting Diode, Corannulene, Multiple Phosphorescence, Room-Temperature Phosphorescence, Temperature Sensing

## Abstract

Corannulene‐derived materials have been extensively explored in energy storage and solar cells, however, are rarely documented as emitters in light‐emitting sensors and organic light‐emitting diodes (OLEDs), due to low exciton utilization. Here, we report a family of multi‐donor and acceptor (multi‐D‐A) motifs, TCzPhCor, TDMACPhCor, and TPXZPhCor, using corannulene as the acceptor and carbazole (Cz), 9,10‐dihydro‐9,10‐dimethylacridine (DMAC), and phenoxazine (PXZ) as the donor, respectively. By decorating corannulene with different donors, multiple phosphorescence is realized. Theoretical and photophysical investigations reveal that TCzPhCor shows room‐temperature phosphorescence (RTP) from the lowest‐lying T_1_; however, for TDMACPhCor, dual RTP originating from a higher‐lying T_1_ (T_1_^H^) and a lower‐lying T_1_ (T_1_^L^) can be observed, while for TPXZPhCor, T_1_^H^‐dominated RTP occurs resulting from a stabilized high‐energy T_1_ geometry. Benefiting from the high‐temperature sensitivity of TPXZPhCor, high color‐resolution temperature sensing is achieved. Besides, due to degenerate S_1_ and T_1_^H^ states of TPXZPhCor, the first corannulene‐based solution‐processed afterglow OLEDs is investigated. The afterglow OLED with TPXZPhCor shows a maximum external quantum efficiency (EQE_max_) and a luminance (L_max_) of 3.3 % and 5167 cd m^−2^, respectively, which is one of the most efficient afterglow RTP OLEDs reported to date.

## Graphical abstract

Corannulene‐based luminophores that exhibit room‐temperature multiple phosphorescence are reported for the first time. We demonstrate utility of these room‐temperature phosphorescence materials in an afterglow organic light‐emitting diode and for optical temperature sensing stemming from a change in the ratio of multiple phosphorescence yields as a function of temperature.

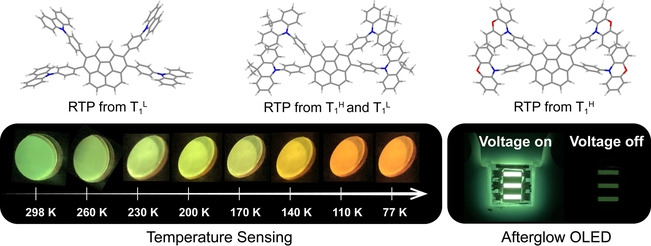

## Introduction

**Figure 1:**
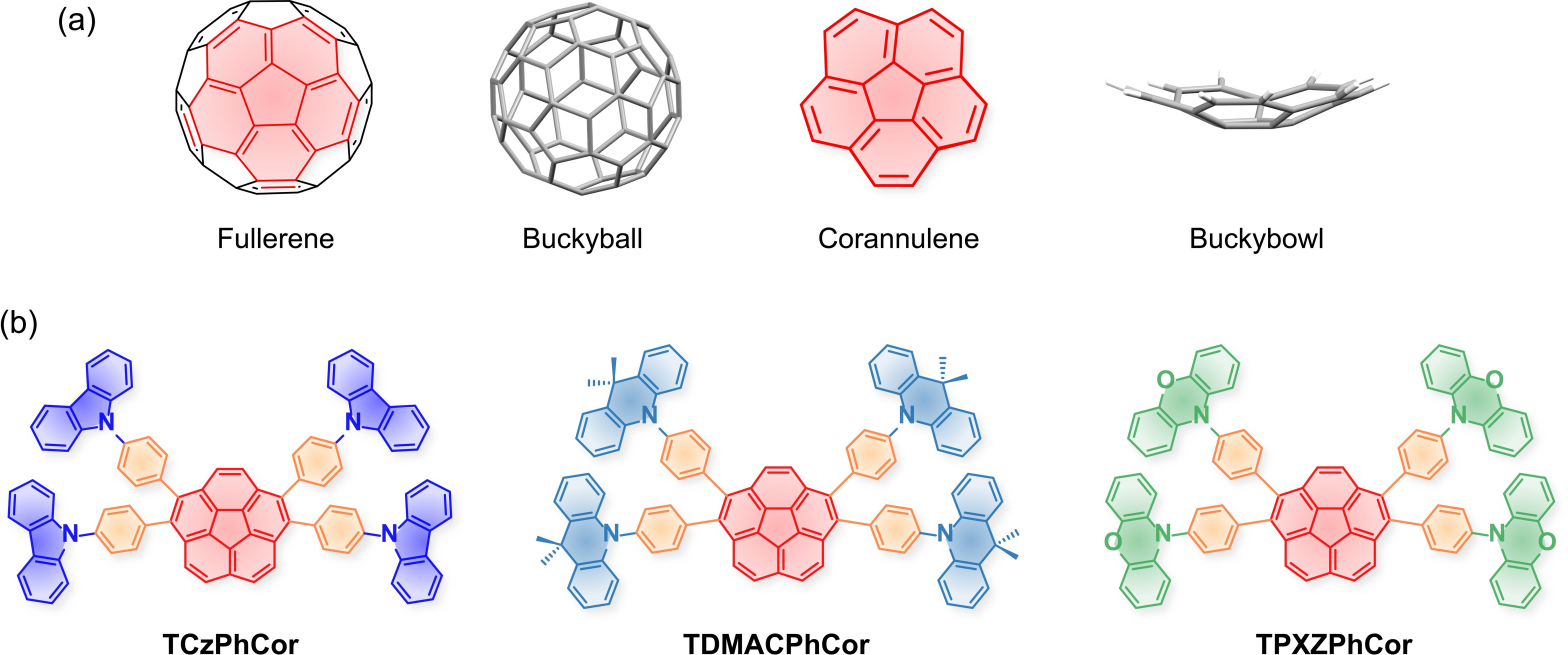
(a) Chemical structures of (a) fullerene and corannulene and (b) multi‐D‐A corannulenes reported in this work.

Corannulene, C_20_H_10_, also known as a buckybowl, is a curved polyaromatic hydrocarbon (PAH) often visualized as the hydrogen‐terminated C_20_ cap of C_60_ (Figure 1a).^[1]^ The ground‐breaking synthesis of corannulene by Lawton and Barth^[2]^ stimulated a spate of investigations into its structural, electrochemical and photophysical properties. These include bowl‐to‐bowl inversion dynamics,^[3]^ a relative large dipole moment of 2.1 D,^[4]^ akin to that of pyridine (2.2 D),^[5]^ and a moderately deep LUMO of −2.65 eV,^[6]^ similar to that of terephthalonitrile (ca. −2.55 eV).^[7]^ As the availability of corannulene derivatives become more widely available, they have found use as components in energy storage devices,^[8]^ solar cells,^[9]^ organic field‐effect transistors^[10]^ and biomedical applications.^[11]^ However, there are to date only a handful of reports describing the luminescent properties of corannulene‐based compounds,^[12]^ despite the fact that more than 1000 publications have focused on corannulene since the first synthesis of corannulene was reported in 1966 (Figure S1).^[2]^

Corannulene phosphorescence at 77 K had been observed and its radical anion characterized.[^12a^, ^13^] While there is no question these seminal studies serve as the stepping stones to corannulene materials studies seen today there was a period 1971–91 where corannulene studies were few and mostly theoretical in scope.^[14]^ Scott's flash vacuum pyrolysis synthesis^[15]^ brought a resurgence to the field along with solution methods, which allowed substantially more to be investigated into the electroluminescence and photoluminescence of corannulene and derivatives. An early comparison of C_70_ and corannulene low‐temperature phosphorescence (LTP) suggested multi‐state emission,^[16]^ an uncommon “violation” of Kasha's rule. Previous reports demonstrate that mono‐ through pentakis‐acetylene‐bridged derivatives revealed the high photoluminescence quantum yields, Φ_PL_, and various colors of corannulene fluorescence were possible.[^12b^, ^12d^, ^12e^, ^12f^, ^12g^] These ethynyl‐ and aryl‐substituted corannulene derivatives are all fluorescent, thus are not adept at harvesting triplet excitons and so less relevant materials for electroluminescent devices. In 2018, Hatakeyama et al. reported a π‐extended B_2_N_2_‐embedded corannulene derivative (λ_PL_=424 nm; Φ_PL_=69 % in CH_2_Cl_2_) and the first reported example of a corannulene‐based organic light‐emitting diode (OLED), which showed a maximum external quantum efficiency (EQE_max_) of 2.61 %.[^13a^, ^17^] A growing database of crystal structures of corannulene derivatives showed that quasi‐columnar packings, also in polar space groups, were possible. Nonetheless, broad‐scale screening of corannulene materials can now follow after the kilogram‐scale synthesis of corannulene;^[18]^ as well as a robust set of multi‐gram‐scale procedures to prepare mono‐, di‐, tetra‐ and pentahalocorannulenes. Despite the increased interest in the development of corannulene derivatives, there are no examples of luminescent corannulene derivatives that harvest triplet excitons towards their emission such as via thermally activated delayed fluorescence (TADF) and room‐temperature phosphorescence (RTP).

RTP is the radiative transition from molecular triplet excitons at room temperature, together with a phosphorescence lifetime, τ_Ph_, that is typically on the order of microseconds or even longer. OLEDs with RTP emitters are able to harvest 100 % of the excitons to produce light. The majority of reported RTP luminophores are organometallic complexes, where the metals, such as platinum (Pt) and iridium (Ir), can effectively enhance spin‐orbit coupling and thus boost both intersystem crossing (ISC) and radiative emission rates from the triplet manifold.^[19]^ Nevertheless, it remains a significant challenge to develop high‐performance purely organic RTP emitters, as in these compounds that have a large energy gap (ΔE_ST_) between the lowest singlet (S_1_) and the lowest (T_1_) triplet excited states there is weak spin‐orbit coupling between singlet and triplet states.^[20]^ To date, the most popular organic RTP design paradigms mainly follow two strategies: 1) boosting ISC by, for instance, introducing carbonyl groups^[21]^ and/or halogens^[22]^ into a molecule; 2) suppressing triplet nonradiative processes through, for instance, crystallization,^[23]^ supramolecular assembly,^[24]^ or matrix rigidification.^[25]^ Notably, most RTP emitters generally obey Kasha's rule, where phosphorescence originates from T_1_,^[26]^ and the observation of multiple phosphorescence in a single molecule, a violation of Kasha's rule, has rarely been documented.[^21a^, ^23c^, ^27^] The observation of multiple phosphorescence provides insight into high‐lying triplet excited‐state dynamics, as we documented for a naphthalene‐based system.^[28]^ Recently, Li et al. reported a host–guest system with dual phosphorescence, originating from T_n_ (n≥2) and T_1_ states of BPBF_2_.^[27f]^ He et al. reported several benzothiophene‐based crystalline samples that show dual phosphorescence due to the relatively large T_2_‐T_1_ energy gap leading to two distinctive triplet radiative rates.^[27b]^ Dual phosphorescence was also observed in TADF compounds at low temperatures, such as DPTZ‐Me‐DBT reported by Huang et al.^[29]^ and PhCz‐O‐DiKTa and PhCz‐DiKTa reported by us.^[30]^

The present study focuses on the design of molecular donor–acceptor (D‐A) composites based on tetra‐donor‐substituted corannulenes with phenylene bridges to TCzPhCor, TDMACPhCor, and TPXZPhCor (Figure 1b). We demonstrated utility of these RTP materials in an afterglow OLED and for optical temperature sensing stemming from a change in the ratio of multiple phosphorescence yields as a function of temperatures. These results demonstrate the promise of corannulene materials to define a new structure space for optoelectronic materials useful for modern device technology and engineering.

## Results and Discussion

### Synthesis and structural characterization

The compounds TCzPhCor, TDMACPhCor, and TPXZPhCor were synthesized in greater than 55 % yield via the Suzuki–Miyaura cross‐coupling reaction using 1,2,7,8‐tetrabromocorannulene (TBrCor) as the core (outlined in Scheme S1).^[18]^ The identity and purity of the three compounds were verified by ^1^H & ^13^C nuclear magnetic resonance (NMR) spectroscopy, melting point determination, high‐resolution mass spectrometry, elemental analyses, and high‐performance liquid chromatography (HPLC) (Figures S2–S28).

### Single‐crystal analysis

**Figure 2:**
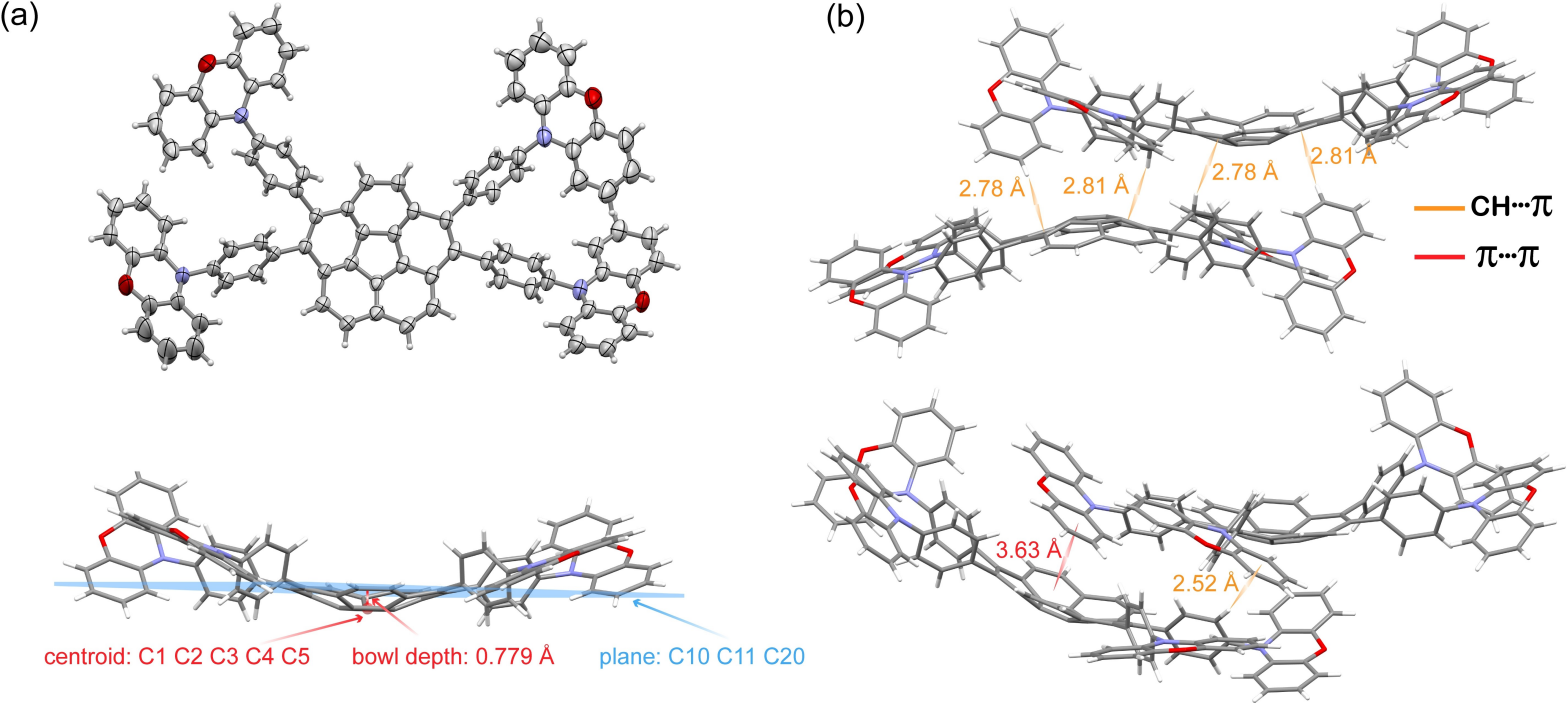
(a) Thermal ellipsoid (top) and stick (bottom) plots of one independent molecule in the single crystal structure of TPXZPhCor. Ellipsoids are drawn at the 50 % probability level, solvent molecules and the minor component of disorder have been omitted for clarity. (b) TPXZPhCor dimers in convex‐convex and convex‐concave orientations along with relevant intermolecular interactions.

X‐ray quality single crystals of TPXZPhCor and commercially available 1,2,5,6‐tetraphenyldibenzo[*ghi,mno*]fluoranthene (TPhCor) were obtained from slow evaporation of toluene at room temperature. As shown in Figure 2a, TPXZPhCor adopts a twisted D‐A geometry, which reduces the likelihood of the occurrence of π‐π interactions (see also Figures S29 and S30, Table S1). The bowl depth, described as the perpendicular distance from the plane designated by the peripheral ten carbon atoms of the corannulene skeleton to the parallel plane containing the hub C1–C5 ring, is 0.779 Å (Figure 2a bottom). This is shallower than that of 1,2,5,6‐tetraphenyldibenzo[*ghi,mno*]fluoranthene (TPhCor) (0.886 Å, Figure S31) and corannulene (0.875 Å).^[14b]^ Figure 2b illustrates the packing motifs of TPXZPhCor dimers in the unit cell. The closest packing corannulenes are arranged in dimers, in a convex‐convex manner (Figure 2b, top). Two sets of CH⋅⋅⋅π interactions support the dimers, with associated H⋅⋅⋅centroid distances of 2.78 (phenylene to corannulene) and 2.81 Å (phenoxazine to corannulene), which may contribute to the suppression of nonradiative excitonic transitions. Neighboring molecules disposed in a relative concave‐convex orientation also show weak CH⋅⋅⋅π (phenylene to phenoxazine, H⋅⋅⋅centroid distance of 2.52 Å) and π⋅⋅⋅π (corannulene to phenoxazine, centroid⋅⋅⋅centroid distance of 3.63 Å) interactions. Due to the steric bulk of the PXZ groups, adjacent molecules pack in an offset manner with the shortest distance between the centroids of the central cyclopentadiene ring of the corannulenes being 7.83 Å (Figure S32). To best of our knowledge, corannulene derivatives generally exhibit convex‐concave stacking[^12c^, ^31^] or “clamshell” type packing.[^12d^, ^32^] The picture of the packing motif observed in TPXZPhCor is slightly different. The shortest equivalent distance between adjacent TPXZPhCor molecules arranged in a concave‐convex manner is 11.80 Å, which is longer than those documented in previous reports,[^12b^, ^12c^, ^31^] and in a concave‐concave manner is 16.07 Å (Figure S32).

### Theoretical modelling

**Figure 3:**
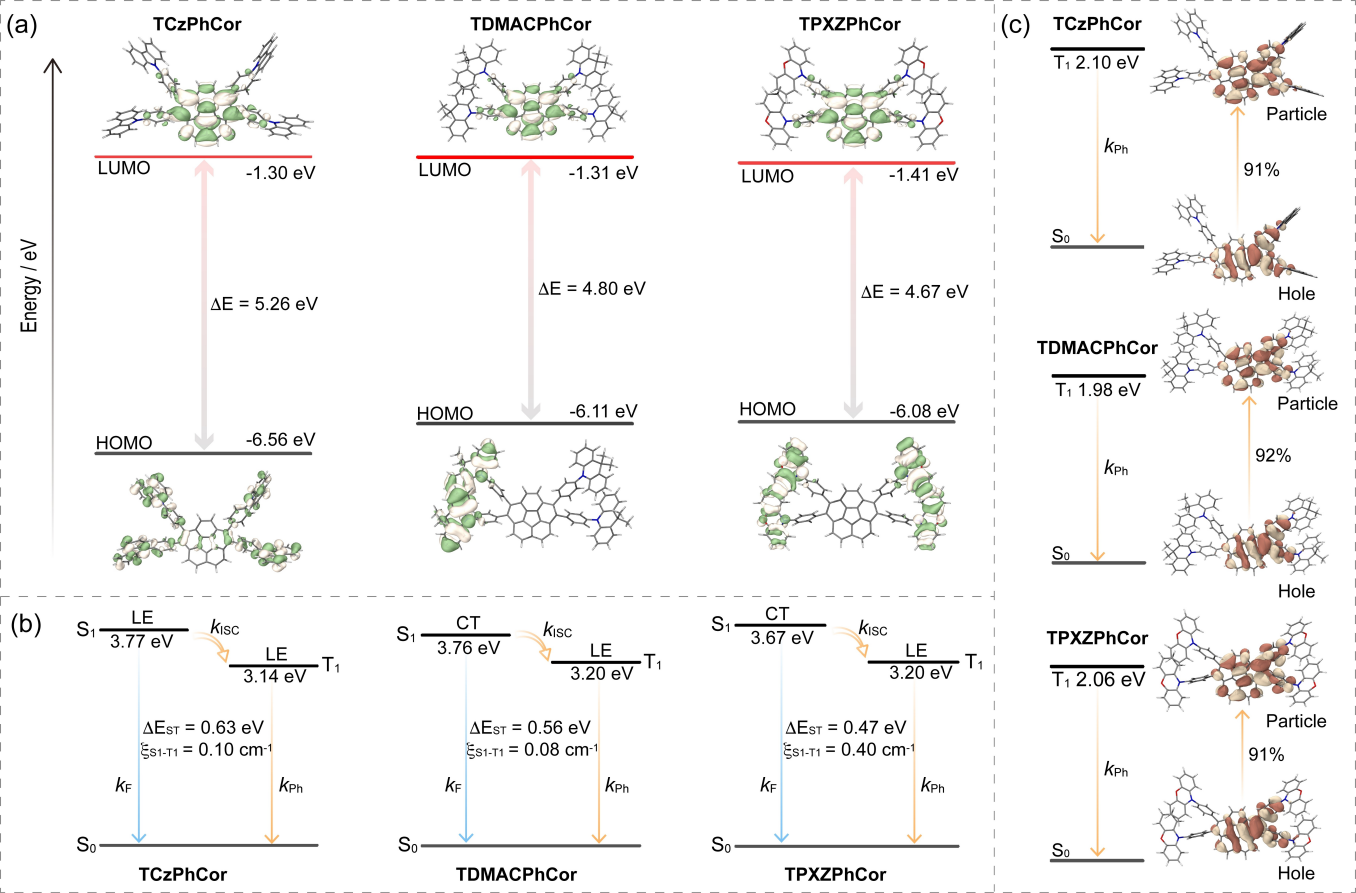
(a) Electron density distribution and energy levels of frontier molecular orbitals (isovalue: 0.02). (b) Vertical excitation energy levels calculated at the optimized S_0_ geometry in the gas phase at the TDA‐DFT−M062X/6‐31G(d,p) level. (c) Natural transition orbitals (isovalue: 0.02) and T_1_ vertical emission energy levels at the TDA‐DFT‐M062X/6‐31G(d,p) level.

We first modelled the photophysical properties of the three compounds in the gas phase using time‐dependent density functional theory (TD‐DFT) within the Tamm‐Dancoff approximation (TDA)^[33]^ at the TDA‐DFT‐M062X/6‐31G(d,p) level of theory.^[34]^ At the optimized ground‐state (S_0_) geometries, the bowl shape of corannulene is present in all three compounds (Figure S33). The bowl depth slightly decreases as the electron‐donating strength of the donors increases from 0.84 Å (TCzPhCor) to 0.83 Å (TDMACPhCor) and 0.79 Å (TPXZPhCor), all shallower than those of corannulene (0.88 Å) and TPhCor (0.84 Å). The calculated bowl depths of TPhCor and TPXZPhCor coincide well with those of the single crystal structures (Figures 2a and S33). Strongly twisted D‐A geometries were computed for TCzPhCor, TDMACPhCor, and TPXZPhCor (Figure S34). Larger dihedral angles between the donor and the phenylene bridge exist in TDMACPhCor (from 84.9° to 94.8°) and TPXZPhCor (from 81.0° to 95.7°) compared to TCzPhCor (from 51.0° to 52.5°), while the dihedral angles between the phenylene bridge and corannulene slightly increase in TDMACPhCor (from 62.6° to 65.8°) and TPXZPhCor (from 59.3° to 79.8°) than TCzPhCor (from 53.3 to 61.3°). The S_0_ geometry of TPXZPhCor is similar to that found in the single crystal (Figure 2a), where the donor groups are highly twisted with respect to the phenylene bridge and the phenylene bridge is itself strongly twisted with respect to corannulene (Figures S34). The calculated energies of the highest occupied molecular orbital (HOMO) and lowest unoccupied molecular orbital (LUMO) are displayed in Figures 3a and S35. As expected, the HOMO and LUMO are localized on the donors and the corannulene acceptor, respectively. The progressively destabilized HOMO reflects the increasing electron‐donating strength of the donors in TCzPhCor, TDMACPhCor, and TPXZPhCor, respectively, while similar LUMO energies reveal negligible electronic coupling between the donors and the corannulene acceptor. The LUMO of TPhCor is destabilized (−0.95 eV) compared to the LUMOs of the three corannulene derivatives (Figure S36) as it has a smaller conjugation length.

Natural transition orbital (NTO) analyses at the S_0_ geometry provide insight into the nature of excited‐state transitions (Figure S37). For TCzPhCor, the S_1_ state possesses a locally excited (LE) character mostly centered on the corannulene core. However, for TDMACPhCor and TPXZPhCor, the S_1_ state is of charge‐transfer (CT) character, reflective of the stabilization of this state compared to the LE states in the presence of the stronger electron donors; indeed, there is a progressive, although very weak, stabilization of the S_1_ state from 3.77 eV in TCzPhCor to 3.76 eV in TDMACPhCor and 3.67 eV in TPXZPhCor as a result of the near orthogonal conformation of the donor groups relative to the TPhCor. The ΔE_S1T1_ values also decrease progressively from 0.63 eV in TCzPhCor to 0.56 eV in TDMACPhCor and 0.47 eV in TPXZPhCor (Figure 3b).

The S_1_ and T_1_ geometries were optimized at the TDA‐DFT−M062X/6‐31G(d,p) level. TCzPhCor exhibits a relatively larger geometry relaxation, where the root‐mean‐square displacement (RMSD) value, calculated using the VMD program,^[35]^ is 0.60 Å between S_0_ and S_1_ compared to that of TDMACPhCor (0.48 Å) and TPXZPhCor (0.48 Å) (Figure S38). While TDMACPhCor shows a quite large RMSD value of 0.92 Å between S_0_ and T_1_, TCzPhCor and TPXZPhCor possess the same value of 0.77 Å. The bowl depth of corannulene in both the S_1_ (bowl depth=≈0.68 Å) and T_1_ (bowl depth=≈0.63 Å) geometries decreases by more than 15 % compared to that in the S_0_ geometry (bowl depth=≈0.82 Å) for all three compounds (Figures S33 and S39). At the relaxed S_1_ geometry, there is a much larger S_1_‐T_1_ spin‐orbit coupling (SOC) matrix element (0.40 cm^−1^) in TPXZPhCor than that in both TCzPhCor (0.10 cm^−1^) and TDMACPhCor (0.08 cm^−1^) (Figures 3b and S40), while larger SOC values of greater than 0.56 cm^−1^ are noted from S_1_ to T_3_ (Figure 3b). The density of triplet states and the SOC between these and S_1_ should favor ISC, which should be beneficial for producing triplet excitons for RTP. At the optimized T_1_ geometry (Figure S34), TCzPhCor, TDMACPhCor, and TPXZPhCor possess similar T_1_ energies (Figure 3c), which indicate the same LE character of the T_1_ state on the corannulene core (Figure S36). The relatively stabilized T_1_ energy (Figure 3c) of TDMACPhCor probably originates from an LE state that extends onto the phenylene rings, which is possible due to the smaller dihedral angles between the corannulene and the π‐linker than exists in TCzPhCor and TPXZPhCor (Figure S34).

### Optoelectronic investigations

The energies of the frontier molecular orbitals (FMOs) were inferred from the electrochemical behavior of TCzPhCor, TDMACPhCor, and TPXZPhCor using cyclic voltammetry (CV) and differential pulse voltammetry (DPV) in degassed dichloromethane (DCM), with tetra‐*n*‐butylammonium hexafluorophosphate, [^*n*^Bu_4_N]PF_6_, as the supporting electrolyte (Figure S41). The oxidation potentials (*E*_ox_), determined from the DPV peak values, are 1.29 V (TCzPhCor), 0.81 V (TDMACPhCor), and 0.73 V (TPXZPhCor) versus SCE. The corresponding HOMO energies are −5.64 eV, −5.13 eV, and −5.07 eV for TCzPhCor, TDMACPhCor, and TPXZPhCor, respectively, consistent with the trend in calculated HOMO energies (Figure 3a) and reflecting the increasing strength of the donor across the series. TDMACPhCor and TCzPhCor have similar E_red_ of −2.02 and −1.94 V, respectively, which align well with their similar LUMO energies (Figure 3a). However, the E_red_ of TPXZPhCor (−1.78 V) is anodically shifted, in line with the DFT prediction, which we ascribe to a more delocalized electronic distribution of the LUMO on the corannulene and the phenylene linker. The corresponding LUMO energies of TCzPhCor, TDMACPhCor, and TPXZPhCor are −2.40 eV, −2.32 eV, and −2.56eV. The resulting HOMO–LUMO gaps of TCzPhCor, TDMACPhCor, and TPXZPhCor are 3.24, 2.81, and 2.51 eV, respectively, mirroring the trend in the calculated values (Figure 3a).

**Figure 4:**
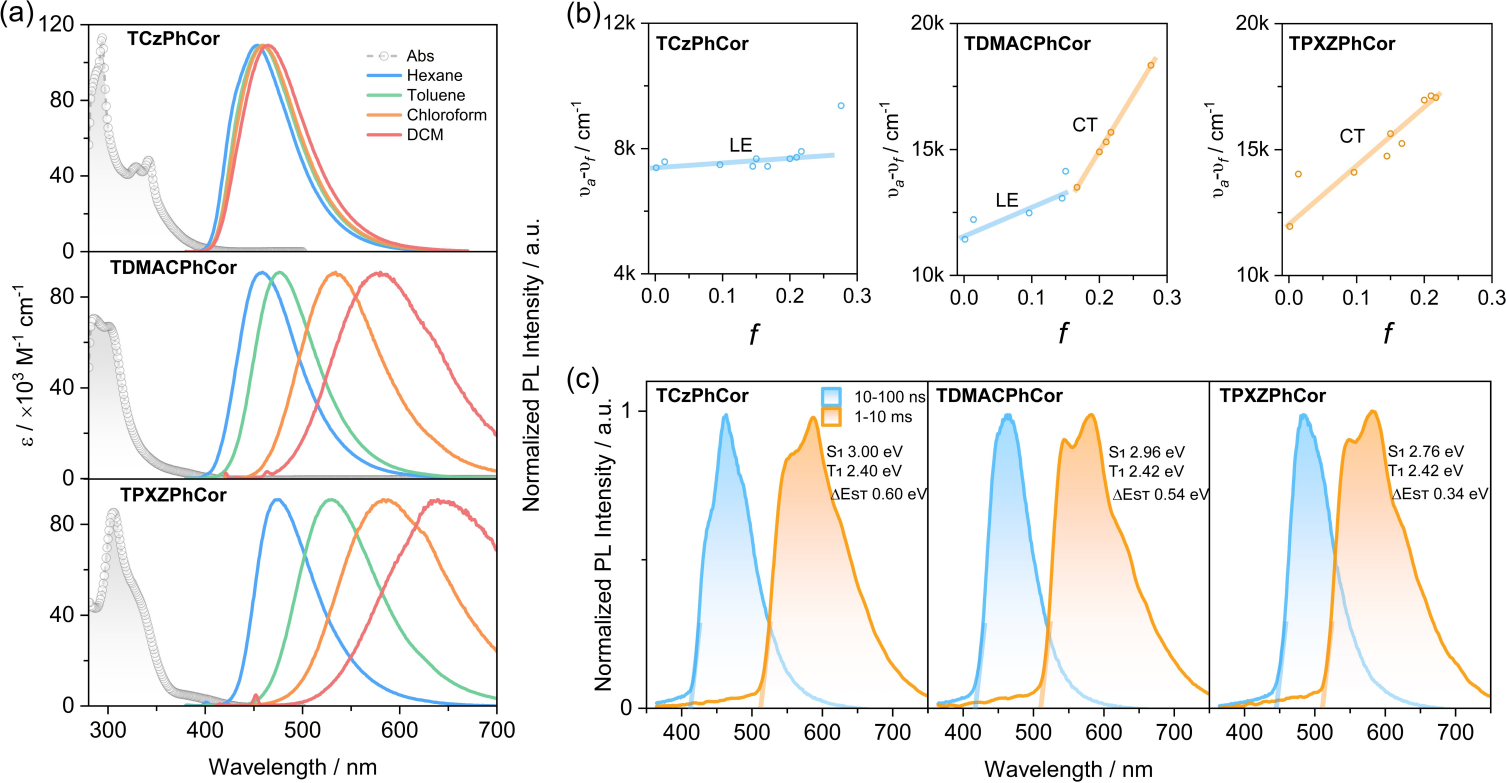
(a) UV/Vis absorption (in toluene) and solvatochromism studies of TCzPhCor, TDMACPhCor, and TPXZPhCor recorded in air at room temperature (λ_exc_=340 nm). (b) Lippert–Mataga plots of TCzPhCor, TDMACPhCor, and TPXZPhCor. (c) Prompt fluorescence (1–100 ns) and phosphorescence spectra (1–10 ms) recorded in toluene at 77 K of TCzPhCor, TDMACPhCor, and TPXZPhCor (λ_exc_=343 nm).

The UV/Vis absorption spectra of the three emitters measured in toluene show a strong absorption centered at ≈300 nm (Figures 4a, S42 and Table S2), originating from the LE π‐π^*^ transitions on the corannulene core.^[36]^ A weak CT transition band at around 400 nm is observed in TDMACPhCor and TPXZPhCor (Figure S42a–b). The enhanced molar absorptivity coefficients of the band at about 300 nm, for all three compounds compared to both TPhCor and corannulene, are the result of contributions from LE transitions on the donors (Figures S42a–b).^[37]^ The optical band gaps, calculated from the intersection point of the normalized absorption and emission spectra, for TCzPhCor, TDMACPhCor, and TPXZPhCor are 3.04 eV, 3.01 eV, 2.90 eV, respectively (Figure S42c).

Steady‐state photoluminescence (PL) spectra recorded in toluene show a gradual red‐shift from TCzPhCor to TDMACPhCor and TPXZPhCor, coinciding with their decreasing optical gaps (Figure S42c). A stronger positive solvatochromism is observed in TPXZPhCor compared to TDMACPhCor (Figure 4a), which is almost absent in TCzPhCor. As shown in Figure 4b, the Lippert–Mataga study reveals that the emissive state of TCzPhCor is of dominant LE character, while the emissive state of TDMACPhCor is better described as having hybrid LE and CT character, that of TPXZPhCor is of CT character, matching well with the calculations (Figure 3b and S37). The related experimental absorption and PL spectra and calculated parameters from the Lippert–Mataga study are presented in Figure S43 and Table S3. Notably, a linear relationship between the Stokes shift (υ_a_‐υ_f_) and the orientational polarizability *f* was observed for TCzPhCor and TPXZPhCor (Figure 4b). The relatively small slope for TCzPhCor and unstructured emission imply that the S_1_ state possesses a moderate degree of CT character, reflected in a dipole moment, *μ*_e_, of 11.4 D calculated according to the Lippert–Mataga equation (see Supporting Information for details). For TPXZPhCor, the slope of the Lippert Mataga plot is much steeper, which implies a much stronger CT character to the S_1_, linked with an associated *μ*_e_ of 24.7 D. The picture for TDMACPhCor is more complex as the Lippert–Mataga plot reveals two distinct regimes with *μ*_e_ of 16.9 D and 33.0 D. Such behavior is characteristic of an excited state of mixed LE and CT character (HLCT).^[38]^ The Φ_PL_ values of TCzPhCor, TDMACPhCor, and TPXZPhCor in aerated toluene are 34 %, 18 %, and 16 %, respectively, which essentially do not change in degassed toluene (Table S2). The time‐resolved PL decays are monoexponential, with PL lifetimes, τ_PL_, in the nanosecond regime (Figure S44). The Φ_PL_ and τ_PL_ values rule out emission involving triplet excitons. The Δ*E*_ST_ for TCzPhCor, TDMACPhCor, and TPXZPhCor, determined from the onsets of prompt and delayed emission in toluene at 77 K, are 0.59 eV, 0.54 eV, and 0.34 eV (Figure 4c), respectively. Such large Δ*E*_ST_ values rule out the possibility of TADF. The similar phosphorescence spectra in the three compounds recorded in toluene at 77 K indicate that the nature of the T_1_ state is the same and localized on the TPhCor core (Figure S45).

**Figure 5:**
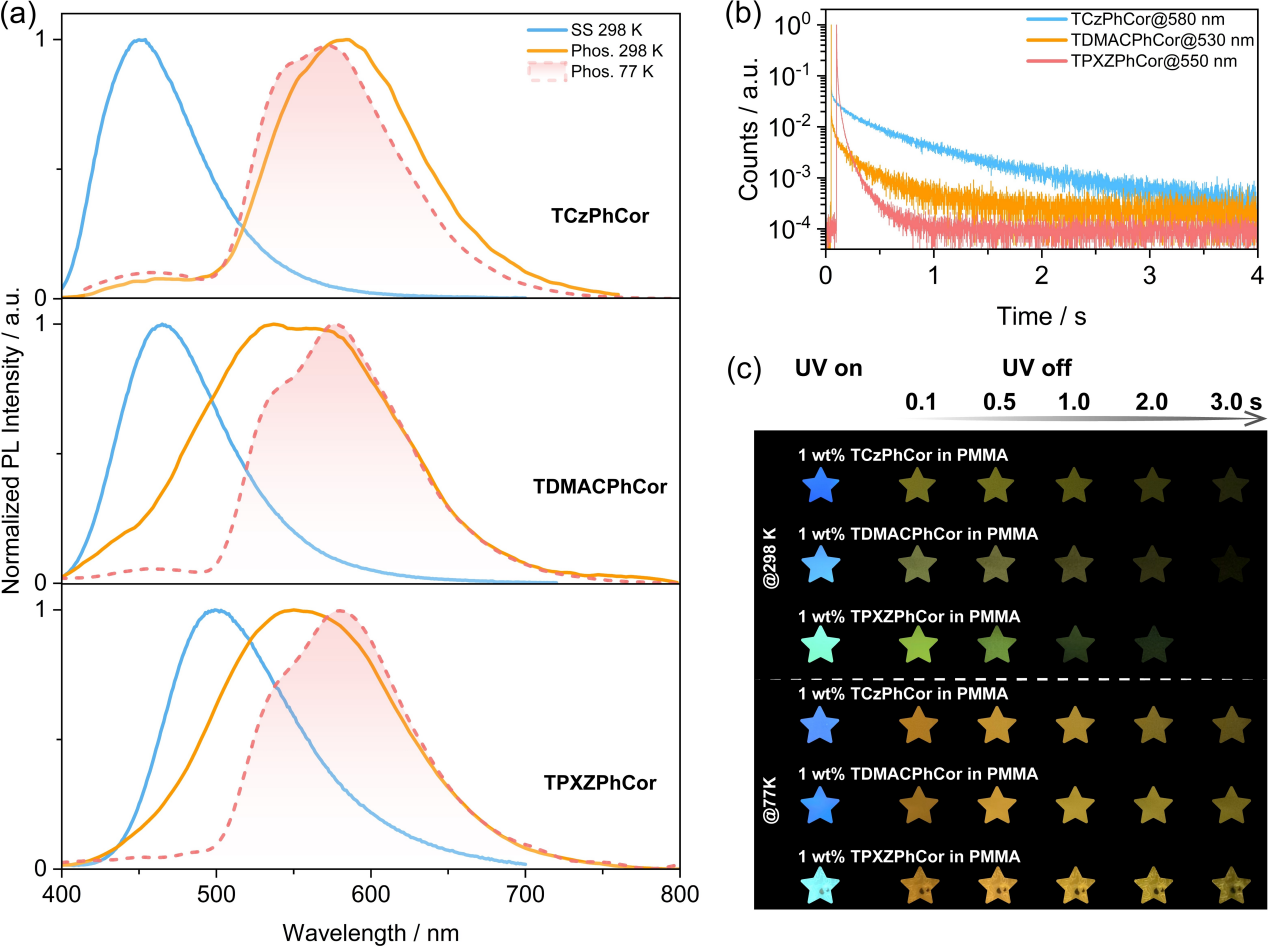
(a) Steady‐state and time‐gated (time‐gated window: 30–200 ms) PL spectra of 1 wt % TCzPhCor, 1 wt % TDMACPhCor, and 1 wt % TPXZPhCor in PMMA at 298 K and 77 K (λ_exc_=320 nm). (b) Time‐resolved PL decay profiles of 1 wt % TCzPhCor, 1 wt % TDMACPhCor, and 1 wt % TPXZPhCor in PMMA under vacuum. (c) Images of the PMMA films with emitters showing afterglows at 298 K and 77 K under vacuum (excitation source: 365 nm UV torch).

We next investigated the photophysical properties of the three emitters in PMMA at a 1 wt % doping concentration. As shown in Figure 5a, the steady‐state PL spectra of TCzPhCor, TDMACPhCor, and TPXZPhCor show structureless emission at λ_PL_ of 450 nm, 465 nm, and 500 nm, respectively, with associated τ_PL_ of 9.8 ns, 25.2 ns, and 30.8 ns, respectively (Figure S46). RTP spectra of TCzPhCor, TDMACPhCor, and TPXZPhCor were acquired across a time‐gated window of 30–200 ms, with maxima, λ_Ph_, centered at around 580 nm, 530 nm, and 550 nm, respectively. The corresponding phosphorescence lifetimes (τ_Ph_) are 573.0 ms, 286.1 ms, and 34.6 ms, respectively (Figure 5b). The LTP spectra at 77 K are centered at around 575 nm, 576 nm and 580 nm for TCzPhCor, TDMACPhCor, and TPXZPhCor, respectively (Figure 5a). The LTP emission profiles overlap very well with the phosphorescence spectrum of 1 wt % TPhCor in PMMA measured at 77 K (Figure S47). Based on these results, we can conclude that the LTP originates from the LE T_1_ state localized on TPhCor. Notably, there is a remarkable difference in the phosphorescence spectra at 298 K and 77 K of both TDMACPhCor and TPXZPhCor (Figures 5a), also reflected in the different phosphorescence afterglows at 298 K and 77 K (Figures 5c). Based on similar observations in our previous report,^[28]^ we assign this dual phosphorescence to T_1_^H^ and T_1_^L^, associated with phosphorescence from two conformers. Considering the broad RTP of TDMACPhCor and TPXZPhCor, we contend that the spectra consist of contributions from both T_1_^H^ and T_1_^L^, which is only possible if there is thermally activated excitonic coupling between T_1_^H^ and T_1_^L^ (see below)_._

**Figure 6:**
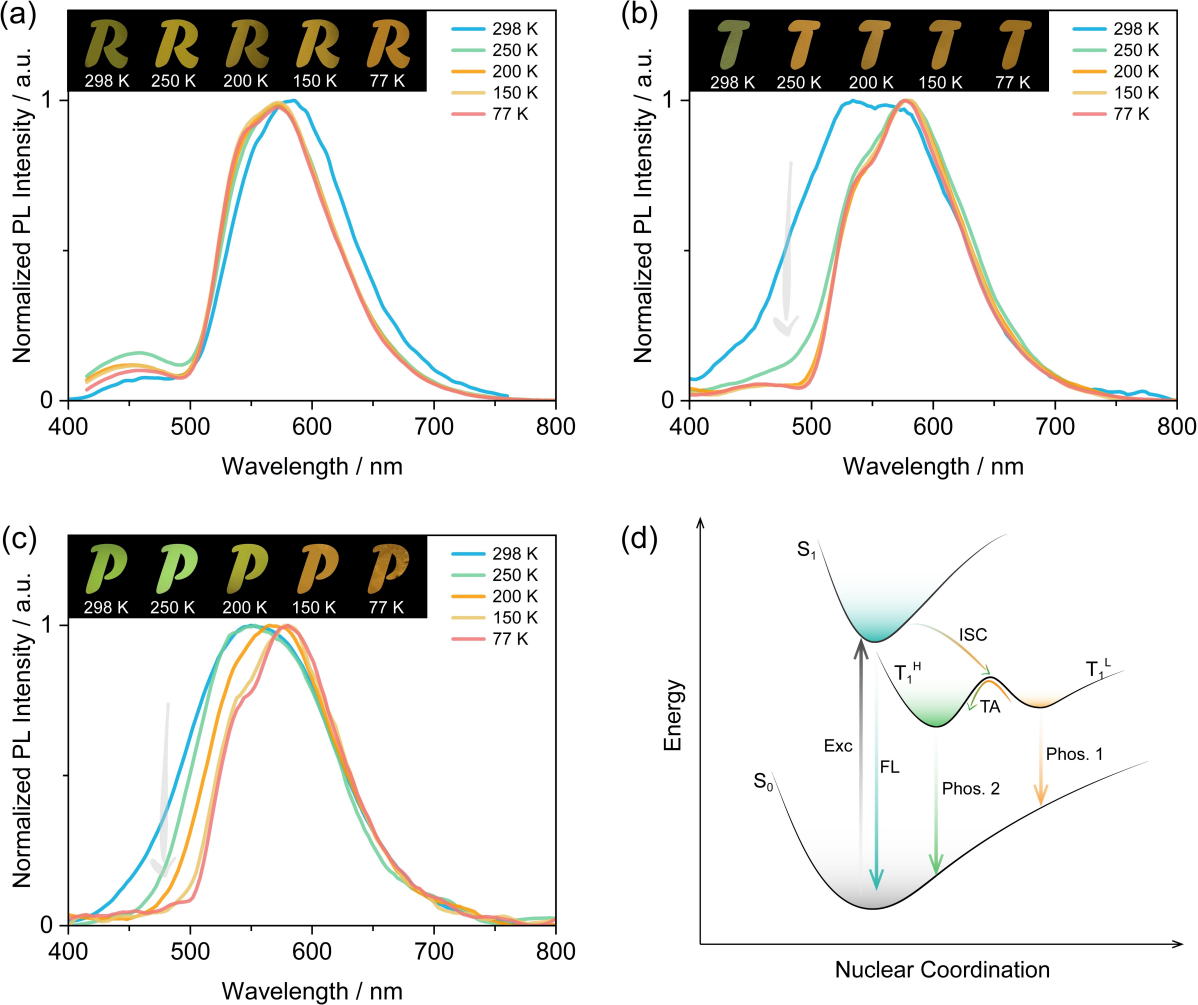
Temperature‐dependent phosphorescence spectra of (a) 1 wt % TCzPhCor, (b) 1 wt % TDMACPhCor, and (c) 1 wt % TPXZPhCor in PMMA (time‐gated window: 30–200 ms, λ_exc_=320 nm; insets: images showing phosphorescence in vacuum at different temperatures, excited by a 365 nm UV torch; The images of the letters were generated using a mask on top of the image of the photoexcited film on the postprocessed image. (d) Mechanistic illustration of dual phoshorescence using a simplified Jablonski diagram; Exc: excitation; FL: fluorescence; TA: thermal activation; Phos.: phosphorescence.

To validate this hypothesis, temperature‐dependent phosphorescence spectra were measured from 77 to 298 K. As shown in Figure 6a, the phosphorescence emission of TCzPhCor shows a negligible change across this temperature range; the weak emission band centred at ≈460 nm is ascribed to steady‐state fluorescence due to the residual background excitation, evidenced by the unchanged PL intensity as a function of temperature (Figure S48a). A similar background excitation was observed in the phosphorescence spectrum of the TCzPhCor‐doped PMMA film (Figure 5a). However, for TDMACPhCor and TPXZPhCor, distinctly red‐shifted phosphorescence spectra were obtained with decreasing temperature, also reflected by the change in the phosphorescence afterglows (Figures 6b‐c, S48d‐f), which is the result of loss of the phosphorescence from T_1_^H^. Figure 6d illustrates a plausible mechanistic explanation, where excitonic coupling between T_1_^H^ and T_1_^L^ is temperature dependent, which explains why at different temperatures, differing phosphorescence afterglows were recorded. To elucidate the mechanism, we also conducted relaxed potential energy surface calculations based on the optimized T_1_ geometry at the uM06‐2X/6‐31G(d,p) level (Figure S49). Notably, it is not feasible to conduct such calculations on these molecules given their size, and so to simplify the calculations, mono‐substituted corannulenes were taken as examples. As demonstrated in our previous work,^[28]^ the conformational energy barrier is the key to determining which of the two geometries (T_1_^H^ and T_1_^L^) dominates and likely contributes the most to the phosphorescence emission (see details shown in Figure S49). The energy differences between T_1_^H^ and T_1_^L^ approximated from the onsets of the respective phosphorescence spectra are 0.36 eV, and 0.31 eV for TDMACPhCor, and TPXZPhCor, respectively (Figure S50).

**Figure 7:**
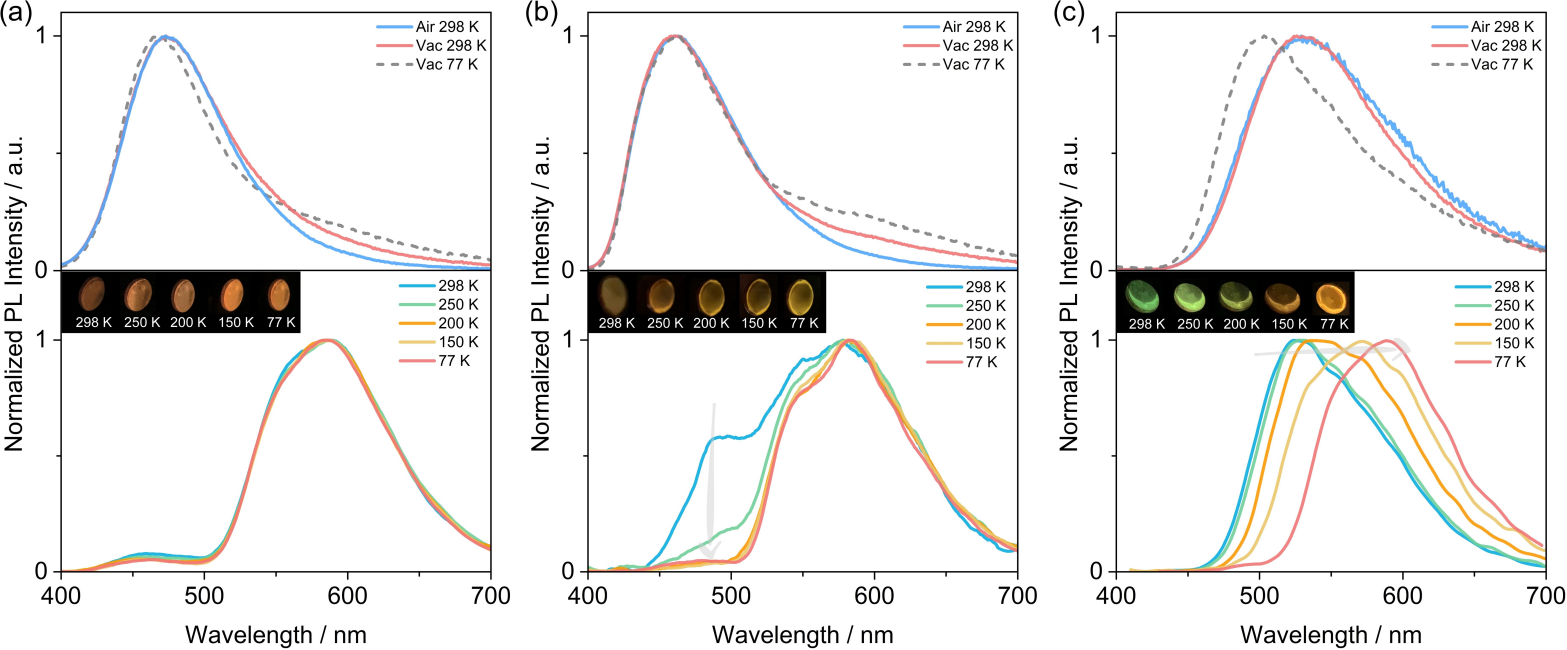
Steady‐state PL (at 298 K and 77 K) and temperature‐dependent phosphorescence spectra of (a) 1 wt % TCzPhCor, (b) 7 wt % TDMACPhCor and (c) 15 wt % for TPXZPhCor in mCP (λ_exc_=320 nm; time‐gated window: 30–200 ms; insets: images showing phosphorescence at different temperature excited by a 365 nm UV torch).

With a potential view to assessing these materials in OLEDs, we explored the photophysics in doped films in mCP as this host has a T_1_ energy of ≈3.0 eV,^[39]^ thus excitons would be confined onto the RTP emitters. The optimized doping concentrations of TCzPhCor, TDMACPhCor, and TPXZPhCor are 1 wt %, 7 wt %, and 15 wt %, respectively, where the Φ_PL_ values are 37.2 %, 32.1 %, and 42.5 %, respectively. The steady‐state PL spectra of the three emitters in air and vacuum at 298 K show broad and structureless emission at λ_PL_ of 465 nm, 475 nm, and 530 nm (Figures 7a–c, top), with τ_PL_ of 6.5 ns, 19.8 ns and 30.7 ns, for TCzPhCor, TDMACPhCor and TPXZPhCor, respectively (Figure S51). Similar to the result recorded in PMMA, TCzPhCor in mCP exhibits RTP at 588 nm with τ_Ph_ of 293.7 ms (Figure S52a), which originates mostly from T_1_^L^ that is localized on the TPhCor core. The unchanged phosphorescence spectra as a function of temperature are consistent with a single origin of the phosphorescence (Figure 7a, bottom). For TDMACPhCor, dual RTP emission from T_1_^H^ and T_1_^L^ was observed, centered at 480 nm and 585 nm Figure 7b, bottom) with associated τ_Ph_ of 96.8 ms and 343.3 ms (Figures S52b and S53), respectively. As the temperature decreases, the high‐energy phosphorescence from T_1_^H^ decreases in intensity as it can no longer be efficiently populated. For TPXZPhCor, the emission at 528 nm is dominated by the RTP from T_1_^H^ (Figure 7c, bottom), with a τ_Ph_ of 82.6 ms (Figure S52c). This is reflected by a significant change in the spectral profile as a function of temperature (Figures 7a–c and S54). The Φ_RTP_ of TCzPhCor, TDMACPhCor and TPXZPhCor in mCP are 2.6 %, 2.7 %, and 18.0 %, respectively, which are much higher than those measured in 1 wt % doped PMMA films (0.1 % for TCzPhCor, 0.3 % for TDMACPhCor and 10.0 % for TPXZPhCor, respectively) (Figure S52d). This divergence in Φ_RTP_ recorded in mCP and PMMA results from different host–guest interactions, which provides a window into an effective strategy to regulate the T_1_^H^ and T_1_^L^ excitonic coupling.

**Figure 8:**
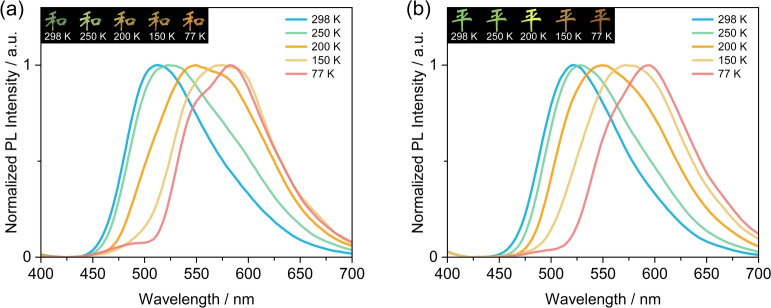
Temperature‐dependent phosphorescence spectra of (a) 1 wt % and (b) 30 wt % TPXZPhCor in mCP films (time‐gated window: 30–200 ms; λ_exc_=320 nm); inset: images showing phosphorescence in vacuum at different temperatures, where the two Chinese characters together mean “peace”. The characters were generated using a mask on top of the image of the photoexcited film excited by a 365 nm UV torch, on the postprocessed image.

Given that TPXZPhCor shows dual phosphorescence emission in mCP as a function of temperature (Figure 7), we decided to study the photophysics of TPXZPhCor in mCP at both lower (1 wt %) and higher (30 wt %) doping concentrations to interrogate the influence of intermolecular interactions on the dual phosphorescence behavior. The Φ_PL_ values measured at these doping concentrations indicate that the 30 wt % TPXZPhCor doped mCP film possesses the highest RTP Φ_PL_ (17.8 %), higher than that of the 1 wt % doped film (RTP Φ_PL_=10.2 %) (Figures S55a–b). The observed aggregation‐induced phosphorescence enhancement is hypothesized to originate from the twisted geometry, which disfavors unwanted intermolecular π‐π interactions (Figure S32). Both 1 wt % and 30 wt % TPXZPhCor doped mCP films exhibit similar temperature‐dependent phosphorescence behavior to the 15 wt % doped sample. As temperature decreases, there is a red shift of the phosphorescence, reflected in the temperature‐dependent phosphorescence afterglows (Figures 8a–b) and evolution in the CIE coordinates (Figures S55c–d). The photophysical investigation at these two doping concentrations indicates that aggregation has negligible influences on the T_1_^H^ and T_1_^L^ phosphorescence spectra, which implies that the phosphorescence occurs from monomolecular species and not aggregates.

### Temperature sensing and afterglow OLEDs

**Figure 9:**
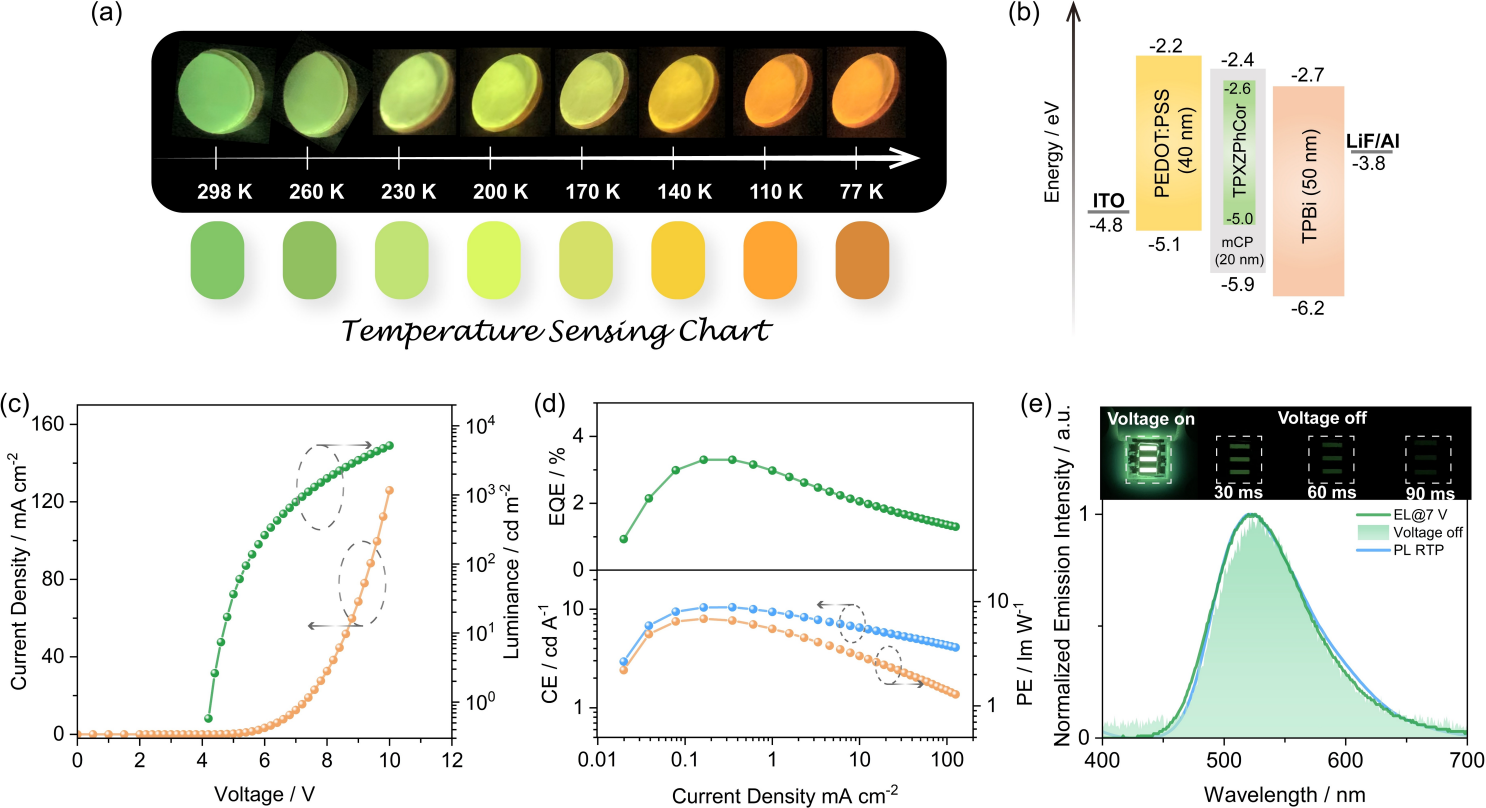
(a) Temperature‐dependent phosphorescence afterglows of 30 wt % TPXZPhCor doped films in mCP and fitted sensing charts. (b) Device configuration and energy level diagram of the materials employed in the devices. (c) Current density‐voltage‐luminance plot. (d) efficiencies‐current density plot. (e) Afterglow EL and a comparison of the EL and PL spectra; inset: photos of the device showing steady‐state and afterglow luminescence under electrical excitation.

In our previous report,^[28]^ we developed several temperature sensing charts showing phosphorescence afterglows ranging from blue to green. Considering the remarkable difference in the phosphorescence afterglows of TPXZPhCor as a function of temperature, the temperature sensing charts using the 30 wt % TPXZPhCor doped mCP films exhibit a much wider color palate than that in our previous study,^[28]^ ranging from cyan at 298 K to orange at 77 K (Figure 9a). To the best of our knowledge, there are only a limited number of reports of temperature sensing based on phosphorescence afterglow materials.^[40]^ Lee et al.^[40a]^ reported a temperature sensor in a microfluidic device based on an organic RTP compound, Br6 A, doped in a temperature‐sensitive polymer matrix; however, no temperature‐dependent emission color change was observed. Qin et al.^[40b]^ demonstrated that a fluorine‐substituted organic phosphor is thermo‐responsive in both crystals and doped films, where the phosphorescence lifetime was found to increases linearly with decreasing temperature; however, no afterglow color change was observed, which limits its application as a rapid naked eye temperature sensor. TPXZPhCor, by contrast, can be used for rapid temperature sensing in cold environments.

We also explored the potential of TPXZPhCor as an emitter in an afterglow OLED. To the best of our knowledge, there were only three prior reports of afterglow OLEDs.^[41]^ Adachi and co‐workers demonstrated the first afterglow OLED wherein they employed a hydrophobic steroid derivative as the host and a deuterated fluorene‐based compound as the dopant in the emissive layer (EML).^[41a]^ The green OLED only showed an EQE_max_ of ≈1 %. The same group proposed another strategy and developed an exciplex‐based afterglow OLED by reducing the guest concentration to slow the exciton recombination rate and by increasing the EML thickness to enhance the charge accumulation process.^[41b]^ However, the abnormally low guest concentration and thick EML contribute to the significantly reduced device brightness and necessitate a very high driving voltage (pulse voltage=70 V), resulting in an EQE_max_ of ≈0.8 %. Further, the device exhibited a long transient EL decay of more than 10 s. Xie et al.^[41c]^ reported an afterglow OLED that used 2,8‐bis(diphenylphosphoryl)dibenzo[b,d]thiophene (PPT) and *N,N′*‐di(1‐naphthyl)‐*N,N′*‐ diphenyl‐(1,1′‐biphenyl)‐4,4′‐diamine (NPB) as host and guest, respectively, within the EML. The devices showed green afterglow emission with an ultralong lifetime of 356 ms; however, the EQE_max_ was 1.47 % and the maximum luminance (L_max_) reached only 743 cd m^−2^.

We fabricated solution‐processed OLEDs employing 15 wt % TPXZPhCor doped in mCP films as the EML and investigated their performance as well as the afterglow produced from the electroluminescence. The device stack consisted of: indium tin oxide (ITO)/poly(3,4‐ethylenedioxy‐ thiophene) : poly(styrenesulfonate) (PEDOT : PSS) (40 nm)/EML (30 nm)/2,2′,2′′‐(1,3,5‐benzinetriyl)‐tris(1‐phenyl‐1‐*H*‐benzimidazole (TPBi) (50 nm)/LiF (1 nm)/Al (100 nm), where ITO and Al serve as the anode and cathode, respectively, PEDOT : PSS acts as the hole‐transporting layer, TPBi acts as the electron‐transporting layer, and LiF acts as the electron‐injection layer (Figure 9b). The afterglow OLED performance is summarized in Table 1. The optimized device has a turn‐on voltage of 4.3 V and a driving voltage at 100 cd m^−2^ of 5.5 V, which is much lower than previously reported afterglow OLEDs.^74^ The L_max_ reached 5167 cd m^−2^ (Figure 9c) and the EQE_max_ was 3.3 % (Figure 9d). Compared to the previous reports of afterglow OLEDs,^[41]^ device A has the highest performance (Table 1). The EL spectrum (Figure 9e) of the OLED matches the phosphorescence spectrum of the 15 wt % TPXZPhCor doped in the mCP film. Furthermore, the steady‐state EL and afterglow spectra (Figure 9e) are essentially the same, reflecting degenerate S_1_ and T_1_^H^ states.

**Table 1:** Summary of afterglow OLED performance.

Devices	V_on_^[a]^/V	V_100_^[b]^/V	λ_EL_^[c]^/nm	λ_EL_^[d]^/nm	Luminance^[e]^/cd m^−2^	EQE_max_^[f]^/%
A	4.3	5.5	525	528	5167	3.30
Ref. [41c]	5.8	8.9	440	544	450	1.47
Ref. [41b]	–	–	–	≈520	–	≈1.00
Ref. [41a]	–	–	–	–	–	≈0.8

[a] Turn‐on voltage at 1 cd m^−2^. [b] Driving voltage at 100 cd m^−2^. [c] EL maximum at 7 V. [d] EL maximum after ceasing electrical excitation. [e] Maximum luminance. [f] Maximum external quantum efficiency.

## Conclusion

Herein, we have systematically explored how the strength of the donor regulates multiple phosphorescence in the corannulene‐based emitters TCzPhCor, DMACPhCor, and PXZPhCor. We found that TCzPhCor shows ultralong RTP from the lowest T_1_, with τ_Ph_ of 573.0 ms (in 1 wt % doped PMMA) and 293.7 ms (in 1 wt % doped mCP). TDMACPhCor, by contrast, exhibits dual RTP in both PMMA and mCP due to the balanced distribution of triplet excitons emanating from T_1_^H^ and T_1_^L^ states, with τ_Ph_ values of 96.8 ms and 343.7 ms, respectively, in 7 wt % doped mCP, while TPXZPhCor only shows RTP from T_1_^H^, with τ_Ph_ of 82.6 ms in 15 wt % doped mCP, which occurs as a result of thermally activated reverse internal conversion. Exploiting this photophysical behavior, we demonstrated how TPXZPhCor can act as an optical temperature sensor in the range from 77 K to 298 K. Benefitting from degenerate S_1_ and T_1_^H^ states, we have fabricated record‐efficient solution‐processed afterglow OLEDs using TPXZPhCor, which showed an EQE_max_ of 3.3 % and a L_max_ of 5167 cd m^−2^.

## Supporting Information

^1^H and ^13^C NMR spectra, HRMS and EA of all target compounds; X‐ray crystallographic details; supplementary computational data; supplementary photophysical data.

## Conflict of interest

The authors declare no competing financial interest.

1

## Supporting information

As a service to our authors and readers, this journal provides supporting information supplied by the authors. Such materials are peer reviewed and may be re‐organized for online delivery, but are not copy‐edited or typeset. Technical support issues arising from supporting information (other than missing files) should be addressed to the authors.

Supporting Information

## Data Availability

The research data supporting this publication can be accessed at https://doi.org/10.17630/76d51023‐456c‐4ad0‐9f9b‐262d88ca6f03.

## References

[1] R. G. Lawton , W. E. Barth , J. Am. Chem. Soc. 1971, 93, 1730–1745.

[2] W. E. Barth , R. G. Lawton , J. Am. Chem. Soc. 1966, 88, 380–381.

[3a] T. J. Seiders , K. K. Baldridge , G. H. Grube , J. S. Siegel , J. Am. Chem. Soc. 2001, 123, 517–525; 11456563 10.1021/ja0019981

[3b] Y.-T. Wu , J. S. Siegel , Chem. Rev. 2006, 106, 4843–4867; 17165677 10.1021/cr050554q

[3c] M. Juríček , N. L. Strutt , J. C. Barnes , A. M. Butterfield , E. J. Dale , K. K. Baldridge , J. F. Stoddart , J. S. Siegel , Nat. Chem. 2014, 6, 222–228. 24557137 10.1038/nchem.1842

[4a] K. K. Baldridge , J. S. Siegel , Theor. Chem. Acc. 1997, 97, 67–71;

[4b] F. J. Lovas , R. J. McMahon , J.-U. Grabow , M. Schnell , J. Mack , L. T. Scott , R. L. Kuczkowski , J. Am. Chem. Soc. 2005, 127, 4345–4349; 15783216 10.1021/ja0426239

[4c] L. M. Roch , L. Zoppi , J. S. Siegel , K. K. Baldridge , J. Phys. Chem. C 2017, 121, 1220–1234.

[5] B. A. Middleton , J. R. Partington , Nature 1938, 141, 516–517.

[6] R.-Q. Lu , Y.-Q. Zheng , Y.-N. Zhou , X.-Y. Yan , T. Lei , K. Shi , Y. Zhou , J. Pei , L. Zoppi , K. K. Baldridge , J. S. Siegel , X.-Y. Cao , J. Mater. Chem. A 2014, 2, 20515–20519.

[7] J. H. Kim , J. H. Yun , J. Y. Lee , Adv. Opt. Mater. 2018, 6, 1800255.

[8] A. Mishra , M. Ulaganathan , E. Edison , P. Borah , A. Mishra , S. Sreejith , S. Madhavi , M. C. Stuparu , ACS Macro Lett. 2017, 6, 1212–1216. 35650797 10.1021/acsmacrolett.7b00746

[9] Z. Xing , M.-W. An , Z.-C. Chen , M. Hu , X. Huang , L.-L. Deng , Q. Zhang , X. Guo , S.-Y. Xie , S. Yang , J. Am. Chem. Soc. 2022, 144, 13839–13850. 35862295 10.1021/jacs.2c05235

[10] X. Fu , Y. Zhen , Z. Ni , Y. Li , H. Dong , J. S. Siegel , W. Hu , Angew. Chem. Int. Ed. 2020, 59, 14024–14028. 10.1002/anie.202003179 32396268

[11] X. Gu , X. Zhang , H. Ma , S. Jia , P. Zhang , Y. Zhao , Q. Liu , J. Wang , X. Zheng , J. W. Y. Lam , D. Ding , B. Z. Tang , Adv. Mater. 2018, 30, 1801065. 10.1002/adma.201801065 29766581

[12a] J. F. Verdieck, W. A. Jankowski, in *Molecular Luminescence: An International Conference* (Ed.: E. C. Lim), Chicago, **1969**, pp. 829–836;

[12b] Y.-T. Wu , D. Bandera , R. Maag , A. Linden , K. K. Baldridge , J. S. Siegel , J. Am. Chem. Soc. 2008, 130, 10729–10739; 18642812 10.1021/ja802334n

[12c] L. Zoppi , L. Martin-Samos , K. K. Baldridge , J. Am. Chem. Soc. 2011, 133, 14002–14009; 21793582 10.1021/ja2040688

[12d] A. M. Rice , W. B. Fellows , E. A. Dolgopolova , A. B. Greytak , A. K. Vannucci , M. D. Smith , S. G. Karakalos , J. A. Krause , S. M. Avdoshenko , A. A. Popov , N. B. Shustova , Angew. Chem. Int. Ed. 2017, 56, 4525–4529; 10.1002/anie.201612199 PMC5396291 28332256

[12e] P. Bachawala , T. Ratterman , N. Kaval , J. Mack , Tetrahedron 2017, 73, 3831–3837;

[12f] C. S. Jones , E. Elliott , J. S. Siegel , Synlett 2004, 187–191;

[12g] J. Mack , P. Vogel , D. Jones , N. Kaval , A. Sutton , Org. Biomol. Chem. 2007, 5, 2448–2452; 17637965 10.1039/b705621d

[12h] G. H. Grube , E. L. Elliott , R. J. Steffens , C. S. Jones , K. K. Baldridge , J. S. Siegel , Org. Lett. 2003, 5, 713–716; 12605497 10.1021/ol027565f

[12i] T. J. Seiders , E. L. Elliott , G. H. Grube , J. S. Siegel , J. Am. Chem. Soc. 1999, 121, 7804–7813.

[13a] F. B. Bramwell , J. Gendell , J. Chem. Phys. 1970, 52, 5656–5661;

[13b] J. Janata , J. Gendell , C.-Y. Ling , W. E. Barth , L. Backes , H. B. Mark , R. G. Lawton , J. Am. Chem. Soc. 1967, 89, 3056–3058.

[14a] M. Kataoka , T. Nakajima , Tetrahedron 1986, 42, 6437–6442;

[14b] J. C. Hanson , C. E. Nordman , Acta Crystallogr. Sect. B 1976, 32, 1147–1153;

[14c] M. Randic , N. Trinajstic , J. Am. Chem. Soc. 1984, 106, 4428–4434.

[15a] L. T. Scott , M. M. Hashemi , D. T. Meyer , H. B. Warren , J. Am. Chem. Soc. 1991, 113, 7082–7084;

[15b] L. T. Scott , P.-C. Cheng , M. M. Hashemi , M. S. Bratcher , D. T. Meyer , H. B. Warren , J. Am. Chem. Soc. 1997, 119, 10963–10968.

[16] S. M. Argentine , A. H. Francis , C. C. Chen , C. M. Lieber , J. S. Siegel , J. Phys. Chem. 1994, 98, 7350–7354.

[17] S. Nakatsuka , N. Yasuda , T. Hatakeyama , J. Am. Chem. Soc. 2018, 140, 13562–13565. 30251839 10.1021/jacs.8b08197

[18] A. M. Butterfield , B. Gilomen , J. S. Siegel , Org. Process Res. Dev. 2012, 16, 664–676.

[19a] M. A. Baldo , D. F. O′Brien , Y. You , A. Shoustikov , S. Sibley , M. E. Thompson , S. R. Forrest , Nature 1998, 395, 151–154;

[19b] B. Minaev , G. Baryshnikov , H. Agren , Phys. Chem. Chem. Phys. 2014, 16, 1719–1758; 24346346 10.1039/c3cp53806k

[19c] C. Ulbricht , B. Beyer , C. Friebe , A. Winter , U. S. Schubert , Adv. Mater. 2009, 21, 4418–4441.

[20] W. Zhao , Z. He , B. Z. Tang , Nat. Rev. Mater. 2020, 5, 869–885.

[21a] T. Wang , X. Su , X. Zhang , W. Huang , L. Huang , X. Zhang , X. Sun , Y. Luo , G. Zhang , J. Mater. Chem. C 2019, 7, 9917–9925;

[21b] Z. Mao , Z. Yang , C. Xu , Z. Xie , L. Jiang , F. L. Gu , J. Zhao , Y. Zhang , M. P. Aldred , Z. Chi , Chem. Sci. 2019, 10, 7352–7357. 31489156 10.1039/c9sc02282a PMC6713867

[22a] X. Sun , B. Zhang , X. Li , C. O. Trindle , G. Zhang , J. Phys. Chem. A 2016, 120, 5791–5797; 27319778 10.1021/acs.jpca.6b03867

[22b] E. Hamzehpoor , C. Ruchlin , Y. Tao , C.-H. Liu , H. M. Titi , D. F. Perepichka , Nat. Chem. 2023, 15, 83–90; 36302870 10.1038/s41557-022-01070-4

[22c] O. Bolton , K. Lee , H.-J. Kim , K. Y. Lin , J. Kim , Nat. Chem. 2011, 3, 205–210. 21336325 10.1038/nchem.984

[23a] Z. Yang , Z. Mao , X. Zhang , D. Ou , Y. Mu , Y. Zhang , C. Zhao , S. Liu , Z. Chi , J. Xu , Y.-C. Wu , P.-Y. Lu , A. Lien , M. R. Bryce , Angew. Chem. Int. Ed. 2016, 55, 2181–2185; 10.1002/anie.201509224 PMC5064736 26836346

[23b] W. Z. Yuan , X. Y. Shen , H. Zhao , J. W. Lam , L. Tang , P. Lu , C. Wang , Y. Liu , Z. Wang , Q. Zheng , J. Phys. Chem. C 2010, 114, 6090–6099;

[23c] T. Wang , A. K. Gupta , S. Wu , A. M. Slawin , E. Zysman-Colman , J. Am. Chem. Soc. 2023, 145, 1945–1954. 36638828 10.1021/jacs.2c12320 PMC9880999

[24] T. Ono , A. Taema , A. Goto , Y. Hisaeda , Chem. Eur. J. 2018, 24, 17487–17496. 30295356 10.1002/chem.201804349

[25a] A. Cheng , Y. Jiang , H. Su , B. Zhang , J. Jiang , T. Wang , Y. Luo , G. Zhang , Angew. Chem. Int. Ed. 2022, 61, e202206366; 10.1002/anie.202206366 35670291

[25b] X. Zhang , J. Liu , B. Chen , X. He , X. Li , P. Wei , P. F. Gao , G. Zhang , J. W. Lam , B. Z. Tang , Matter 2022, 5, 3499–3512;

[25c] Y. Lei , W. Dai , J. Guan , S. Guo , F. Ren , Y. Zhou , J. Shi , B. Tong , Z. Cai , J. Zheng , Y. Dong , Angew. Chem. Int. Ed. 2020, 59, 16054–16060. 10.1002/anie.202003585 32500576

[26] M. Kasha , Discuss. Faraday Soc. 1950, 9, 14–19.

[27a] Y.-H. Wu , H. Xiao , B. Chen , R. G. Weiss , Y.-Z. Chen , C.-H. Tung , L.-Z. Wu , Angew. Chem. Int. Ed. 2020, 59, 10173–10178; 10.1002/anie.202000608 32012424

[27b] Z. He , W. Zhao , J. W. Y. Lam , Q. Peng , H. Ma , G. Liang , Z. Shuai , B. Z. Tang , Nat. Commun. 2017, 8, 416; 28871160 10.1038/s41467-017-00362-5 PMC5583377

[27c] T. Wang , Z. Hu , X. Nie , L. Huang , M. Hui , X. Sun , G. Zhang , Nat. Commun. 2021, 12, 1364; 33649318 10.1038/s41467-021-21676-5 PMC7921125

[27d] N. A. Kukhta , M. R. Bryce , Mater. Horiz. 2021, 8, 33–55; 34821289 10.1039/d0mh01316a

[27e] J. Yang , M. Fang , Z. Li , Acc. Mater. Res. 2021, 2, 644–654;

[27f] J. Li , X. Li , G. Wang , X. Wang , M. Wu , J. Liu , K. Zhang , Nat. Commun. 2023, 14, 1987; 37031245 10.1038/s41467-023-37662-y PMC10082826

[27g] T. Wang , Z. Tang , D. Xu , W. Sun , Y. Deng , Q. Wang , X. Zhang , P. Su , G. Zhang , Mater. Chem. Front. 2018, 2, 559–565.

[28] T. Wang , J. De , S. Wu , A. K. Gupta , E. Zysman-Colman , Angew. Chem. Int. Ed. 2022, 61, e202206681. 10.1002/anie.202206681 PMC9545188 35684990

[29] R. Huang , J. S. Ward , N. A. Kukhta , J. Avó , J. Gibson , T. Penfold , J. C. Lima , A. S. Batsanov , M. N. Berberan-Santos , M. R. Bryce , F. B. Dias , J. Mater. Chem. C 2018, 6, 9238–9247.

[30] T. Wang , A. K. Gupta , D. B. Cordes , A. M. Z. Slawin , E. Zysman-Colman , Adv. Opt. Mater. 2023, 11, 2300114.

[31] I. V. Kuvychko , S. N. Spisak , Y.-S. Chen , A. A. Popov , M. A. Petrukhina , S. H. Strauss , O. V. Boltalina , Angew. Chem. Int. Ed. 2012, 51, 4939–4942. 10.1002/anie.201200178 22492671

[32a] D. Eisenberg , A. S. Filatov , E. A. Jackson , M. Rabinovitz , M. A. Petrukhina , L. T. Scott , R. Shenhar , J. Org. Chem. 2008, 73, 6073–6078; 18505292 10.1021/jo800359z

[32b] A. S. Filatov , A. V. Zabula , S. N. Spisak , A. Y. Rogachev , M. A. Petrukhina , Angew. Chem. Int. Ed. 2014, 53, 140–145. 10.1002/anie.201308090 24375738

[33] S. Hirata , M. Head-Gordon , Chem. Phys. Lett. 1999, 314, 291–299.

[34] Y. Zhao , D. G. Truhlar , Theor. Chem. Acc. 2008, 120, 215–241.

[35] W. Humphrey , A. Dalke , K. Schulten , J. Mol. Graphics 1996, 14, 33–38. 10.1016/0263-7855(96)00018-5 8744570

[36] G. Rouillé , C. Jäger , M. Steglich , F. Huisken , T. Henning , G. Theumer , I. Bauer , H.-J. Knölker , ChemPhysChem 2008, 9, 2085–2091. 18798213 10.1002/cphc.200800387

[37a] P. L. dos Santos , J. S. Ward , A. S. Batsanov , M. R. Bryce , A. P. Monkman , J. Phys. Chem. C 2017, 121, 16462–16469;

[37b] S. Izumi , H. F. Higginbotham , A. Nyga , P. Stachelek , N. Tohnai , P. d Silva , P. Data , Y. Takeda , S. Minakata , J. Am. Chem. Soc. 2020, 142, 1482–1491. 31895980 10.1021/jacs.9b11578

[38a] W. Li , Y. Pan , R. Xiao , Q. Peng , S. Zhang , D. Ma , F. Li , F. Shen , Y. Wang , B. Yang , Y. Ma , Adv. Funct. Mater. 2014, 24, 1609–1614;

[38b] W. Li , D. Liu , F. Shen , D. Ma , Z. Wang , T. Feng , Y. Xu , B. Yang , Y. Ma , Adv. Funct. Mater. 2012, 22, 2797–2803.

[39] D. Zhang , M. Cai , Z. Bin , Y. Zhang , D. Zhang , L. Duan , Chem. Sci. 2016, 7, 3355–3363. 29997829 10.1039/c5sc04755b PMC6006951

[40a] D. Lee , O. Bolton , B. C. Kim , J. H. Youk , S. Takayama , J. Kim , J. Am. Chem. Soc. 2013, 135, 6325–6329; 23521108 10.1021/ja401769g

[40b] W. Qin , J. Ma , Y. Zhou , Q. Hu , Y. Zhou , G. Liang , Chem. Eng. J. 2020, 400, 125934;

[40c] J.-X. Wang , L.-Y. Peng , Z.-F. Liu , X. Zhu , L.-Y. Niu , G. Cui , Q.-Z. Yang , J. Phys. Chem. Lett. 2022, 13, 1985–1990. 35188776 10.1021/acs.jpclett.2c00168 PMC8900125

[41a] R. Kabe , N. Notsuka , K. Yoshida , C. Adachi , Adv. Mater. 2016, 28, 655–660; 26599764 10.1002/adma.201504321

[41b] S. Tan , K. Jinnai , R. Kabe , C. Adachi , Adv. Mater. 2021, 33, 2008844; 10.1002/adma.202008844 33945182

[41c] G. Xie , J. Wang , X. Xue , H. Li , N. Guo , H. Li , D. Wang , M. Li , W. Huang , R. Chen , Y. Tao , Appl. Phys. Rev. 2022, 9, 031410.

