# Supplementary material for: Room‐Temperature Multiple Phosphorescence from Functionalized Corannulenes: Temperature Sensing and Afterglow Organic Light‐Emitting Diode
Source: Angew Chem Int Ed Engl. 2023 Sep 15;62(43):e202309718. doi: 10.1002/anie.202309718 (PMC10953377; doi:10.1002/anie.202309718)
Supplement: Supplementary file 1 — Supporting Information [file ANIE-62-0-s001.pdf]

Angewandte Chemie
Eine Zeitschrift der Gesellschaft Deutscher Chemiker

Supporting Information

Room-Temperature Multiple Phosphorescence from Functionalized Corannulenes: Temperature Sensing and Afterglow Organic Light-Emitting Diode

*C. Si, T. Wang, A. K. Gupta, D. B. Cordes, A. M. Z. Slawin, J. S. Siegel, E. Zysman-Colman**

Table of Contents

General Methods S3

Literature Study S8

Experimental Section S9

X-Ray structural analysis S38

Theoretical calculation S42

Electrochemistry S49

Photophysical characterization S50

References S63

General Methods

General Synthetic Procedures

The following compounds were synthesised according to the literature: 9,9-dimethylacridan,[1] tetrabromocorannulene (**TBrCor**).[2] All commercially available chemicals and reagent grade solvents were used as received. Air-sensitive reactions were performed under a nitrogen atmosphere using Schlenk techniques. Flash column chromatography was carried out using silica gel (Silia-P from Silicycle, 60 Å, 40-63 μm). Analytical thin-layer-chromatography (TLC) was performed with silica plates with aluminum backings (250 μm with F-254 indicator). TLC visualization was accomplished by 254/365 nm UV lamp. HPLC analysis was conducted on a Shimadzu Prominence Modular HPLC system. HPLC traces were performed using an ACE Excel 2 C18 analytical column. ^{1}H and ^{13}C NMR spectra were recorded on a Bruker Advance spectrometer (400 or 500 MHz for ^{1}H, 101 or 126 MHz for ^{13}C). The following abbreviations have been used for multiplicity assignments: "s" for singlet, "d" for doublet, "t" for triplet, "dd" for doublet of doublets, "dt" for doublet of triplets, "ddd" for doublet of doublet of doublets, "q" for quintet and "m" for multiplet. Deuterated chloroform ($CDCl_3$) and dimethyl sulfoxide-d_6 (DMSO-d_6) were used as the solvent of record. ^{1}H NMR and ^{13}C NMR spectra were referenced to the solvent peak. Melting points were measured using open-ended capillaries on an Electrothermal melting point apparatus IA9200 and are uncorrected. High-resolution mass spectrometry (HRMS) was performed by Xevo G2-XS QTof Quadrupole Time-of-Flight Mass Spectrometry at the University of St Andrews. Elemental analyses were performed by Mr. Stephen Boyer, London Metropolitan University.

Theoretical calculation.

All ground-state optimizations have been performed the M062X functional[3] with the 6-31G(d,p) basis set[4] in the gas phase, based on the density functional theory (DFT). Vibrational frequency calculations were performed to ensure that the optimized geometries represented the local minima. Excited-state calculations have been performed at time-dependent DFT (TD-DFT) using the same functional and basis set within the Tamm-Dancoff approximation (TDA)[5] as for ground-state geometry optimization. Spin-orbit coupling matrix elements (ξ) were calculated based on the optimized singlet excited state geometry. Spin-orbit coupling matrix elements between singlet and triplet excited states were calculated using the PySOC program.[6] Hole-electron and reduced density gradient (RDG)[7] analyses were conducted using the Multiwfn program,[8] and the corresponding molecular orbitals were visualized using VMD program.[9]

Electrochemistry measurements.

Cyclic Voltammetry (CV) and differential pulse voltammetry (DPV) analysis were performed on an Electrochemical Analyzer potentiostat model 620E from CH Instruments. Samples were prepared in dichloromethane (DCM) solutions, which were degassed by sparging with DCM-saturated nitrogen gas for 5 minutes prior to measurements. All measurements were performed using 0.1 M tetra-*n*-butylammonium hexafluorophosphate, $[^{n}Bu_4N]PF_6$, in DCM. An Ag/Ag^+ electrode was used as the reference electrode, a platinum electrode was used as the working electrode, and a platinum wire was used as the counter electrode. The redox potentials are reported relative to a saturated calomel electrode (SCE) with a ferrocene/ferrocenium (Fc/Fc^+) redox couple as the internal standard (0.46 V vs SCE).[10] The HOMO and LUMO energies were calculated using the relation $E_{HOMO/LUMO} = -(E_{ox}/E_{red} - E_{Fc/Fc+} + 4.8)$ eV,[11] where E_{ox} and E_{red} are anodic and cathodic peak potentials obtained from DPV, respectively.

Photophysical measurements

All samples were prepared in HPLC grade toluene (PhMe), dichloromethane (DCM) or acetonitrile (MeCN) with varying concentrations on the order of 10^{-5} or 10^{-6} M for absorption and emission study. Absorption spectra were recorded at RT using a Shimadzu UV-2600 double beam spectrophotometer. Molar absorptivity determination was verified by linear least-squares fit of values obtained from at least five independent solutions at varying concentrations with absorbance ranging from 1.44×10^{-3} to 3.00×10^{-1} M^{-1} cm^{-1} for **TCzPhCor**, 4.15×10^{-5} to 4.89×10^{-2} M^{-1} cm^{-1} for **TDMACPhCor**, 5.99×10^{-5} to 1.91×10^{-1} M^{-1} cm^{-1} for **TPXZPhCor**.

Degassed solutions were prepared via three freeze-pump-thaw cycles prior to emission analysis using an in-house adapted fluorescence cuvette, itself purchased from Starna. Steady-state emission and time-resolved emission spectra were recorded at 298 K using an Edinburgh Instruments F980 fluorimeter. All the samples for the steady-state measurements were excited at 340 nm using a Xenon lamp, while the samples for the time-resolved measurements were excited at 379 nm using a pico-second laser (PicoQuant, LDH-D-C-375) driven by a laser driver (PDL 800-D). The short-time range (200 ns-20 μs) of PL decays were measured using time-correlated single photon counting (TCSPC) mode. The long-time range (10 ms-4 s) of PL decays were measured using multi-channel scaling (MCS) mode. In MCS mode, the picosecond laser was triggered by a delay generator (Stanford Research

Systems, DG645) in the burst mode. The burst mode increased the excitation pulse duration to increase the excitation power to reduce the measurement time.

The singlet-triplet splitting energy, ΔE_{ST}, was estimated by recording the prompt fluorescence and the delayed phosphorescence spectra at 77 K. 77 K glass samples were prepared by transferring toluene solution into NMR tubes and the NMR tubes were cooled down inside a suprasil nitrogen Dewar flask by liquid nitrogen. Prompt fluorescence spectra (1-100 ns) in toluene at 77 K were measured by time-resolve PL spectroscopy using a 5 mW EPL-375 picosecond pulsed laser as the excitation source. Phosphorescence spectra (1-10 ms) in toluene at 77 K were measured using time-gated PL spectroscopy, where the samples were excited by a 5 W microsecond flash lamp (l_{exc} = 320 nm). The energy values of the lowest singlet and triplet states were determined from the onset of fluorescence and phosphorescence spectra at 77 K, respectively. Phosphorescence spectra (30-200 ms) for all doped films were collected with a 5 W microsecond flash lamp by the MCS mode using an Edinburgh Instruments FS5 (l_{exc} = 320 nm).

Photoluminescence quantum yields for solutions were excited at 360 nm for **TCzPhCor**, **TDMACPhCor** and **TPXZPhCor** and determined using the optically dilute method.[12,13] The Beer-Lambert law was found to be linear at the concentrations of the solutions. For each sample, linearity between absorption and emission intensity was verified through linear regression analysis and additional measurements were acquired until the Pearson regression factor (R^2) for the linear fit of the data set surpassed 0.9. Individual relative quantum yield values were calculated for each solution and the values reported represent the gradient value. The equation $\Phi_s = \Phi_r(A_r/A_s)(I_s/I_r)(n_s/n_r)^2$ was used to calculate the relative quantum yield of each of the sample, where Φ_r is the absolute quantum yield of the reference, *n* is the refractive index of the solvent, *A* is the absorbance at the excitation wavelength, and *I* is the integrated area under the corrected emission curve. The subscripts *s* and *r* refer to the sample and reference, respectively. A solution of quinine sulfate (Φ_r = 54.6% in 1 *N* H_2SO_4)[14] was used as the external reference. An integrating sphere (SC-30 module on FS5 fluorimeter) Hamamatsu C9920-02 was employed for Φ_{PL} measurements for thin film samples. A xenon lamp coupled to a monochromator enabled selective excitation, chosen here to be 340 nm. The output was then fed into the integrating sphere via a fiber, exciting the sample. PL spectra were collected with a multimode fiber and detected with a backthinned CCD. The doped thin films were prepared by spin-coating a 1 wt% chloroform solution of emitter in PMMA while the PL properties of the emitters in a larger range concentration from 1 wt% to 30 wt% in mCP were investigated. The quantum yields of the films were measured in air and N_2 atmosphere by purging the integrating sphere with flowing N_2 gas.

X-ray crystallography

X-ray diffraction data for both compounds were collected at either 173 K or 125 K using a Rigaku MM-007HF High Brilliance RA generator/confocal optics with XtaLAB P200 diffractometer [Cu Kα radiation (λ = 1.54187 Å)]. Intensity data were collected using ω steps accumulating area detector images spanning at least a hemisphere of reciprocal space. Data were collected using CrystalClear[15] and processed (including correction for Lorentz, polarization and absorption) using CrysAlisPro.[16] The structure was solved by dual space (SHELXT[17]) or direct (SIR2011[18]) methods and refined by full-matrix least-squares against F^2 (SHELXL-2018/3[19]). Non-hydrogen atoms were refined anisotropically, and hydrogen atoms were refined using a riding model. The structure of **TPXZPhCor** showed some void space (817 Å^3) containing electron density that did not make a chemically sensible model and the SQUEEZE[20] routine implemented in PLATON[21] was used to remove the contribution to the diffraction pattern of the unordered electron density in the void spaces. All calculations except SQUEEZE were performed using the Olex2[22] or CrystalStructure[23] interface. Selected crystallographic data are presented in Table **S1**. Deposition numbers 2279496-2279497 contains the supplementary crystallographic data for this paper. These data can be obtained free of charge from The Cambridge Crystallographic Data Centre via www.ccdc.cam.ac.uk/structures.

Table **S1**. Selected crystallographic data.

	**TPhCor**	**TPXZPhCor**
formula	$C_{46}H_{26}$	$C_{102.5}H_{66}N_4O_4$
fw	554.65	1417.67
crystal description	colourless prism	yellow plate
crystal size [mm^3]	0.15×0.03×0.02	0.15×0.08×0.02
temperature [K]	173	125
space group	$P\bar{1}$	$P\bar{1}$
a [Å]	8.35421(14)	14.2669(4)
b [Å]	11.06986(17)	19.3001(6)
c [Å]	16.1177(3)	31.2768(8)
α [°]	73.7393(14)	74.436(2)
β [°]	87.2915(14)	84.770(2)
γ [°]	89.8093(14)	69.627(3)
vol [Å]^3	1429.26(4)	7777.4(4)
Z	2	4
ρ (calc) [g/cm^3]	1.289	1.211
μ [mm^{-1}]	0.556	0.575
F(000)	580	2964
reflections collected	17317	93634
independent reflections (R_{int})	5660 (0.0163)	30718 (0.0860)
parameters, restraints	397, 0	1960, 621
GoF on F^2	1.068	1.058
R_1 [$I > 2\sigma(I)$]	0.0390	0.0920
wR_2 (all data)	0.1048	0.3217
largest diff. peak/hole [e/Å^3]	0.19, -0.18	0.62, -0.36

OLED Fabrication and Characterization

The OLED devices were fabricated in a bottom emitting architecture on indium-doped tin oxide (ITO, 12 mm × 12 mm × 1.1 mm, resistivity: 15 Ω sq^{-1}) substrates. A pre-patterned glass substrate coated with ITO were washed sequentially by ultrasonication in acetone, and isopropanol for 20 min and then exposed to oxygen plasma for 10 min to remove all the dust and organics on the ITO surface and to increase the work function of ITO anode for better hole injection from the anode to organic layer. The hole injection layer, poly(3,4-ethylenedioxythiophene):poly(styrenesulfonate) (PEDOT:PSS; Heraeus; Clevios P VP AI 4083; charge: 9001157883), was spin coated onto the ITO surface under 4000 rpm for 1 min, and baked at 130 °C for 15 min to remove the residual water, then the substrates were transferred into a nitrogen-filled glovebox. The emitting layer (15% emitter/ 85% mCP in $CHCl_3$) was spin-coated under 2000 rpm (30 nm), and then annealed at 60 °C in the nitrogen-filled glovebox. The electron transporting layer (2,2',2''-(1,3,5-benzinetriyl)-tris(1-phenyl-1-*H*-benzimidazole), TPBi, 50 nm), the electron injecting layer (lithium fluoride, LiF 1 nm), and the aluminum cathode (100 nm) were successively thermally evaporated in a vacuum chamber under <1 × 10^{-6} mbar. After the evaporation, the OLEDs were taken out from the evaporator and encapsulated inside the glovebox.The devices were taken out from the glovebox for current−voltage−luminance characteristics. The luminance-current-voltage characteristics were measured in an ambient environment using a Keithley 2400 source meter combined with a homemade photodiode connected to a Keithley 2000 multimeter for the voltage reading. The external quantum efficiency was calculated assuming Lambertian emission distribution. The electroluminescence spectra were recorded by an Andor DV420-BV CCD spectrometer.

Literature Study

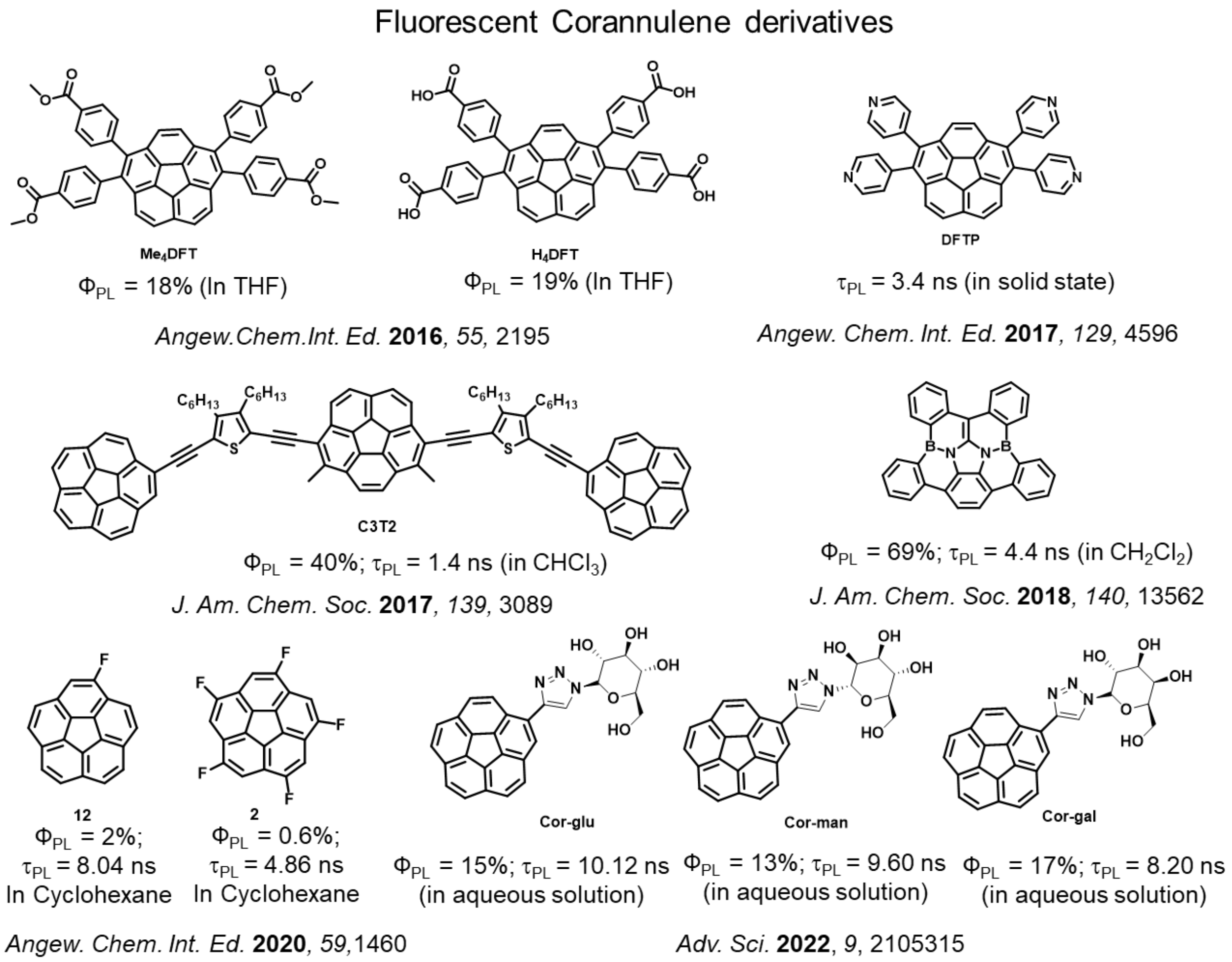

Figure **S1**. Reported fluorescent corannulene derivatives discussed in the manuscript.

Experimental Section

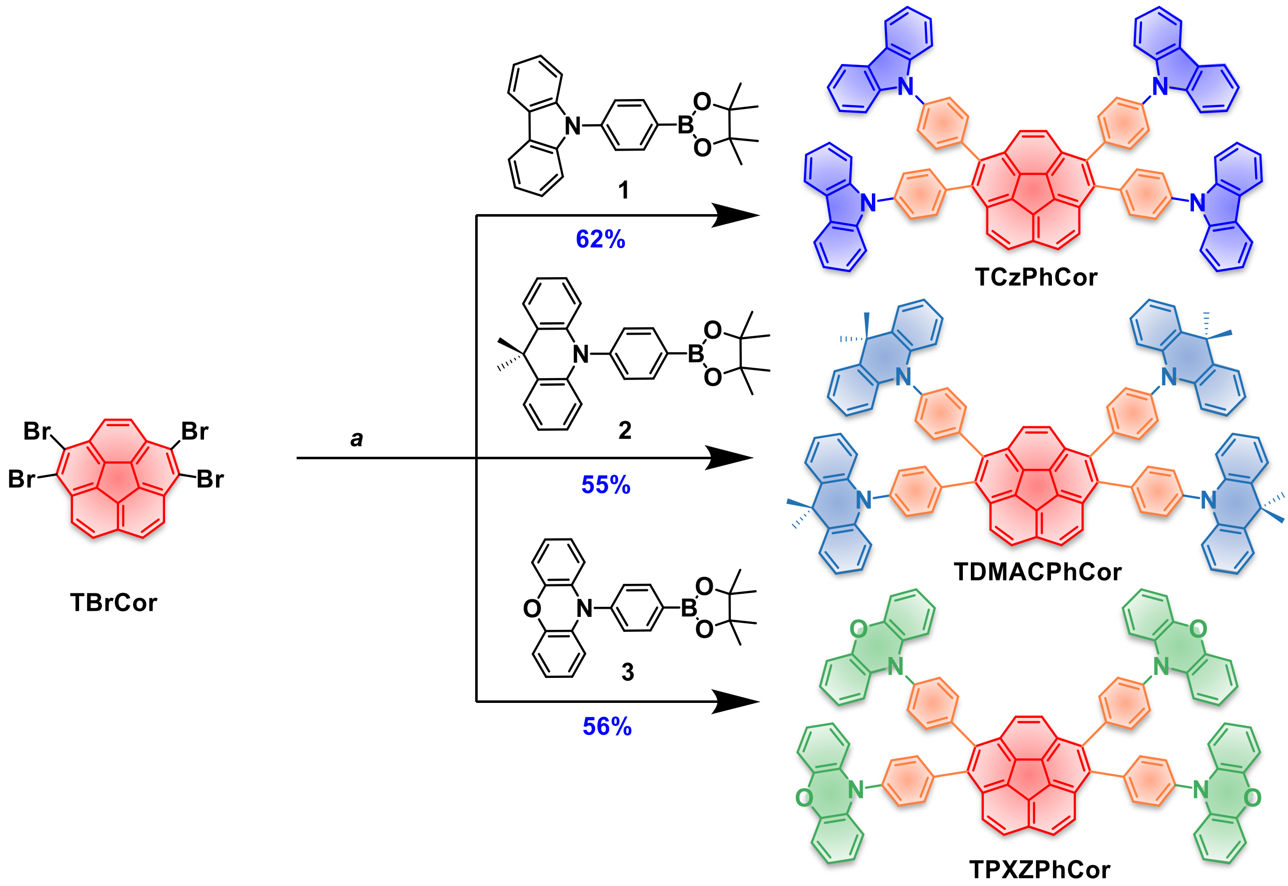

Scheme **S1**. Synthetic route for **TCzPhCor, TDMACPhCor** and **TPXZPhCor**. Reagents and conditions: [a]THF, $(K_2CO_3)_{aq}$, $Pd(PPh_3)_4$ (0.2 equiv.), 80 °C, 12 h.

Synthesis of 9-(4-bromophenyl)-9H-carbazole (1):

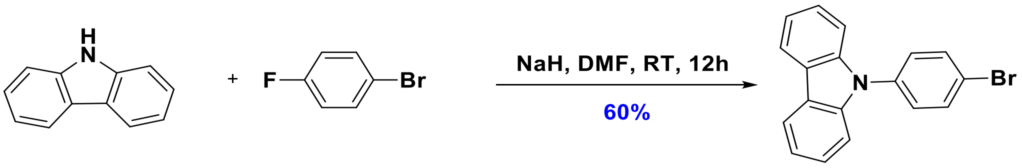

1

To a solution of carbazole (2.14 g, 12.8 mmol, 1.0 equiv.) in dry DMF (50 mL) at room temperature under a nitrogen atmosphere was added portion-wise sodium hydride (60% in mineral oil, 0.56 g, 14.1 mmol, 1.1 equiv.). The resulting suspension was stirred under nitrogen atmosphere for 30 minutes. Then a solution of 1-bromo-4-fluorobenzene (2.24 g, 12.8 mmol, 1.0 equiv.) in dry DMF (30 mL) was added to the stirred suspension. The resulting reaction mixture was allowed to stir for a further 12 h. The reaction mixture was then slowly added to ice water and then extracted with DCM (3 × 50 mL). The combined organic layers were dried with anhydrous sodium sulfate and concentrated under

reduced pressure. The crude product was purified by silica gel flash column chromatography using DCM: hexane= 1:10 as eluent to afford the desired compound as a white solid (yield = 2.74 g).

9-(4-bromophenyl)-9H-carbazole (1): Yield: 60%.**R_f**= 0.4 (Hexane)**. Mp** = 143-145 °C. (Lit. Mp:[24] 144-145 °C) **^{1}H NMR (500 MHz, $CDCl_3$):** δ = 8.19 (d, J = 7.8 Hz, 2H), 7.77 (d, J = 8.6 Hz, 2H), 7.51 – 7.46 (m, 3H), 7.45 – 7.41 (m, 3H), 7.35 (ddd, J = 8.0, 6.8, 1.2 Hz, 2H). ppm**. ^{13}C NMR (126 MHz, $CDCl_3$):** δ = 140.60, 136.80, 133.14, 128.74, 126.13, 123.50, 120.91, 120.45, 120.25, 109.59 ppm. Analytical data matches that previously reported.[24]

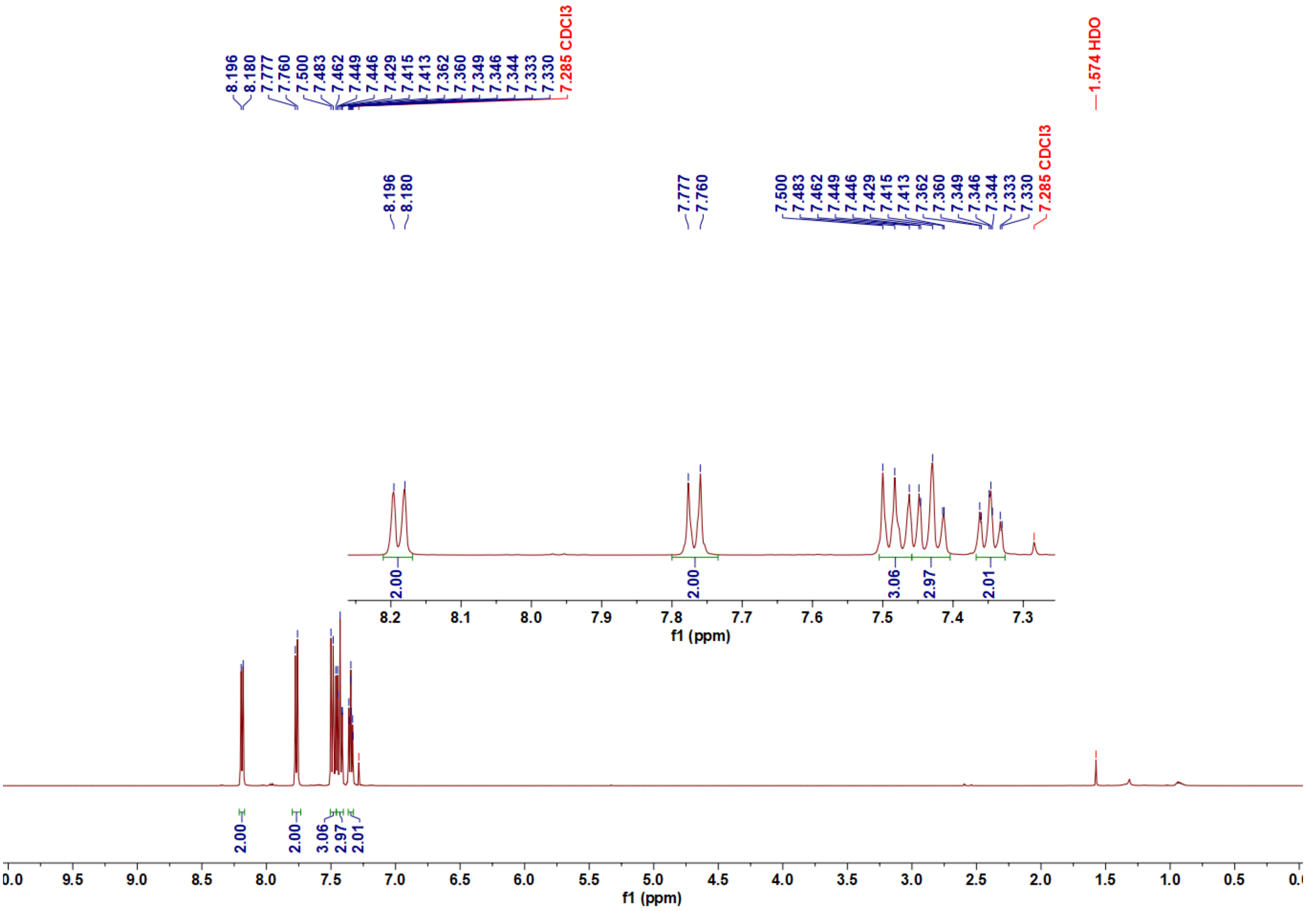

Figure **S2**. ^{1}H NMR spectra of **1** in $CDCl_3$.

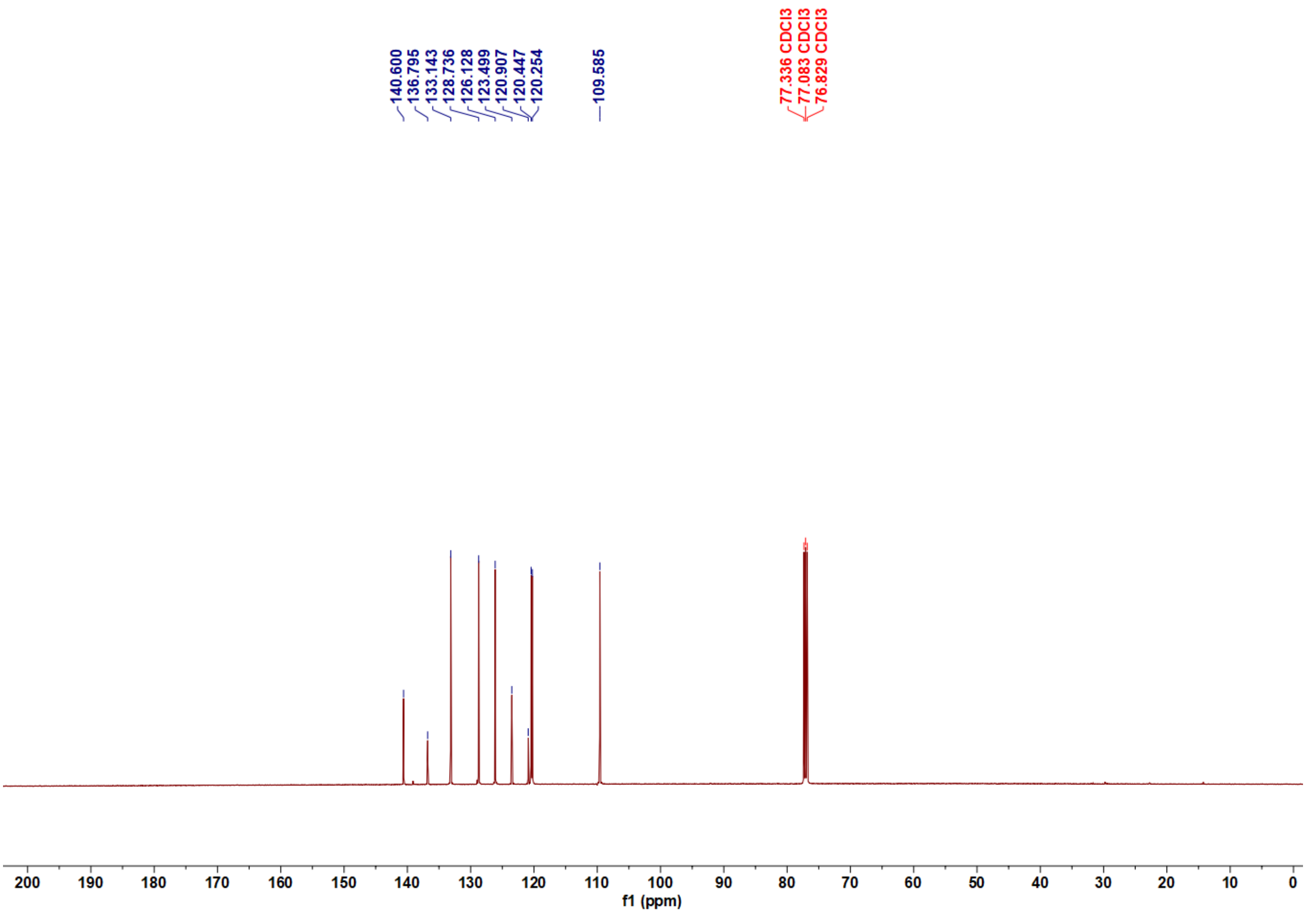

Figure **S3**. ^{13}C NMR spectra of **1** in $CDCl_3$.

Synthesis of 9-(4-(4,4,5,5-tetramethyl-1,3,2-dioxaborolan-2-yl) phenyl)-9H-carbazole (2):

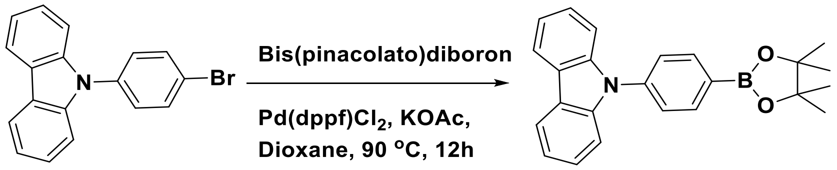

1 84% 2

A Schlenk flask was charged with **1** (1.29 g, 4.0 mmol, 1.0 equiv.), then bis(pinacolato)diboron (1.52 g, 6.0 mmol, 1.5 equiv.), Pd(dppf)Cl_2 (146 mg, 0.2 mmol, 0.05 equiv.), KOAc (1.18 g, 12.0 mmol, 3.0 equiv.) and dry 1,4-dioxane (40 mL) were added to the flask under nitrogen. The mixture was heated to 90 °C with stirring for about 12 h. Then the mixture was cooled to room temperature and extracted with ethyl acetate (3 × 50 mL). The combined organic layers were dried with anhydrous sodium sulfate and concentrated under reduced pressure. The crude product was purified by silica gel flash column chromatography using EA:hexane = 1:10 as eluent to give compound **2** as a white solid (yield =1.24 g).

9-(4-(4,4,5,5-tetramethyl-1,3,2-dioxaborolan-2-yl) phenyl)-9H-carbazole (2): R_f = 0.3 (10% EA/Hexane). **Yield:** 84%. **Mp** = 170-171 °C. (Lit. Mp:[25] 168 °C) **^{1}H NMR (500 MHz, $CDCl_3$):** δ = 8.17 (d, J = 7.8 Hz, 2H), 8.08 (d, J = 8.2 Hz, 2H), 7.63 (d, J = 8.2 Hz, 2H), 7.48 (dd, J = 8.2, 1.0 Hz, 2H), 7.46 – 7.41 (m, 2H), 7.32 (ddd, J = 8.0, 7.0, 1.2 Hz, 2H), 1.43 (s, 12H) ppm. **^{13}C NMR (126 MHz, $CDCl_3$):** δ = 140.57, 140.35, 136.39, 126.08, 125.98, 123.51, 120.32, 120.07, 109.85, 84.11, 24.95 ppm. Analytical data matches that previously reported.[25]

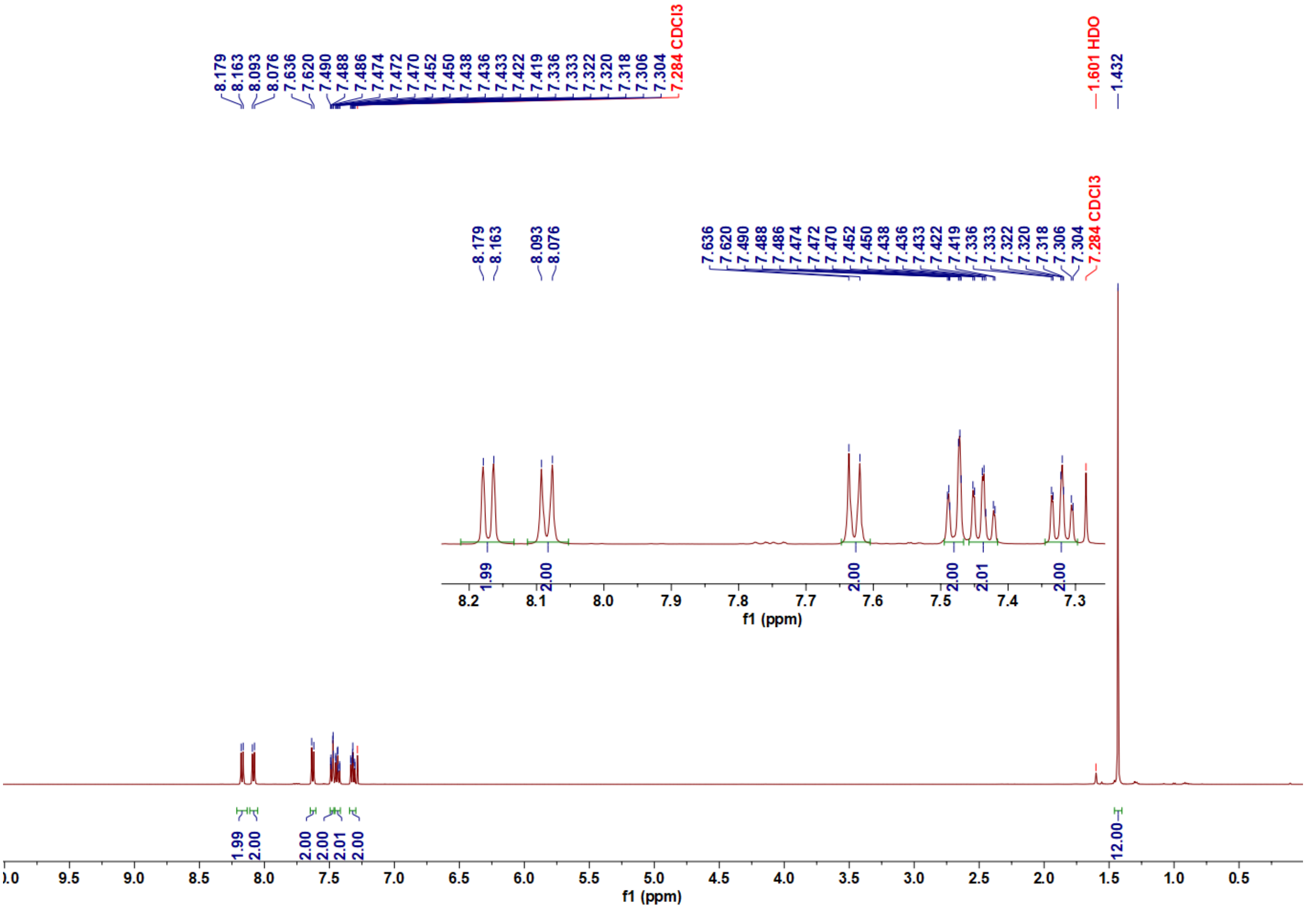

Figure **S4**. ^{1}H NMR spectra of **2** in $CDCl_3$.

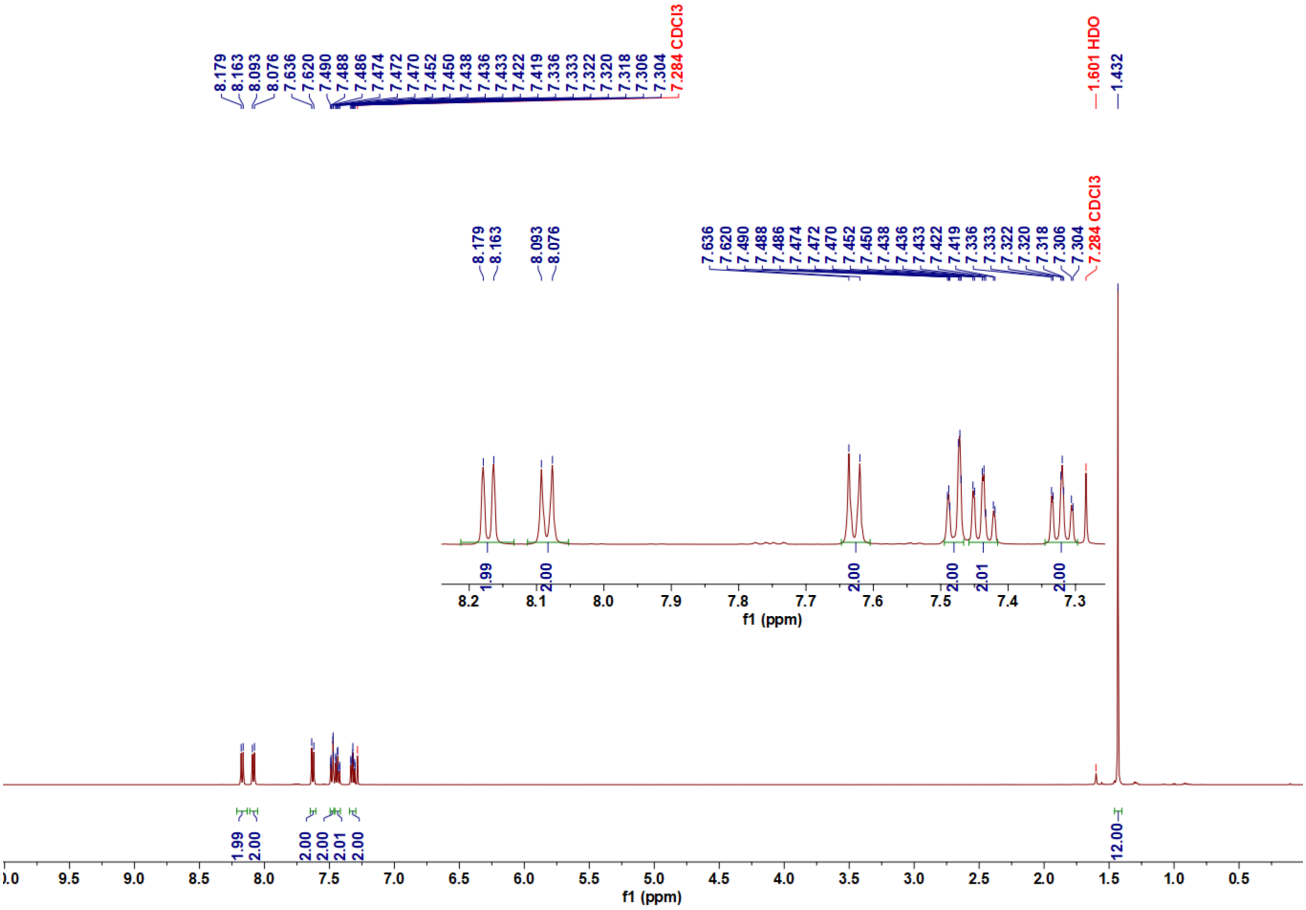

Figure **S5**. ^{13}C NMR spectra of **2** in $CDCl_3$.

Synthesis of 10-(4-bromophenyl)-9,9-dimethyl-9,10-dihydroacridine (3):

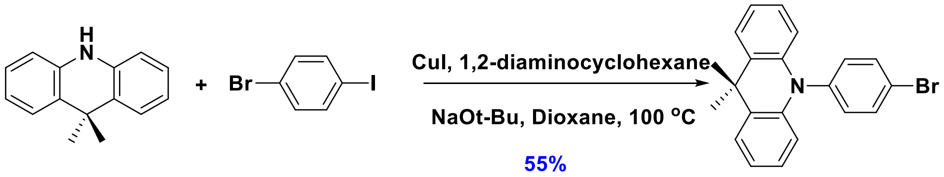

3

A mixture of 9,9-dimethylacridan (5.7 g, 27.25 mmol, 1.0 equiv.), 1-bromo-4-iodobenzene (8.5 g, 30.0 mmol, 1.1 equiv.), copper(I) iodide (0.11 g, 0.55 mmol, 0.02 equiv.), sodium *tert*-butoxide (5 g, 54 mmol, 2.0 equiv), and 1,2-diaminocyclohexane (0.32 g, 2.8 mmol, 0.1 equiv) in dry 1,4-dioxane (50 mL) was refluxed for 12 h. After cooling to room temperature, the reaction mixture was added into water, and then extracted with DCM. The combined organic layers were dried over anhydrous magnesium sulfate and concentrated under reduced pressure. The crude product was purified by column chromatography on silica gel (DCM: hexane/ = 1:9, v/v) to give **3** as a white solid (yield =5.46 g).

10-(4-bromophenyl)-9,9-dimethyl-9,10-dihydroacridine (3): Yield: 55%. **R_f** = 0.6 (10% DCM/Hexane)**. Mp=**180-181 °C. **^{1}H NMR (400 MHz, $CDCl_3$)** δ 7.83 – 7.74 (m, 2H), 7.52 – 7.44 (m, 2H), 7.28 – 7.22 (m, 2H), 7.05 – 6.92 (m, 4H), 6.31 – 6.24 (m, 2H), 1.71 (s, 6H) ppm. **^{13}C NMR (101 MHz, $CDCl_3$): δ** = 140.60, 140.30, 134.24, 133.24, 130.09, 126.43, 125.34, 122.11, 120.82, 113.90, 77.35, 77.04, 76.72, 35.98, 31.25 ppm. Analytical data matches that previously reported.[26]

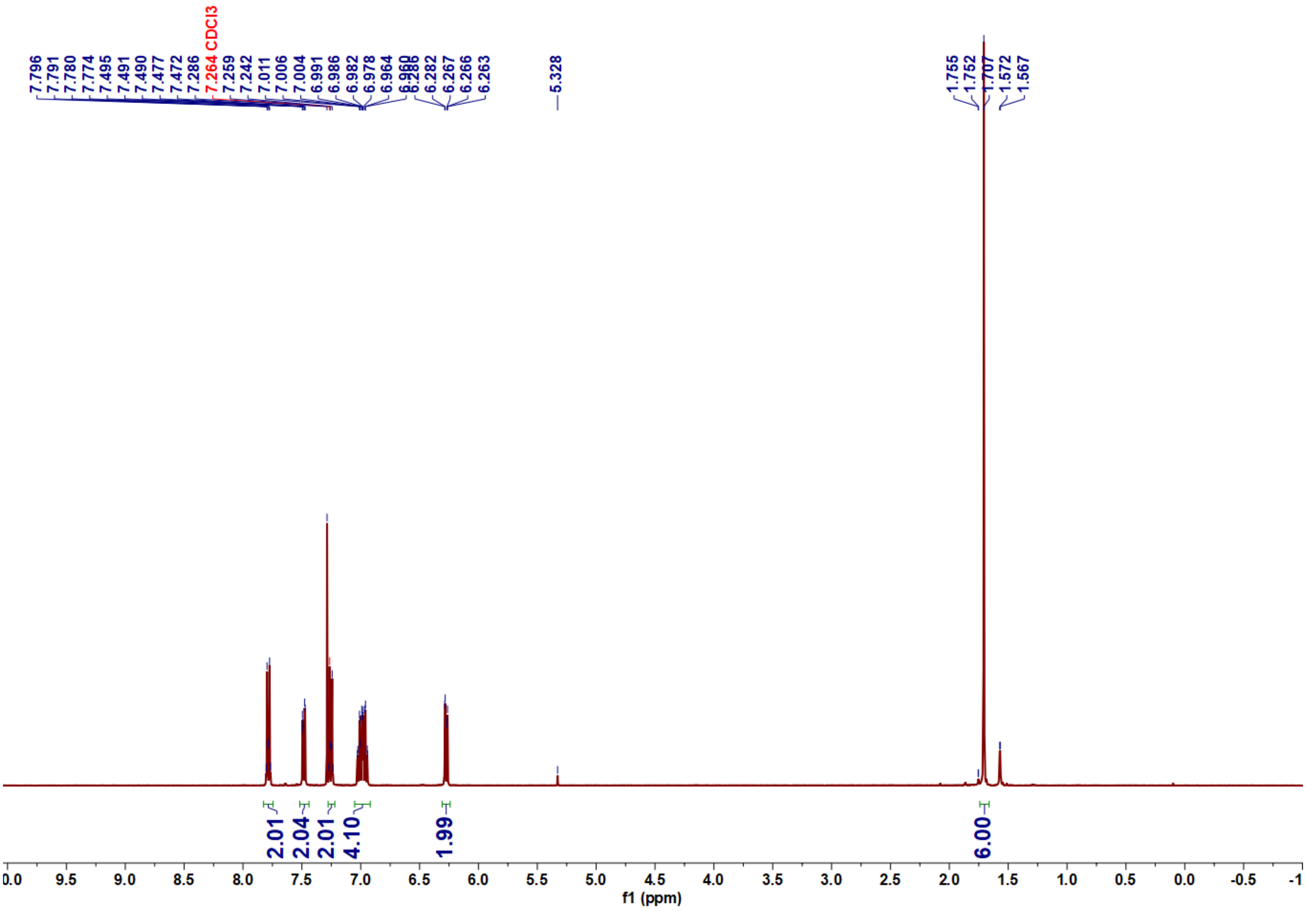

Figure **S6.** ^{1}H NMR spectra of **3** in $CDCl_3$.

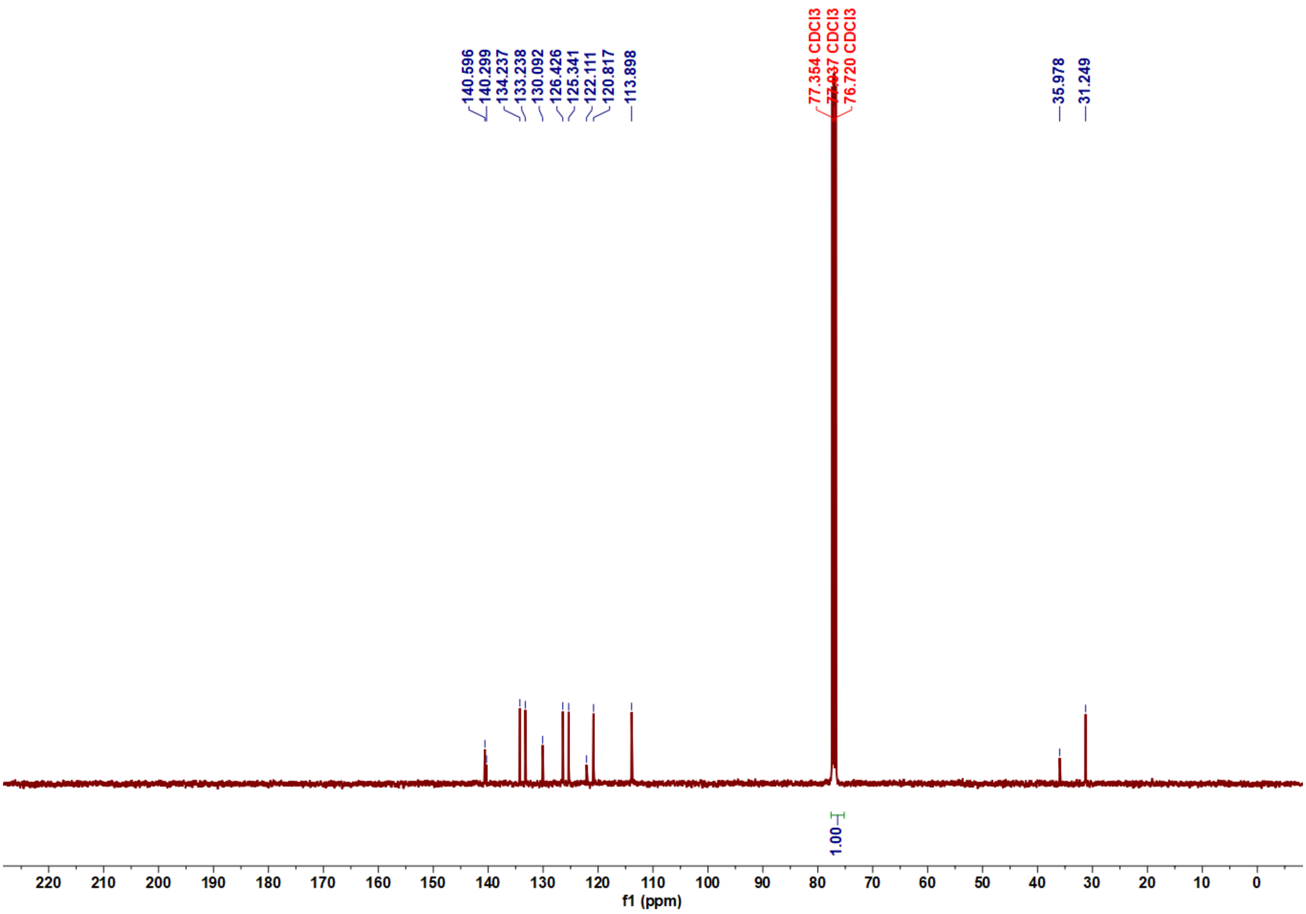

Figure **S7**. ^{13}C NMR spectra of **3** in $CDCl_3$.

Synthesis of 9,9-dimethyl-10-(4-(4,4,5,5-tetramethyl-1,3,2-dioxaborolan-2-yl)phenyl)-9,10-dihydroacridine (4):

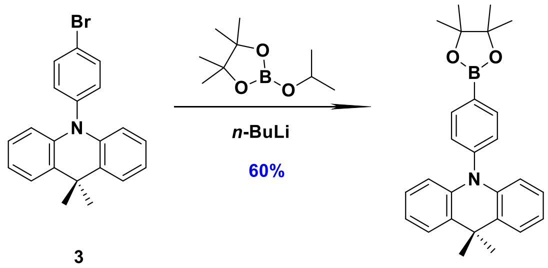

3

4

To a solution of **3** (5.0 g, 13.8 mmol, 1.0 equiv.) in dry THF (100 mL) was added dropwise *n*-BuLi (2.6 M, 6.0 mL, 16 mmol, 1.15 equiv.) at -78 °C. The mixture was stirred for 1 h at -78 °C. Then, 2-isopropoxy-4,4,5,5-tetramethyl-1,3,2-dioxaborolane (3.1 g, 16.5 mmol, 1.2 equiv.) was added dropwise to the mixture at -78 °C. The reaction mixture was allowed to warm to room temperature and stirred for a further 3 h. The resulting mixture was added into water, and then extracted with chloroform (3 × 25 mL). The combined organic layers were washed with water, dried over anhydrous magnesium

sulfate and concentrated under reduced pressure. The crude product was purified by column chromatography on silica gel (DCM: hexane/ =1: 4, v/v) to afford **4** as a white solid (yield =3.40 g).

9,9-dimethyl-10-(4-(4,4,5,5-tetramethyl-1,3,2-dioxaborolan-2-yl)phenyl)-9,10-dihydroacridine (4): R$_f$ = 0.4 (25% DCM/Hexane)**. Yield:** 60%. **Mp** = 169-171 °C. **^{1}H NMR (400 MHz, DMSO-*d*$_6$)** δ 8.02 –7.93 (m, 2H), 7.49 (dd, *J* = 7.6, 1.7 Hz, 2H), 7.40 – 7.35 (m, 2H), 6.99 – 6.87 (m, 4H), 6.13 (dd, *J* = 8.1, 1.4 Hz, 2H), 1.61 (s, 6H), 1.35 (s, 12H) ppm. . **^{13}C NMR (101 MHz, CDCl$_3$):** δ = 143.99, 140.72, 137.33, 130.66, 129.94, 126.34, 125.23, 120.53, 114.09, 84.13, 77.36, 77.04, 76.72, 35.98, 31.34, 24.96. Analytical data matches that previously reported.[26]

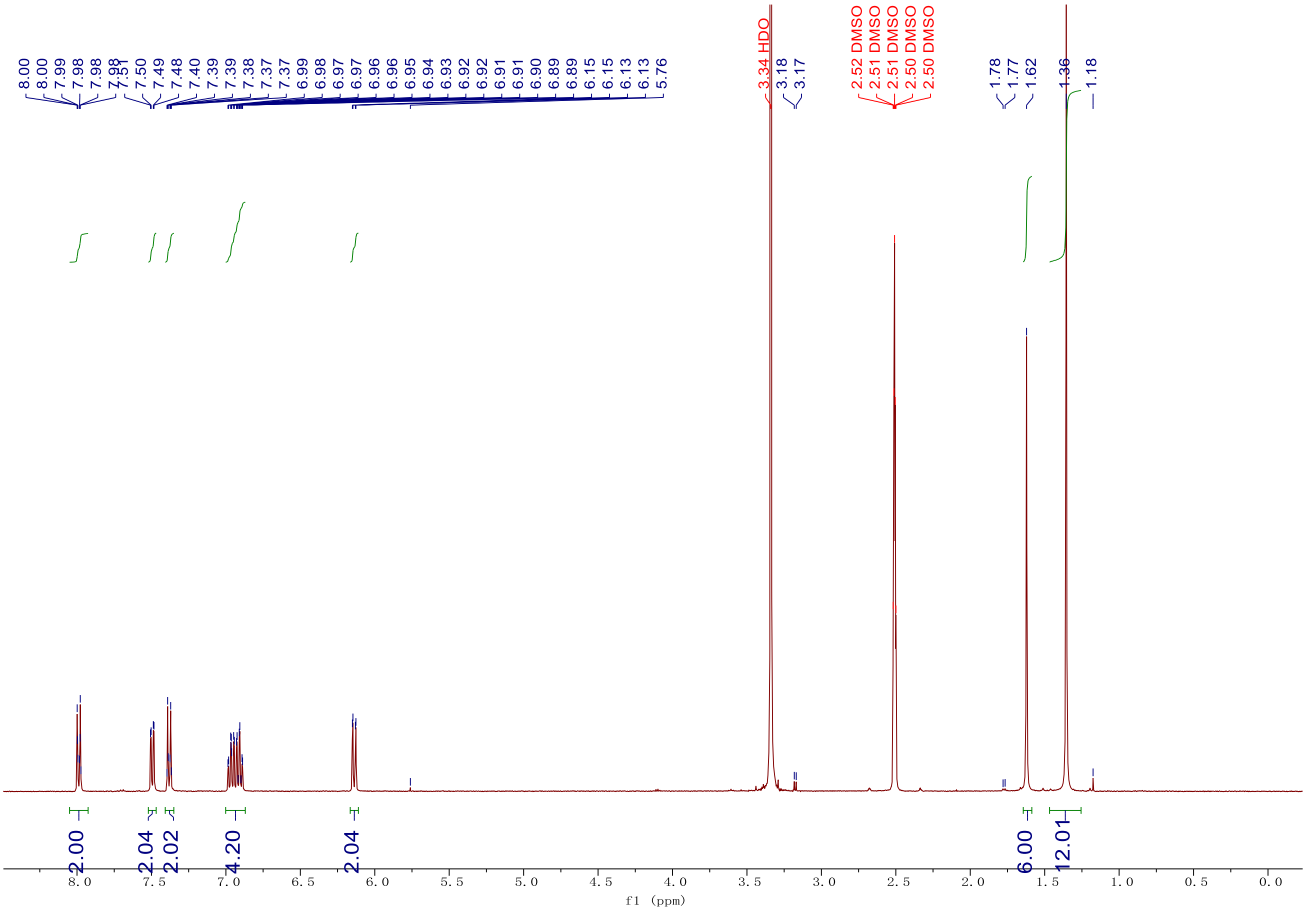

Figure **S8**. ^{1}H NMR spectra of **4** in DMSO-*d*$_6$.

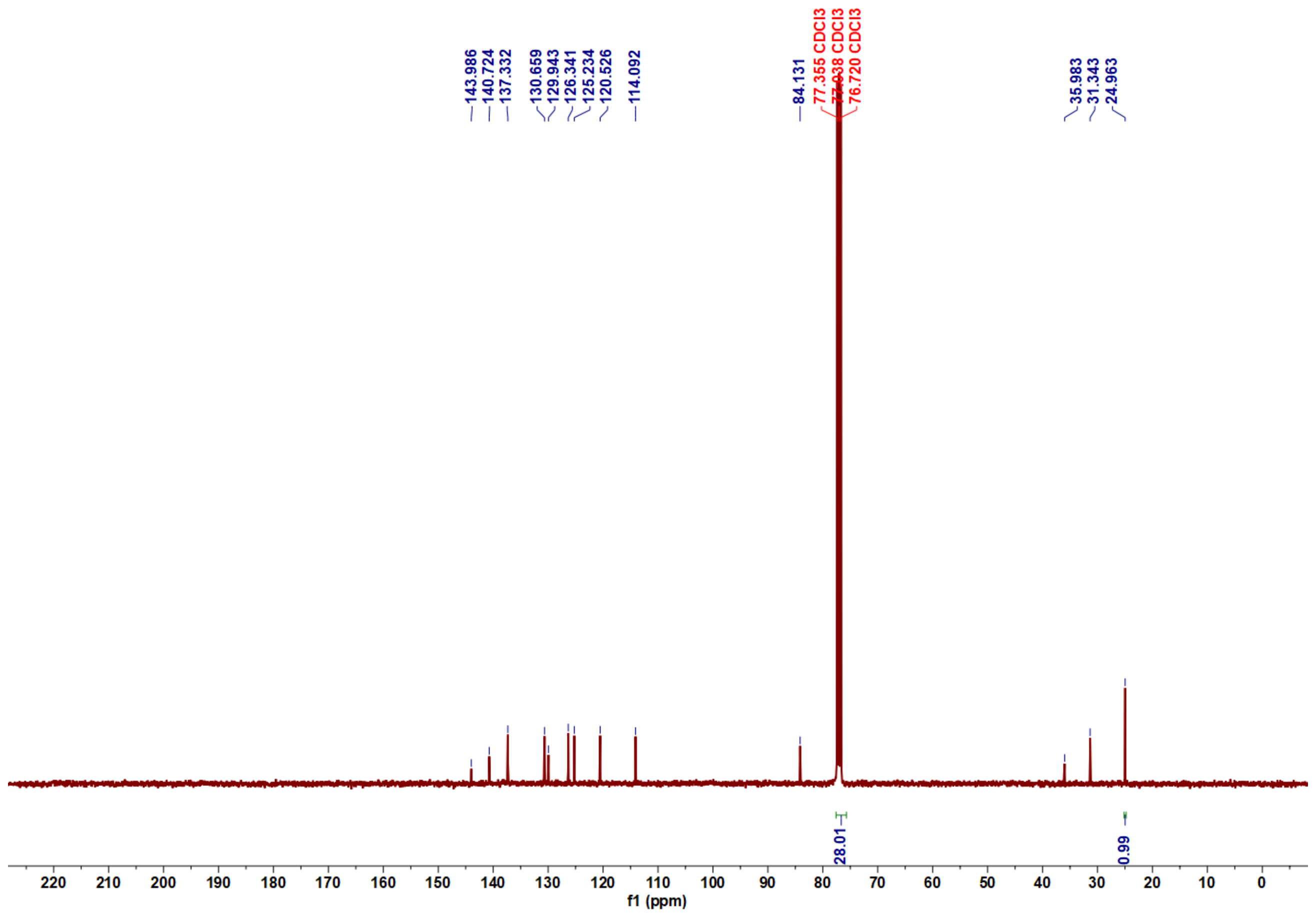

Figure **S9**. ^{13}C NMR spectra of **4** in $CDCl_3$.

Synthesis of 10-(4-bromophenyl)-10H-phenoxazine (5):

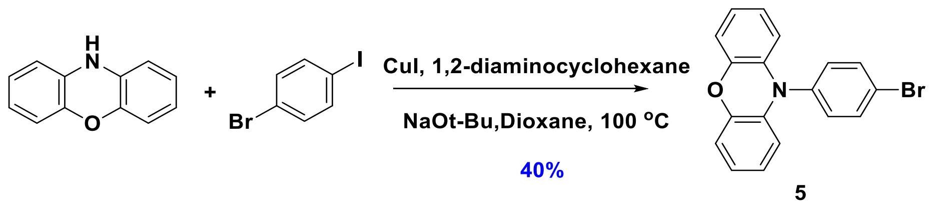

Compound **5** was synthesized according to the same procedure as described above for the synthesis of **3**, except that phenoxazine (5 g, 27 mmol) was used as the reactant instead of 9,9-dimethylacridan, yielding a white solid (yield =3.70 g).

10-(4-bromophenyl)-10H-phenoxazine (5): R$_f$=0.6 (10% DCM/Hexane)**. Yield:** 40%. **Mp** =180-183 °C. (Lit. Mp:[27] 184-185 °C) **^{1}H NMR (500 MHz, $CDCl_3$)**: δ 7.89–7.82 (m, 2H), 7.43–7.37 (m, 2H), 6.77–6.71 (m, 2H), 6.70 – 6.63 (m, 4H), 5.91–5.83 (m, 2H) ppm. **^{13}C NMR (101 MHz, $CDCl_3$):** δ = 143.91, 134.45, 133.99, 133.73, 132.76, 123.29, 122.38, 121.62, 115.58, 113.18, 77.36, 77.04, 76.73 ppm. Analytical data matches that previously reported.[26]

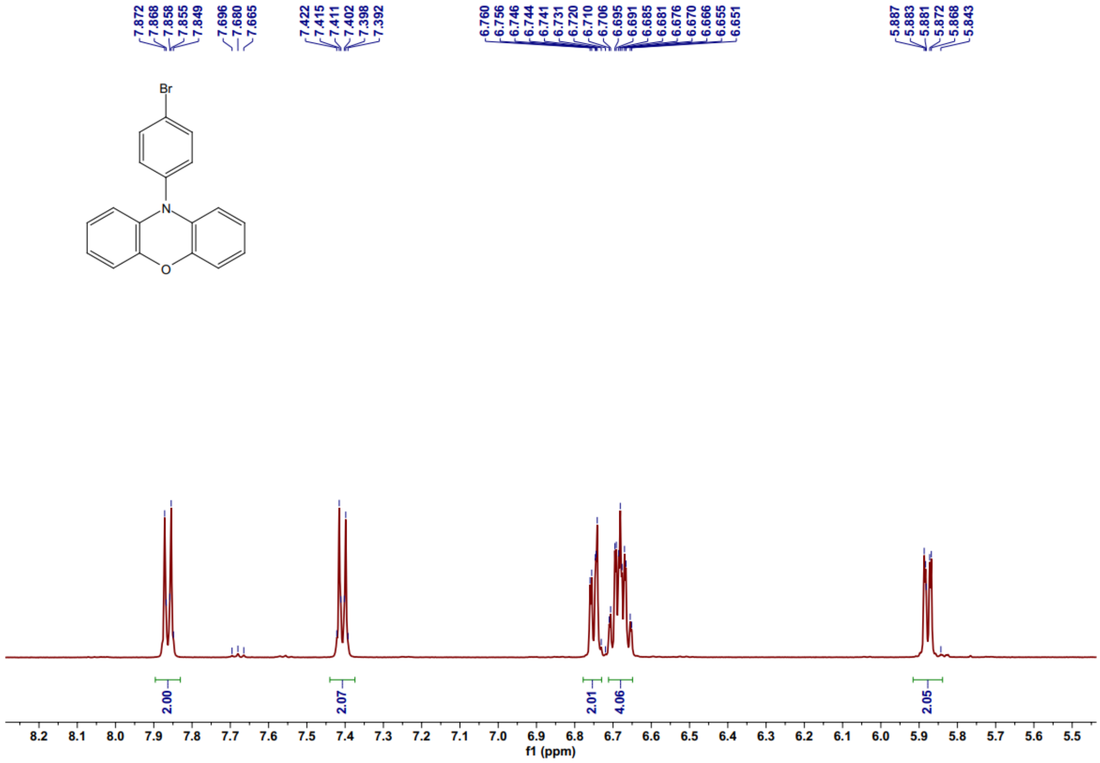

Figure **S10**. ^{1}H NMR spectra of **5** in $CDCl_3$.

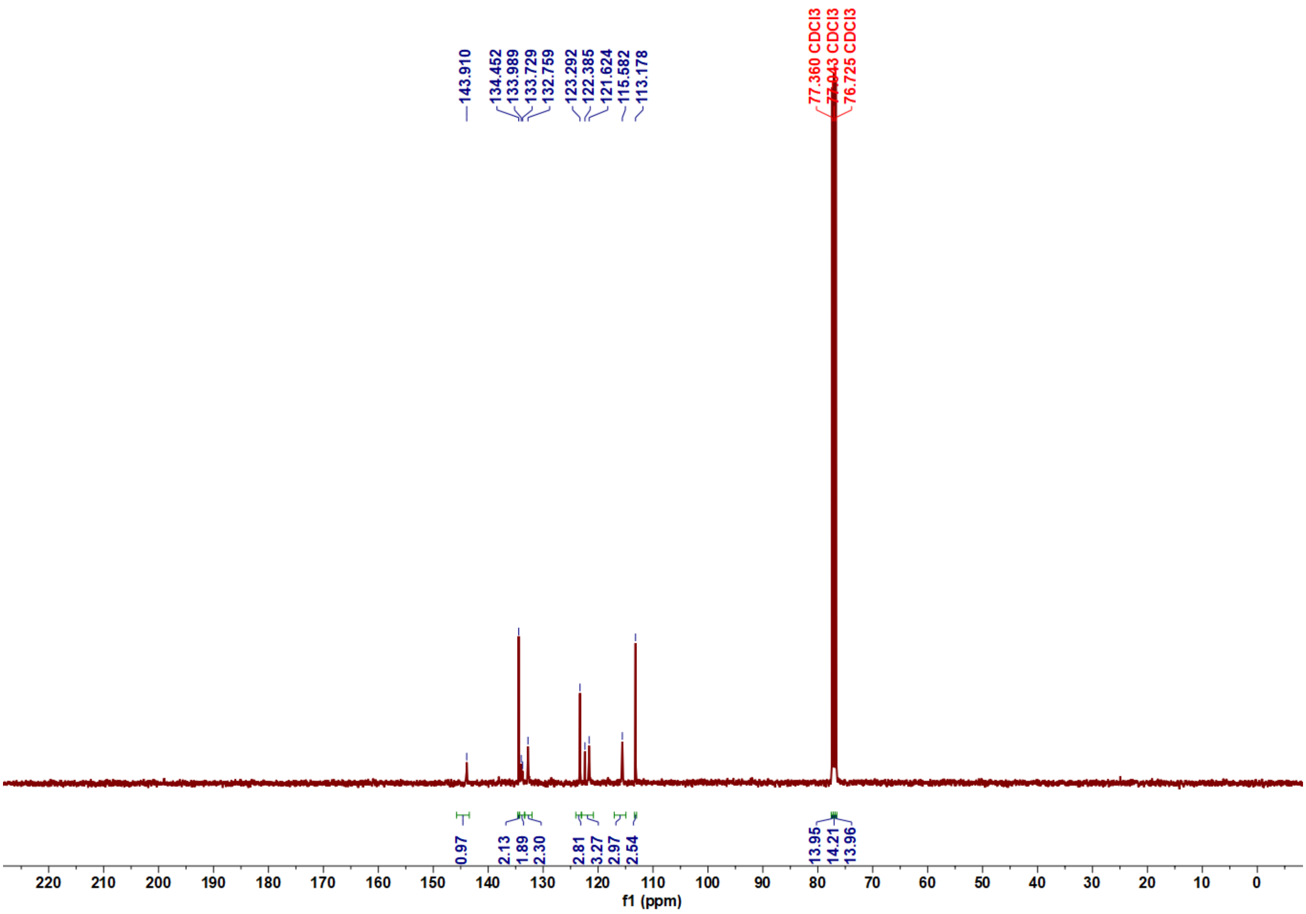

Figure **S11.** ^{13}C NMR spectra of **5** in $CDCl_3$.

Synthesis of 10-(4-(4,4,5,5-tetramethyl-1,3,2-dioxaborolan-2-yl)phenyl)-10H-phenoxazine (6):

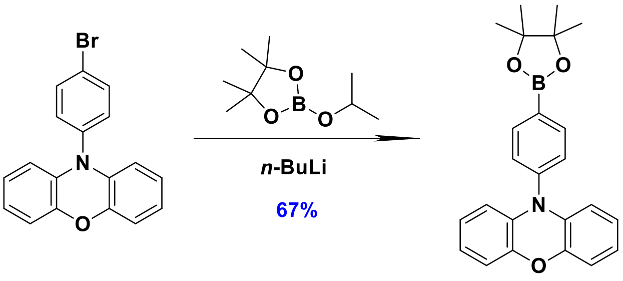

5 **6**

Compound **6** was synthesized according to the same procedure as described above for the synthesis of **4**, except that **5** (3.1 g, 9 mmol) was used as the reactant instead of **3**, yielding a white solid (yield =2.36 g).

10-(4-(4,4,5,5-tetramethyl-1,3,2-dioxaborolan-2-yl)phenyl)-10H-phenoxazine (6): R$_f$=0.3 (20% DCM/Hexane)**. Yield:** 67%. **Mp=**120-123 °C. **^{1}H NMR (400 MHz, CDCl$_3$):** δ 8.06 (d, *J* = 7.6 Hz, 2H), 7.37 (d, *J* = 7.9 Hz, 2H), 6.65 (dd, *J* = 43.1, 7.5 Hz, 6H), 5.93 (s, 2H), 1.29 (s, 12H) ppm. **^{13}C**

NMR (101 MHz, CDCl$_3$): δ = 137.50, 123.20, 113.31, 84.15, 83.53, 77.35, 77.04, 76.72, 25.05, 24.93 ppm. Analytical data matches that previously reported.[26]

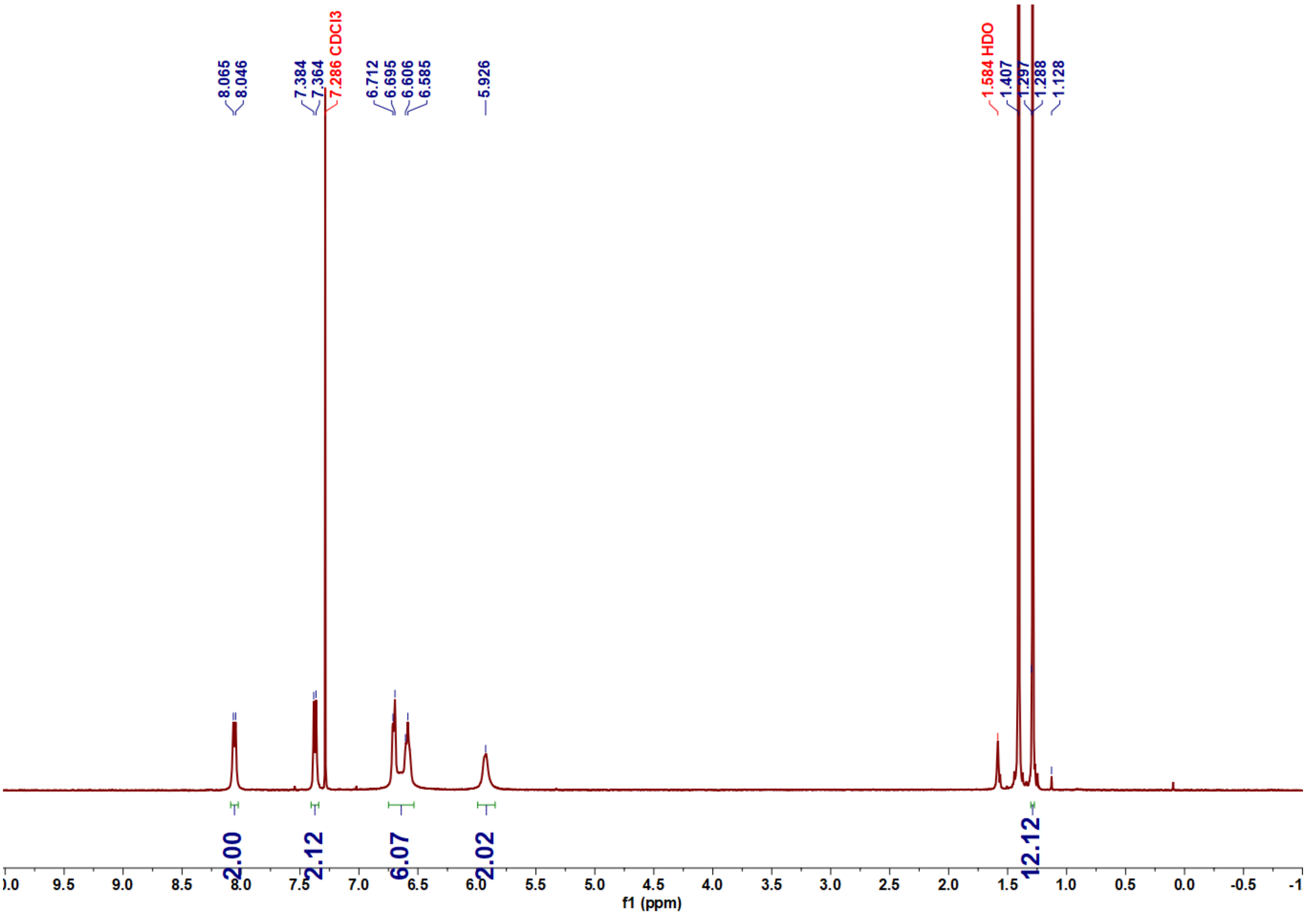

Figure **S12**. ^{1}H NMR spectra of **6** in CDCl$_3$.

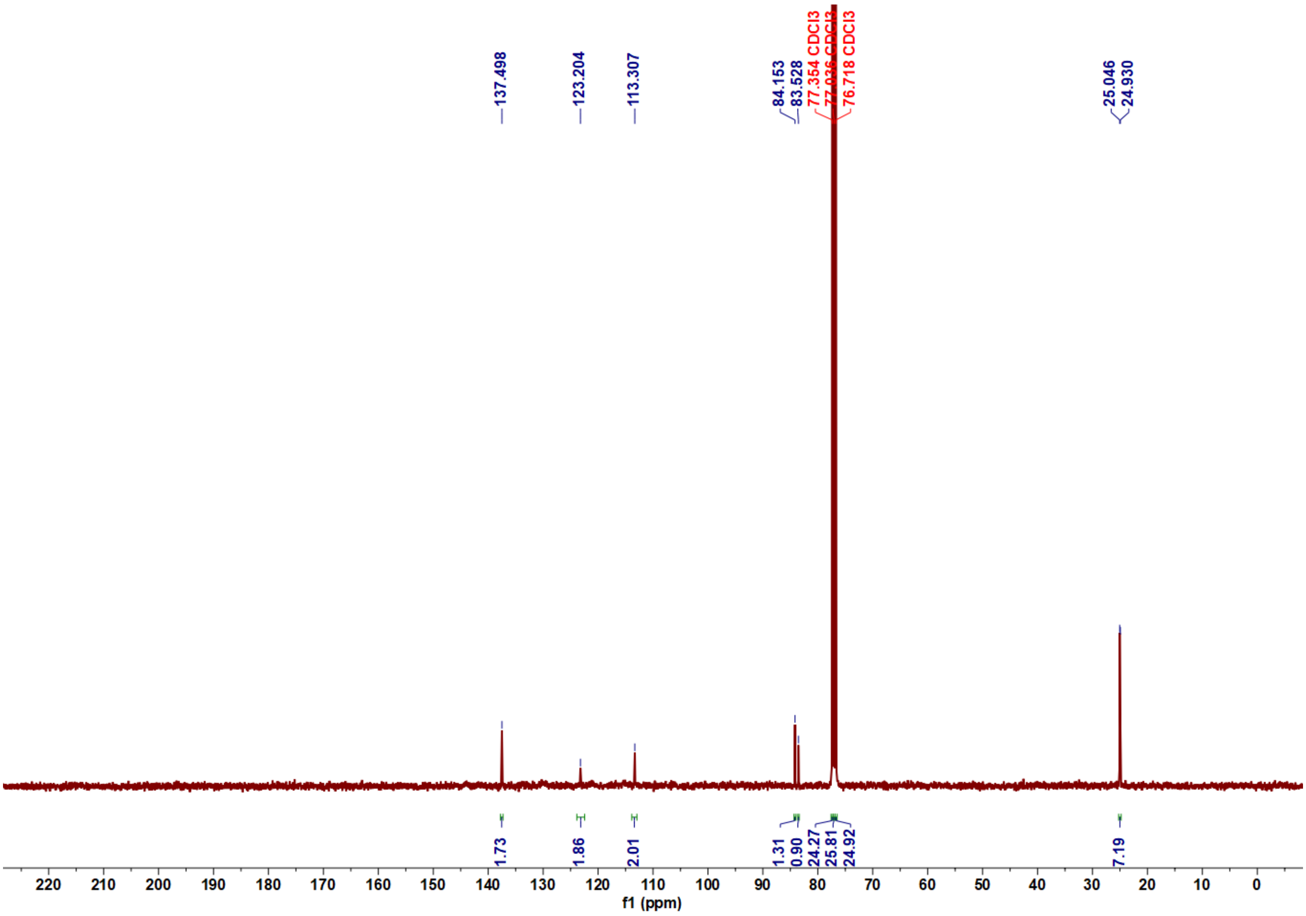

Figure **S13**. ^{13}C NMR spectra of **6** in $CDCl_3$

Synthesis of 9,9',9'',9'''-(dibenzo[*ghi,mno*]fluoranthene-1,2,5,6-tetrayltetrakis(benzene-4,1-diyl))tetrakis(9*H*-carbazole) (TCzPhCor):

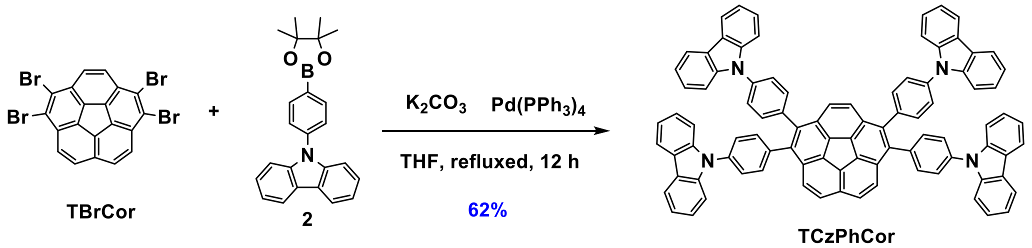

In a two-necked round bottom flask **TBrCor** (0.6 g, 1.06 mmol, 1.0 equiv.) and compound **2** (2 g, 5.3 mmol, 5.0 equiv.) were dissolved in a mixture of THF (40 mL) and a 2 M aqueous solution of potassium carbonate (10.0 mL). The mixture was degassed under nitrogen flow for 15 min, then tetrakis(triphenylphosphine)palladium(0) (185 mg, 0.3 mmol, 0.3 equiv.) was added under nitrogen. The reaction mixture was stirred at 70 °C for 12 h before cooling to room temperature. The reaction mixture was extracted with DCM (3 × 50 mL) and the combined organic layers were dried with

anhydrous sodium sulfate and concentrated under reduced pressure. The crude product was purified by silica gel flash column chromatography using DCM : hexane = 2:8 as eluent to give final compound as light-yellow solid (yield = 0.798 g).

9,9',9'',9'''-(dibenzo[*ghi,mno*]fluoranthene-1,2,5,6-tetrayltetrakis(benzene-4,1-diyl))tetrakis(9*H*-carbazole) (TCzPhCor): R_f = 0.6 (25% DCM/Hexane). **Yield**: 62%. **Mp** = 264-265 °C. **^{1}H NMR (500 MHz, $CDCl_3$)**: δ 8.22 – 8.16 (m, 8H), 8.05 (d, *J* = 8.8 Hz, 2H), 7.98 (s, 2H), 7.94 (d, *J* = 8.8 Hz, 2H), 7.67 – 7.60 (m, 16H), 7.50 – 7.43 (m, 8H), 7.37 – 7.29 (m, 16H) ppm. **^{13}C NMR (101 MHz, $CDCl_3$):** δ 140.81, 138.88, 138.51, 137.62, 136.61, 136.57, 135.56, 135.38, 134.49, 133.11, 130.89, 130.49, 130.05, 127.86, 127.44, 127.31, 126.46, 126.19, 123.49, 120.43, 120.11, 109.66, 109.63, 77.39, 77.07, 76.75 ppm. **HR-MS [M+H]$^+$ Calculated:** ($C_{92}H_{54}N_4$) 1215.4720; **Found:** 1215.4221. **Anal. Calcd. for $C_{92}H_{54}N_4$:** C, 90.91%; H, 4.48%; N, 4.61%. **Found**: C, 90.80%; H, 4.38%; N, 4.61%. HPLC analysis: 99.8% pure on HPLC analysis, retention time 10.3 minutes in 100% THF.

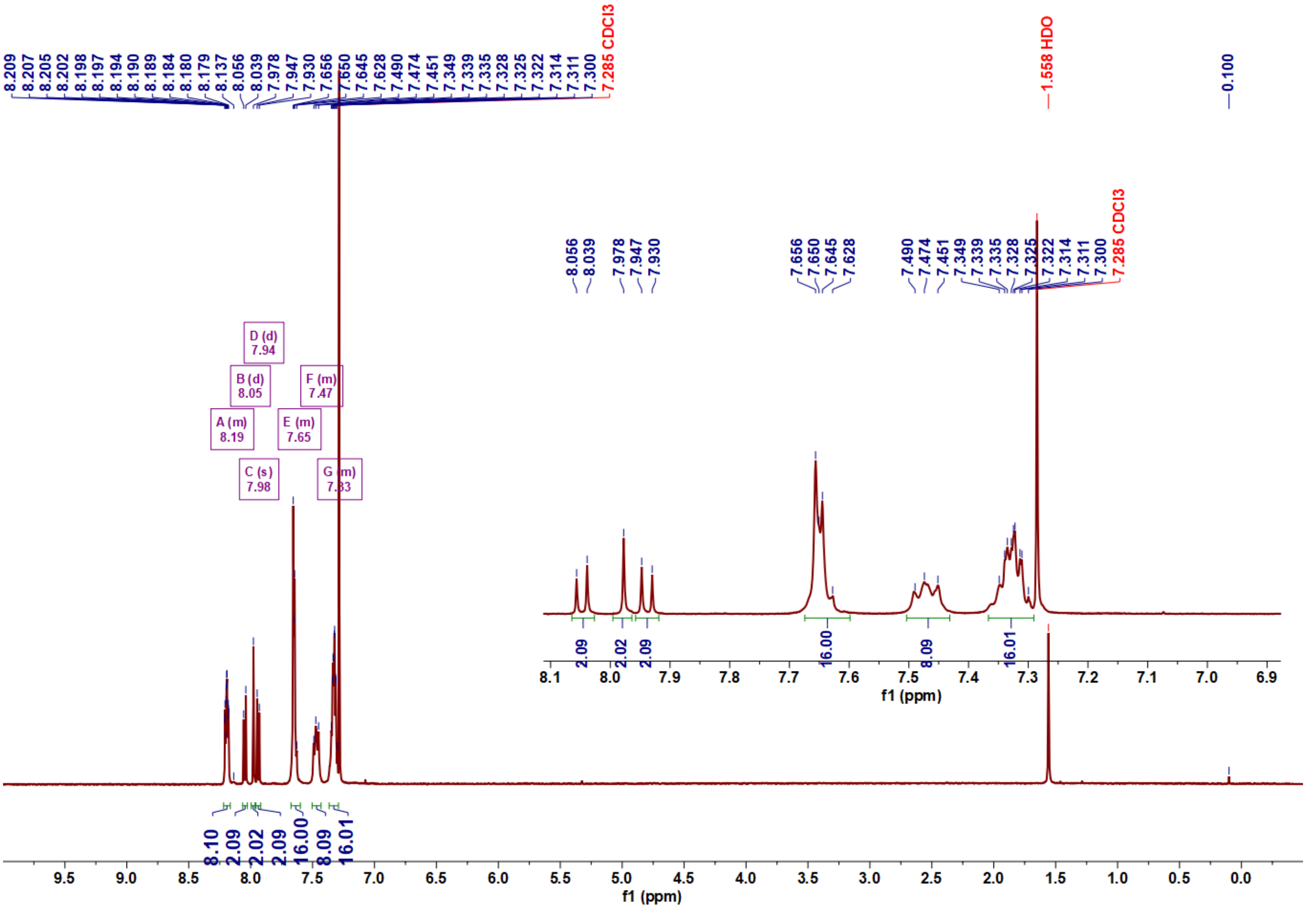

Figure **S14**. ^{1}H NMR spectra of **TCzPhCor** in $CDCl_3$.

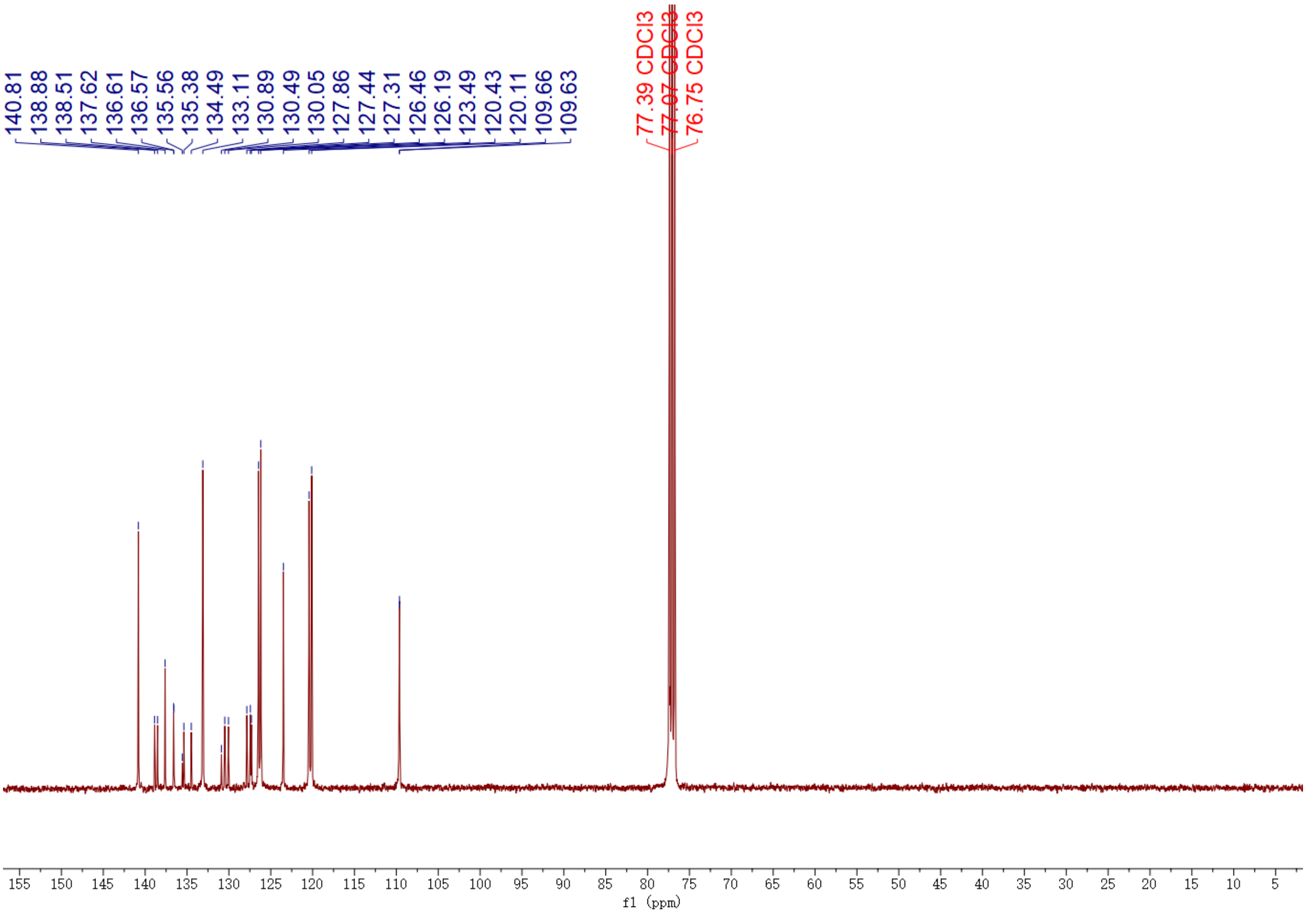

Figure **S15**. ^{13}C NMR spectra of **TCzPhCor** in $CDCl_3$.

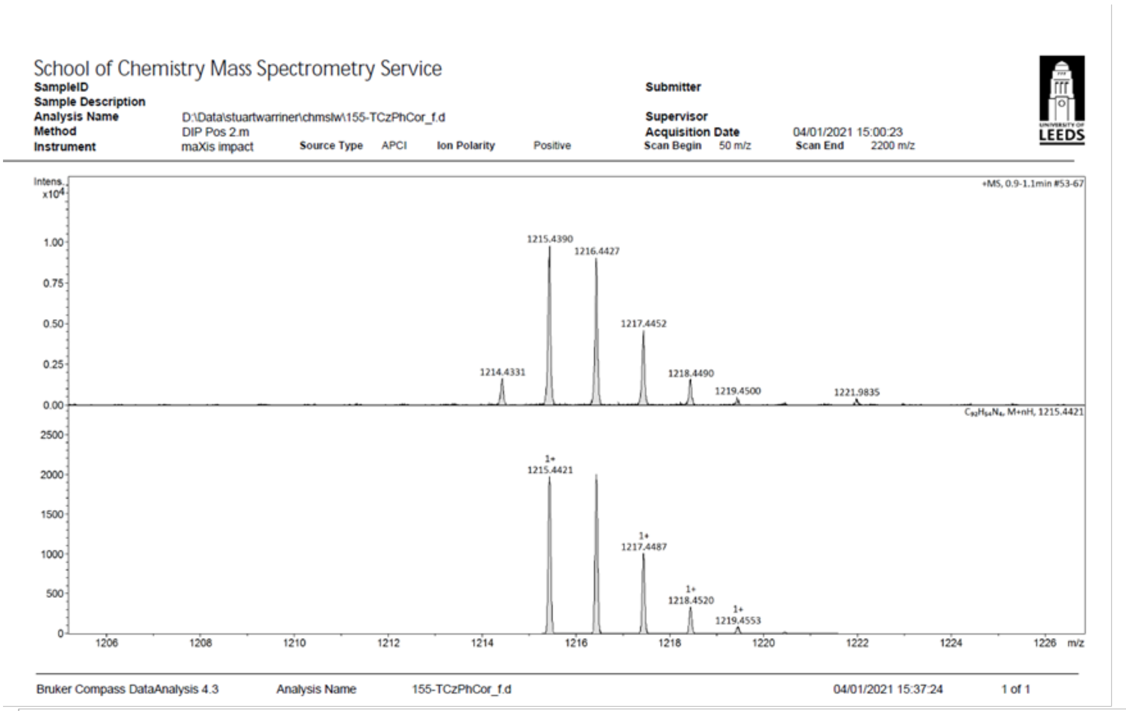

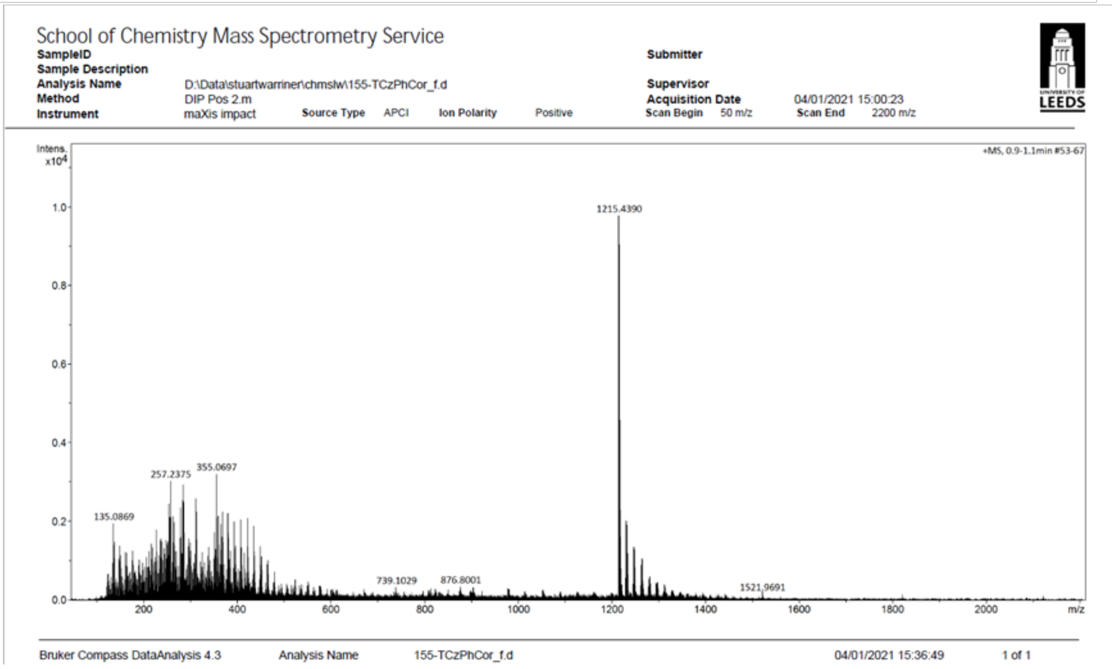

Figure **S16**. HRMS of **TCzPhCor**.

LONDON metropolitan university

Elemental Analysis Service

Please send completed form and samples to:

Stephen Boyer
School of Human Sciences
Science Centre
London Metropolitan University
29 Hornsey Road
London N7 7DD

Telephone: 020 7133 3605
Fax: 020 7133 2577
Email: s.boyer@londonmet.ac.uk

Sample submitted by: Changfeng Si	
Address: **EZC group, School of Chemistry, University of St Andrews, North Haugh,St Andrews, Fife, KY16 9ST**	
Telephone: 07519611858	Email: cs339@st-andrews.ac.uk
Date Submitted:	

Please submit ca. 5 mg of sample.

Sample Reference No.: I55-Cor4PhCz
Name of Compound: 9,9',9'',9'''-(dibenzo[ghi,mno]fluoranthene-1,2,5,6-tetrayltetrakis(benzene-4,1-diyl))tetrakis(9H-carbazole)
Molecular Formula: C92H54N4
Stability: air stable
Hazards: no hazards
Other Remarks:

Element	Expected %	Found (1)	Found (2)	
Carbon	90.91	90.76	90.83	
Hydrogen	4.48	4.35	4.41	
Nitrogen	4.61	4.57	4.65	

Authorising Signature:

Date Completed: '5/11/19	Signature:
Comments:	

Figure **S17**. Elemental analysis of **TCzPhCor**.

HPLC Trace Report31May2022

<Sample Information>

Sample Name	: TCzPhCor		
Sample ID	: TdmacPhCor		
Method Filename	: 100% THF 20 mins 280nm - DH.lcm		
Batch Filename	: 2pDMACBP-2pDMAC-1.lcb		
Vial #	: 1-24	Sample Type	: Unknown
Injection Volume	: 10 uL		
Date Acquired	: 31/05/2022 21:12:10	Acquired by	: System Administrator
Date Processed	: 31/05/2022 21:32:11	Processed by	: System Administrator

<Chromatogram>

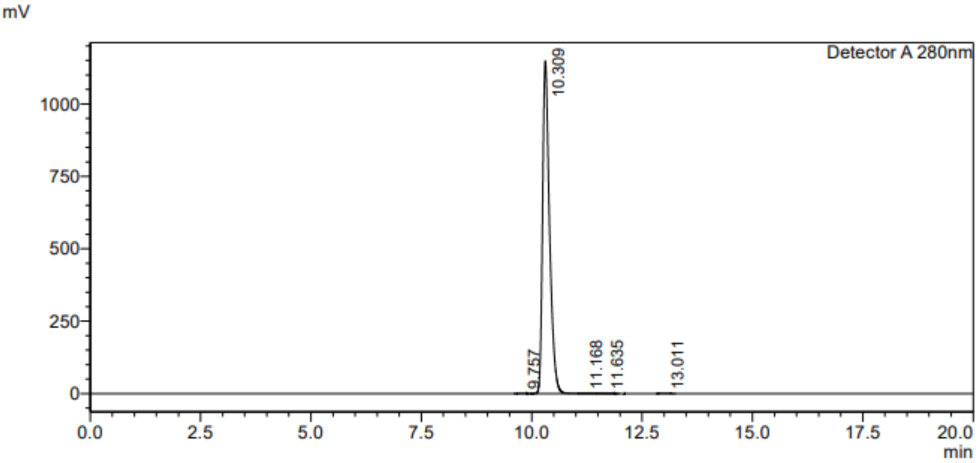

<Peak Table>

Detector A 280nm

Peak#	Ret. Time	Area	Height	Area%	Area/Height	Width at 5% Height
1	9.757	2143	193	0.017	11.096	--
2	10.309	12529545	1145260	99.890	10.940	0.384
3	11.168	4399	447	0.035	9.835	0.315
4	11.635	2855	226	0.023	12.648	0.370
5	13.011	4438	422	0.035	10.525	0.329
Total		12543380	1146548	100.000		

Figure **S18**. HPLC trace of **TCzPhCor**.

Synthesis of 10,10',10'',10'''-(dibenzo[*ghi,mno*]fluoranthene-1,2,5,6-tetrayltetrakis (benzene-4,1-diyl))tetrakis(9,9-dimethyl-9,10-dihydroacridine) (TDMACPhCor):

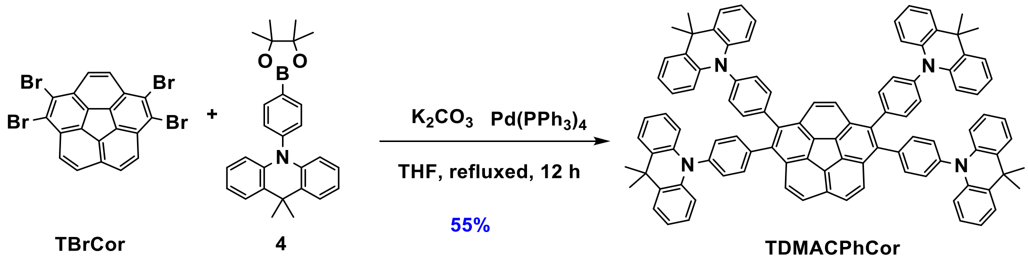

Compound **TDMACPhCor** was synthesized according to the same procedure as described above for the synthesis of **TCzPhCor**, except that **4** (2.2 g, 5.3 mmol) was used as the reactant instead of **2**, yielding a yellow solid (Yield = 0.805 g).

10,10',10'',10'''-(dibenzo[*ghi,mno*]fluoranthene-1,2,5,6-tetrayltetrakis(benzene-4,1-diyl))-tetrakis-(9,9-dimethyl-9,10-dihydroacridine) (TDMACPhCor): R_f = 0.6 (25% DCM/Hexane). **Yield**: 55%. **Mp** = 269-272 °C. **^{1}H NMR (500 MHz, $CDCl_3$)**: δ = 8.05 (d, *J* = 8.8 Hz, 2H), 7.97 (s, 2H), 7.94 (d, *J* = 8.8 Hz, 2H), 7.67 (d, *J* = 7.7 Hz, 8H), 7.47 (ddd, *J* = 7.5, 5.4, 1.6 Hz, 8H), 7.41 (t, *J* = 8.2 Hz, 8H), 6.91 – 6.74 (m, 16H), 6.35 (ddd, *J* = 13.4, 8.1, 1.3 Hz, 8H), 1.73 (d, *J* = 4.7 Hz, 24H) ppm. **^{13}C NMR (101 MHz, $CDCl_3$):** δ = 140.87, 140.84, 140.19, 140.17, 138.75, 138.49, 138.47, 135.49, 135.36, 134.52, 134.19, 130.86, 130.30, 130.13, 130.09, 129.94, 127.84, 127.47, 127.27, 126.62, 124.95, 120.65, 114.02, 114.00, 77.38, 77.06, 76.75, 36.05, 30.82, 30.79 ppm. **HR-MS [M+H]$^+$ Calculated:** ($C_{104}H_{78}N_4$) 1382.6226; **Found:** 1382.6197. **Anal. Calcd. for $C_{104}H_{78}N_4$:** C, 90.27%; H, 5.68%; N, 4.05%. **Found**: C, 89.98%; H, 5.76%; N, 3.94%. HPLC analysis: 99.2% pure on HPLC analysis, retention time 10.1 minutes in 100% THF.

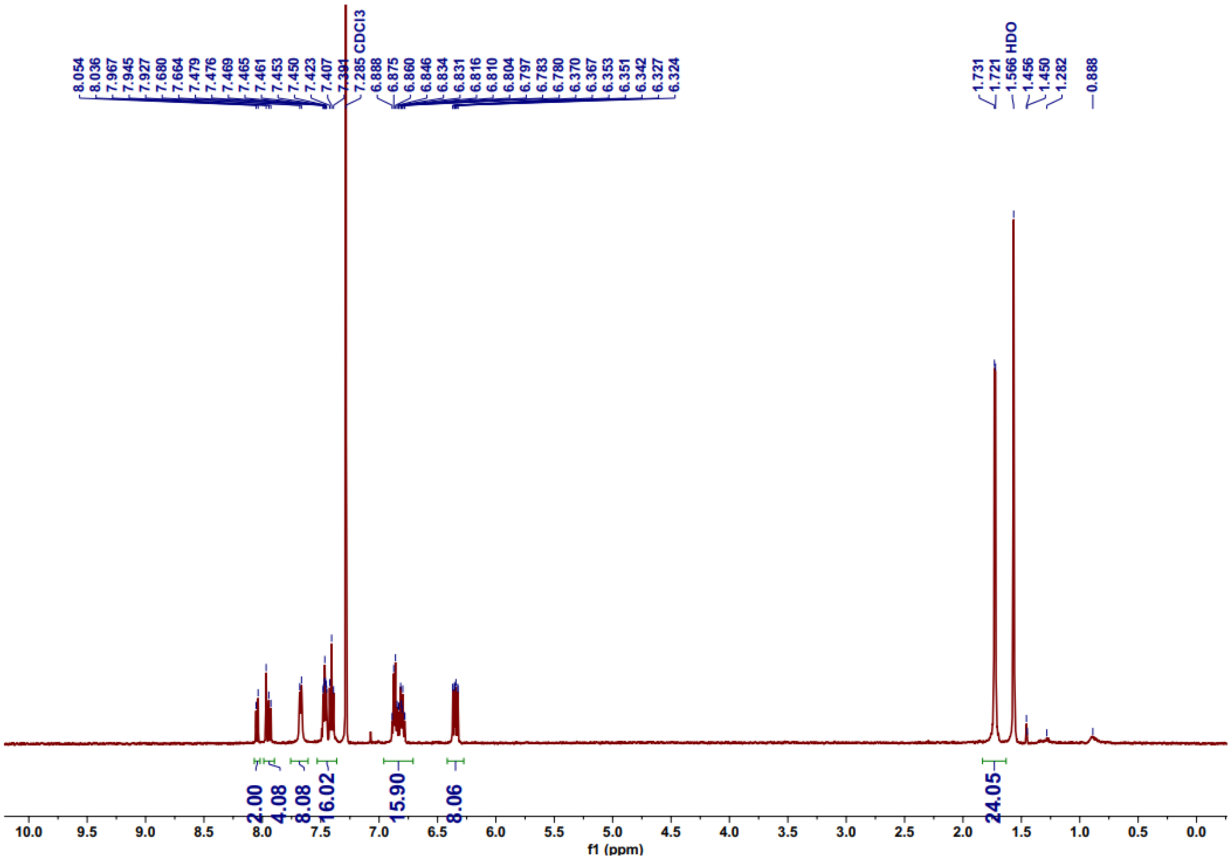

Figure **S19**. ^{1}H NMR spectra of **TDMACPhCor** in $CDCl_3$.

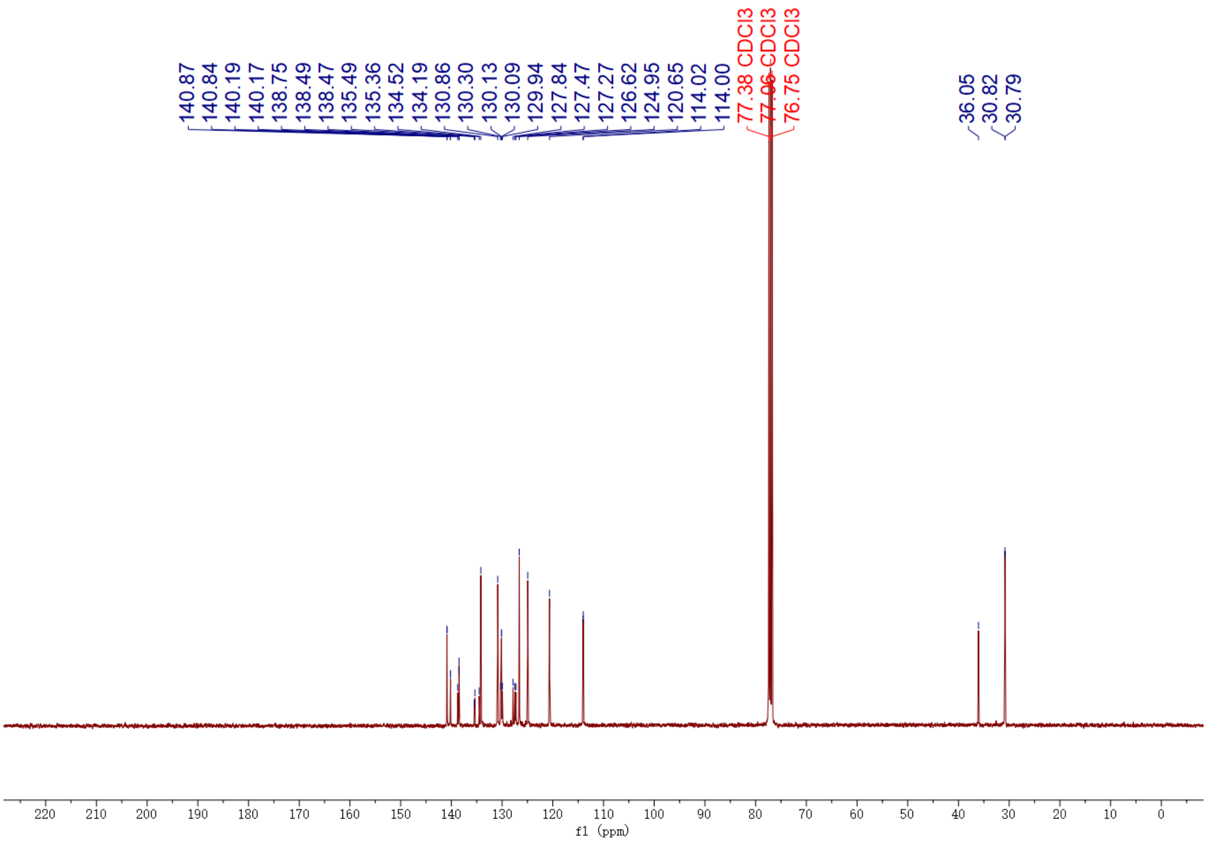

Figure **S20**. ^{13}C NMR spectra of **TDMACPhCor** in $CDCl_3$.

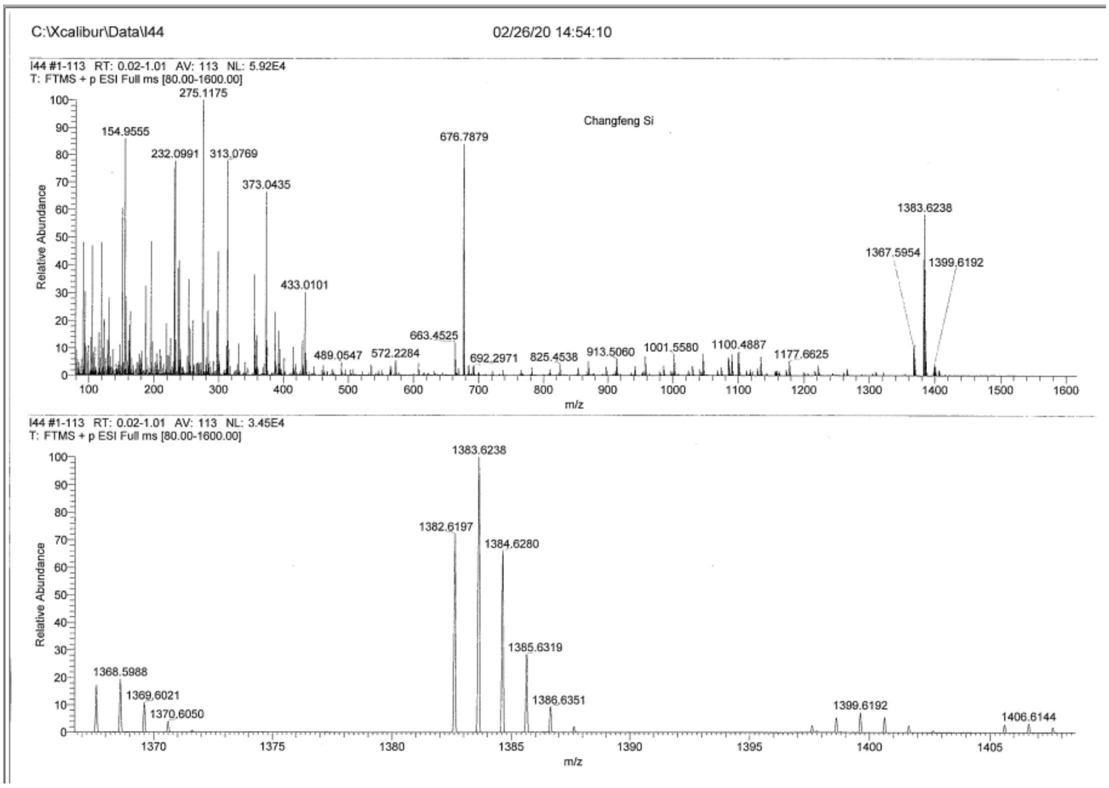

Figure **S21**. HRMS of **TDMACPhCor**.

Elemental Analysis Service Request Form

Researcher name Changfeng Si

Researcher email cs339@st-andrews.ac.uk

NOTE: Please submit ca. 10 mg of sample

**Sample reference number**	I44-Cor4PhDMAC
**Name of Compound**	10,10',10'',10'''-(dibenzo[*ghi,mno*]-fluoranthene-1,2,5,6-tetrayltetrakis(benzene-4,1-diyl))tetrakis(9,9-dimethyl-9,10-dihydroacridine)
**Molecular formula**	C104H78N4
**Stability**	
**Hazards**	
**Other Remarks**	

Analysis type:

Single ☐ **Duplicate** ☑ **Triplicate** ☐

Analysis Result:

Element	Expected %	Found (1)	Found (2)	Found (3)
Carbon	90.27	88.66	89.98	
Hydrogen	5.68	5.69	5.82	
Nitrogen	4.05	3.88	3.94	
Oxygen				

Authorising Signature:

**Date completed**	20·11·20
**Signature**	J-P[illegible]
**comments**	

Figure **S22**. Elemental analysis of **TDMACPhCor**.

HPLC Trace Report31May2022

<Sample Information>

Sample Name	: TdmacPhCor		
Sample ID	: TdmacPhCor		
Method Filename	: 100% THF 20 mins 280nm - DH.lcm		
Batch Filename	: 2pDMACBP-2pDMAC-1.lcb		
Vial #	: 1-23	Sample Type	: Unknown
Injection Volume	: 10 uL		
Date Acquired	: 31/05/2022 21:32:33	Acquired by	: System Administrator
Date Processed	: 31/05/2022 21:52:34	Processed by	: System Administrator

<Chromatogram>

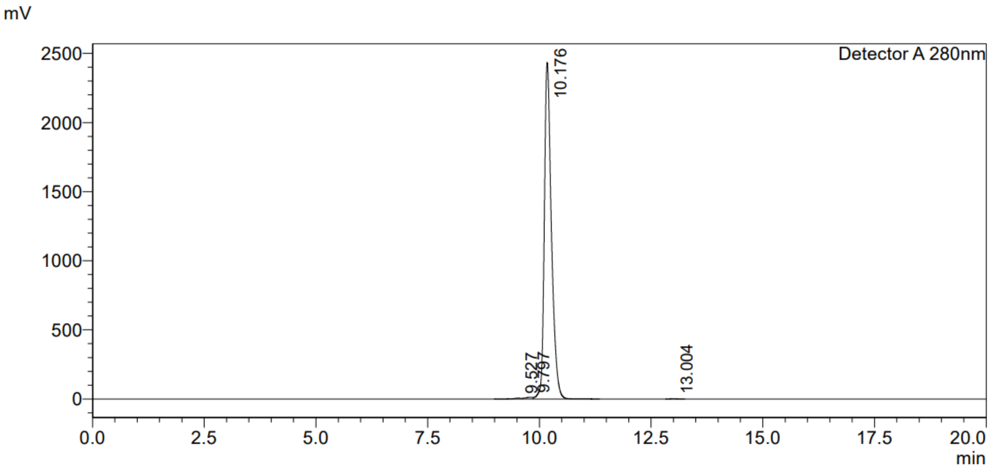

<Peak Table>

Detector A 280nm

Peak#	Ret. Time	Area	Height	Area%	Area/Height	Width at 5% Height
1	9.527	63600	5703	0.234	11.151	--
2	9.797	146176	12367	0.538	11.820	--
3	10.176	26965309	2428477	99.212	11.104	0.392
4	13.004	4412	406	0.016	10.861	0.341
Total		27179496	2446954	100.000		

Figure **S23**. HPLC trace of **TDMACPhCor**.

Synthesis of 10,10',10'',10'''-(dibenzo[*ghi,mno*]fluoranthene-1,2,5,6-tetrayltetrakis (benzene-4,1-diyl))tetrakis(*10H*-phenoxazine) (TPXZPhCor):

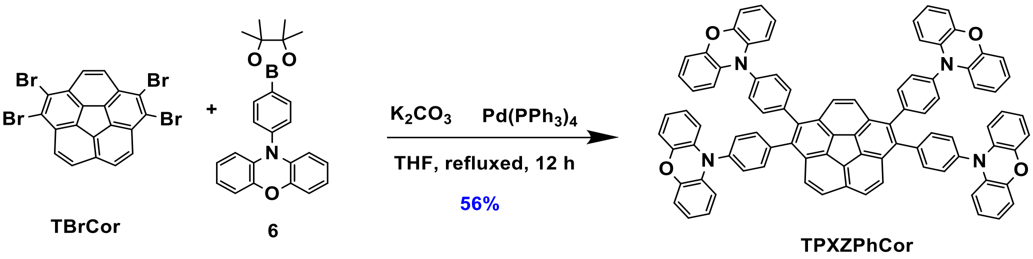

Compound **TPXZPhCor** was synthesized according to the same procedure as described above for the synthesis of **TCzPhCor**, except that **6** (2.0 g, 5.3 mmol) was used as the reactant instead of **4**, yielding a yellow solid (Yield = 0.758 g).

10,10',10'',10'''-(dibenzo[*ghi,mno*]fluoranthene-1,2,5,6-tetrayltetrakis(benzene-4,1-diyl))tetrakis-(*10H*-phenoxazine) (TPXZPhCor): **R_f**=0.6 (25% DCM/Hexane). **Yield**: 56%. **Mp** = 366-368 °C. **^{1}H NMR (500 MHz, $CDCl_3$)** δ 8.00 (d, *J* = 8.8 Hz, 2H), 7.83 (d, *J* = 10.7 Hz, 4H), 7.59 (d, *J* = 7.7 Hz, 8H), 7.37 (t, *J* = 8.4 Hz, 8H), 6.71 (dd, *J* = 7.7, 6.0 Hz, 8H), 6.62 (q, *J* = 7.2 Hz, 8H), 6.50 (dt, *J* = 14.3, 7.6 Hz, 8H), 5.91 (dd, *J* = 14.6, 7.9 Hz, 8H).ppm. **^{13}C NMR (101 MHz, $CDCl_3$):** δ 143.86, 138.72, 138.55, 138.21, 138.00, 135.41, 135.30, 134.43, 134.34, 134.18, 130.83, 130.36, 130.18, 129.81, 129.07, 128.26, 127.86, 127.33, 127.15, 125.32, 123.53, 121.41, 115.46, 113.11, 113.07, 77.36, 77.04, 76.73 ppm. **HR-MS [M+H]$^+$ Calculated:** ($C_{92}H_{54}N_4O_4$) 1279.4680; **Found:** 1279.3588. **Anal. Calcd. for $C_{92}H_{54}N_4O_4$:** C, 86.36%; H, 4.26%; N, 4.38%. **Found**: C, 86.23%; H, 4.25%; N, 4.89%. HPLC analysis: 99.7% pure on HPLC analysis, retention time 10.2 minutes in 100% THF.

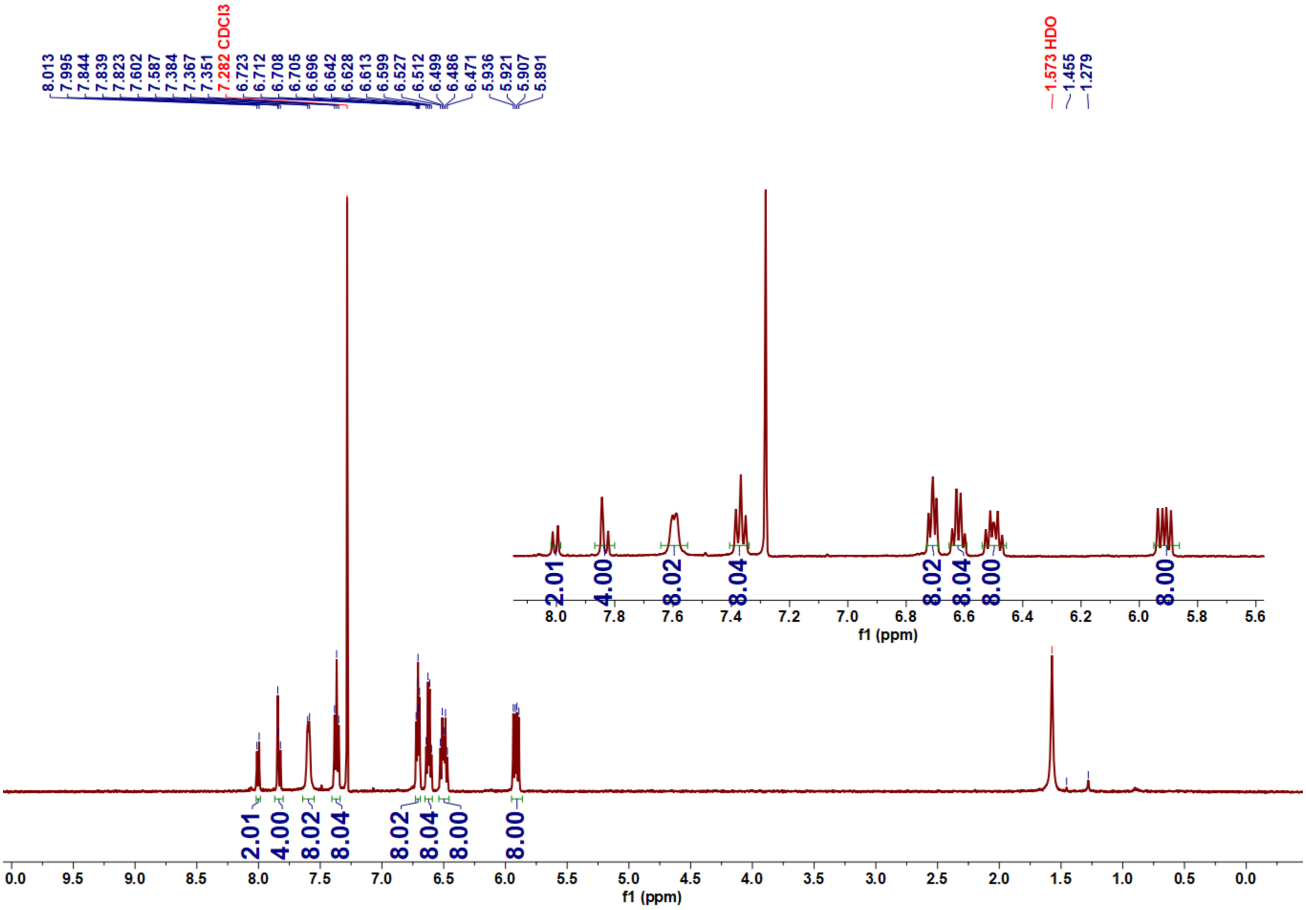

Figure **S24**. ^{1}H NMR spectra of **TPXZPhCor** in $CDCl_3$.

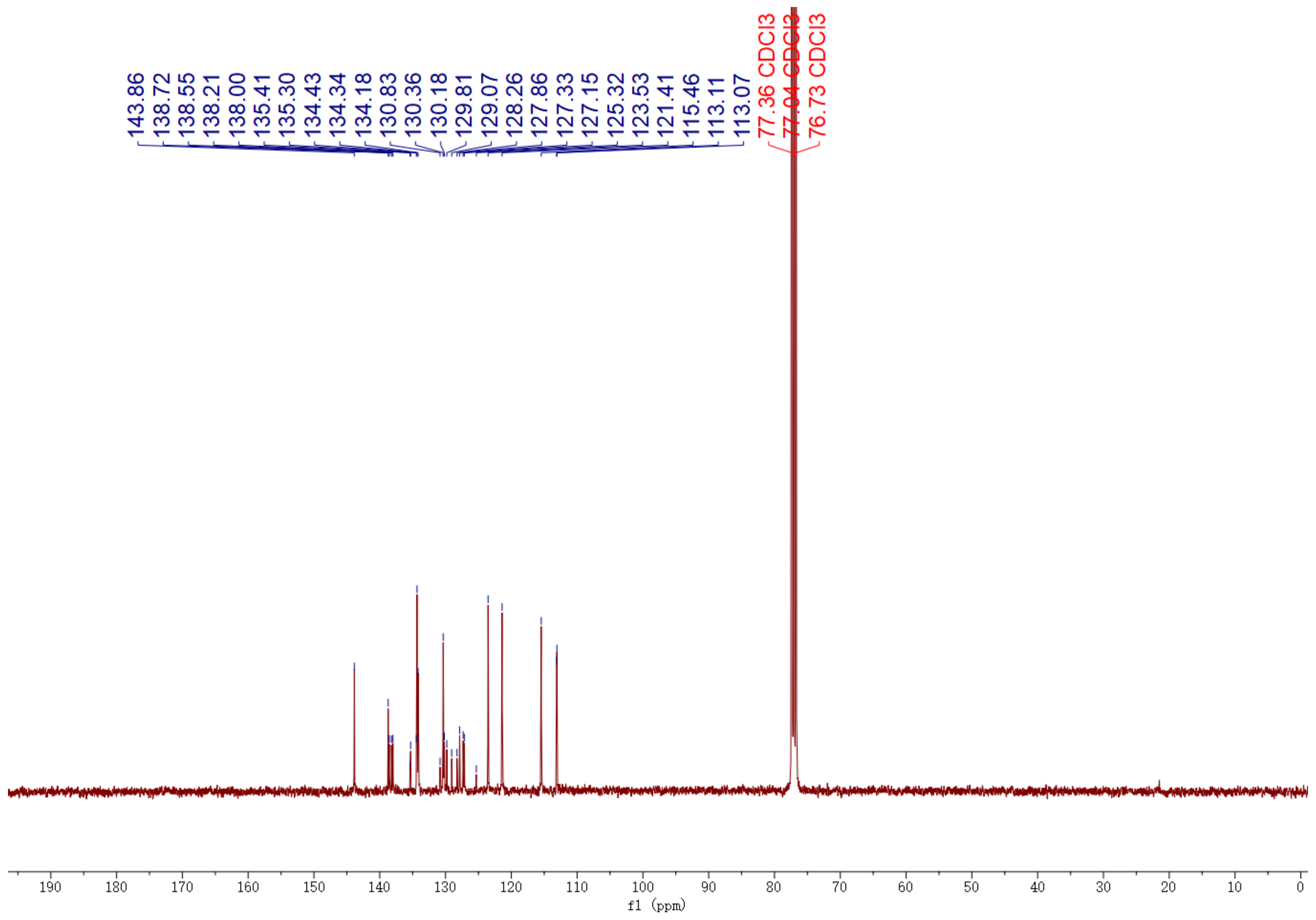

Figure **S25**. ^{13}C NMR spectra of **TPXZPhCor** in $CDCl_3$.

LONDON metropolitan university

Elemental Analysis Service

Please send completed form and samples to:

Stephen Boyer
School of Human Sciences
Science Centre
London Metropolitan University
29 Hornsey Road
London N7 7DD

Telephone: 020 7133 3605
Fax: 020 7133 2577
Email: s.boyer@londonmet.ac.uk

Sample submitted by: Changfeng Si	
Address: **EZC group, School of Chemistry, University of St Andrews, North Haugh,St Andrews, Fife, KY16 9ST**	
Telephone: 07519611858	Email: cs339@st-andrews.ac.uk
Date Submitted:	

Please submit ca. 5 mg of sample.

Sample Reference No.: I54-Cor4PhPXZ
Name of Compound: 10,10',10'',10'''-(dibenzo[ghi,mno]fluoranthene-1,2,5,6-tetrayltetrakis(benzene-4,1-diyl))tetrakis(10H-phenoxazine)
Molecular Formula: C92H54N4O4
Stability: air stable
Hazards: no hazards
Other Remarks:

Element	Expected %	Found (1)	Found (2)	
Carbon	86.36	86.23	86.18	
Hydrogen	4.25	4.22	4.29	
Nitrogen	5.00	4.82	4.92	

Authorising Signature:

Date Completed: 1511/9	Signature: [illegible]
Comments:	

Figure **S26**. Elemental analysis of **TPXZPhCor**.

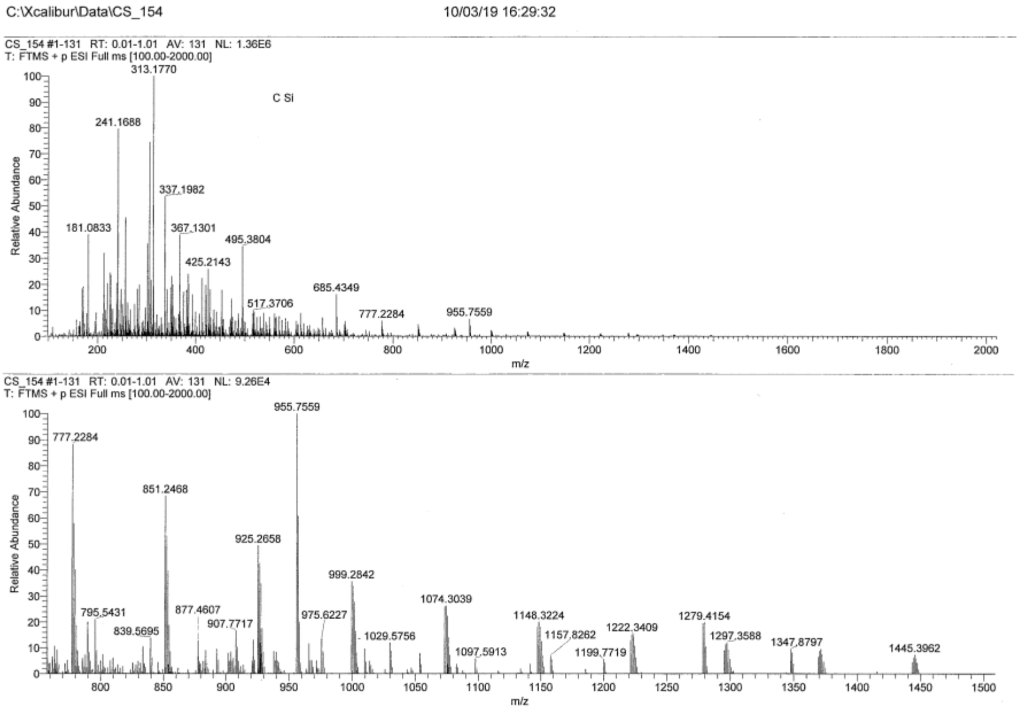

Figure **S27**. HRMS of **TPXZPhCor**.

HPLC Trace Report31May2022

<Sample Information>

Sample Name : TPXZPhCor
Sample ID : TPXZPhCor
Method Filename : 100% THF 20 mins 280nm - DH.lcm
Batch Filename : 2pDMACBP-2pDMAC-1.lcb
Vial # : 1-25 Sample Type : Unknown
Injection Volume : 10 uL
Date Acquired : 31/05/2022 20:48:12 Acquired by : System Administrator
Date Processed : 31/05/2022 21:08:14 Processed by : System Administrator

<Chromatogram>

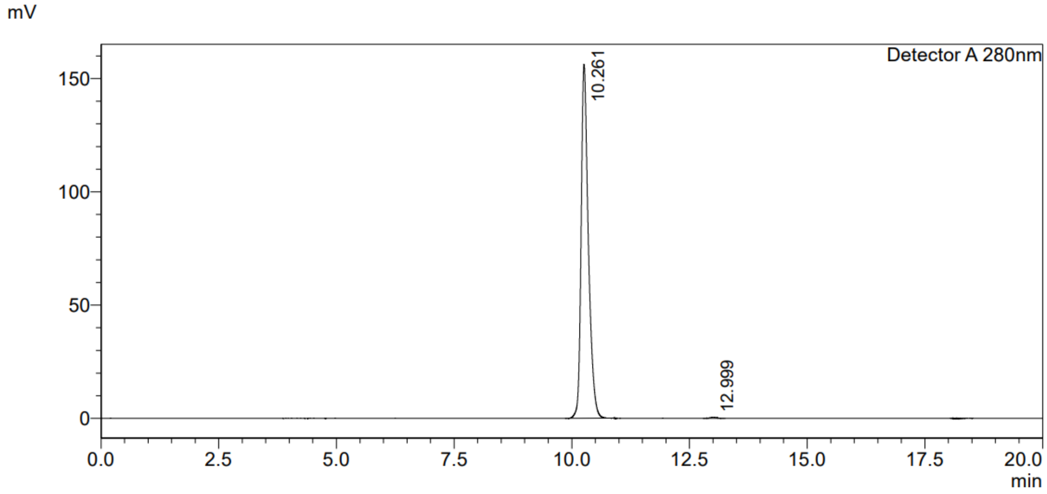

<Peak Table>

Detector A 280nm

Peak#	Ret. Time	Area	Height	Area%	Area/Height	Width at 5% Height
1	10.261	1673608	156147	99.706	10.718	0.386
2	12.999	4934	451	0.294	10.950	0.343
Total		1678542	156597	100.000		

Figure **S28**. HPLC trace of **TPXZPhCor**.

X-Ray structural analysis

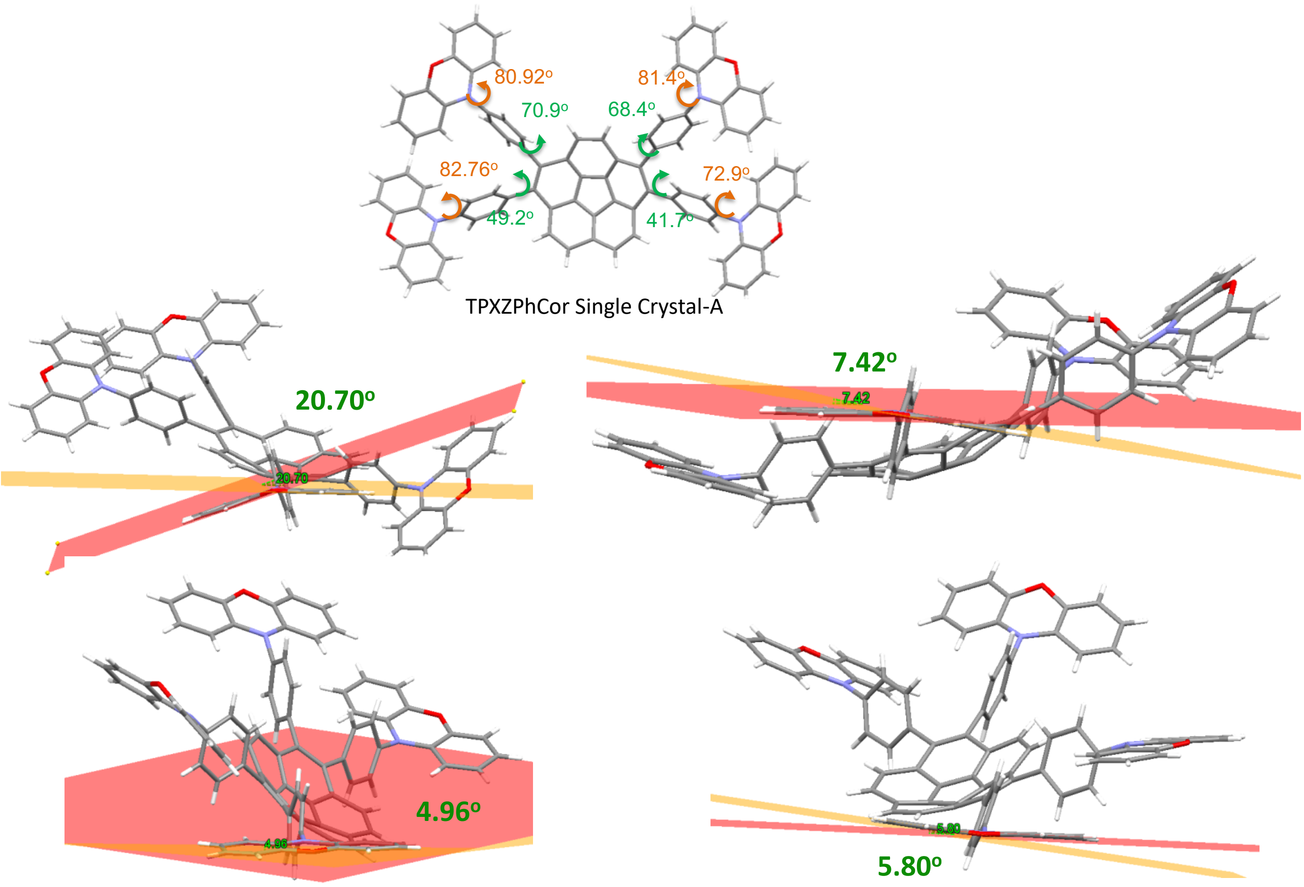

Figure **S29**. Four different orientations of phenylene and peripheral donors of one independent molecule of **TPXZPhCor** from the single crystal structure.

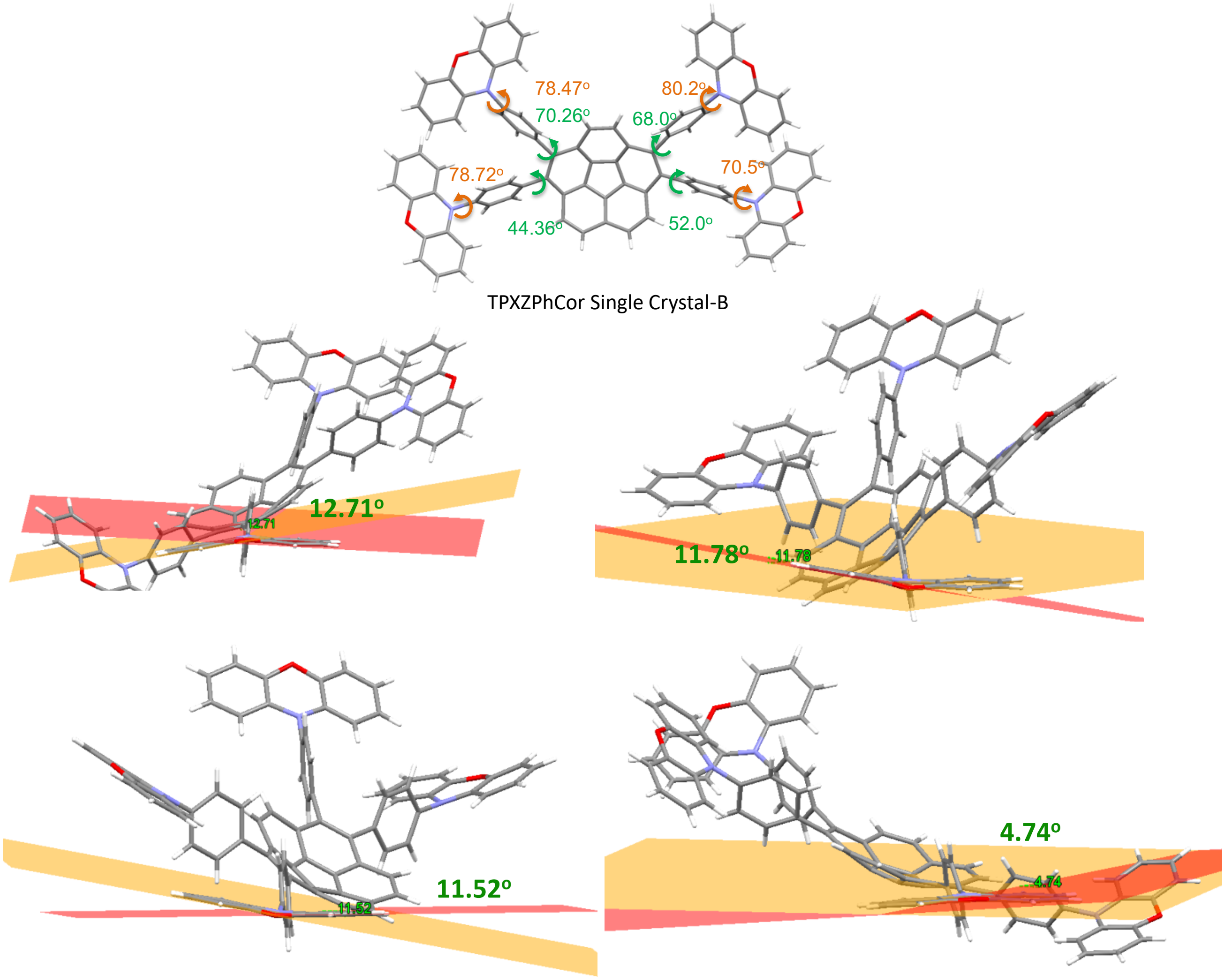

Figure **S30**. Four different orientations of phenylene and peripheral donors of one independent molecule of **TPXZPhCor** from the single crystal structure.

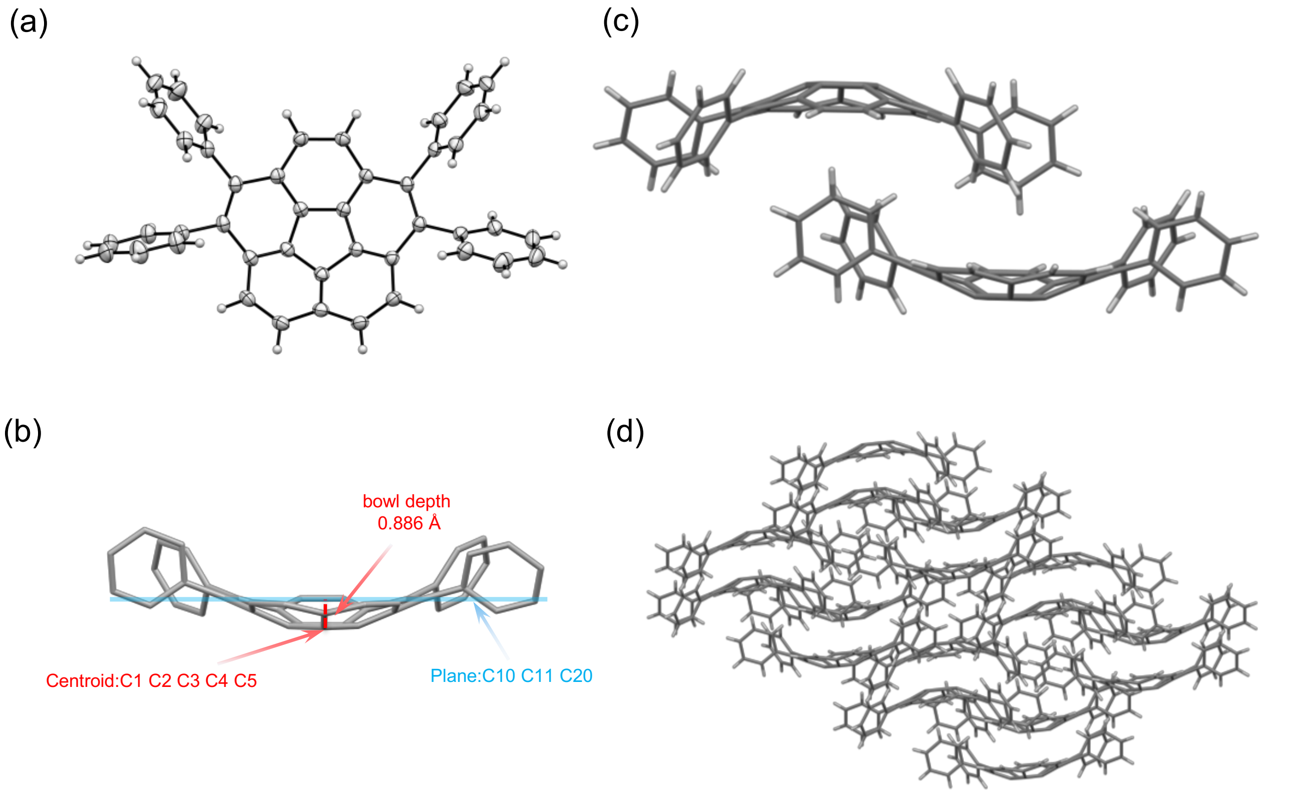

Figure **S31**. (a) Thermal ellipsoid plot of one independent molecule in the single crystal structure of **TPhCor**, ellipsoids are drawn at the 50% probability level, solvent molecules and the minor component of disorder have been omitted for clarity (b) **TPhCor** showing the bowl depth. (c) Views of the crystal structure of **TPhCor** showing interactions between adjacent molecules arranged in a convex-concave fashion. (d) Packing of **TPhCor** single crystals.

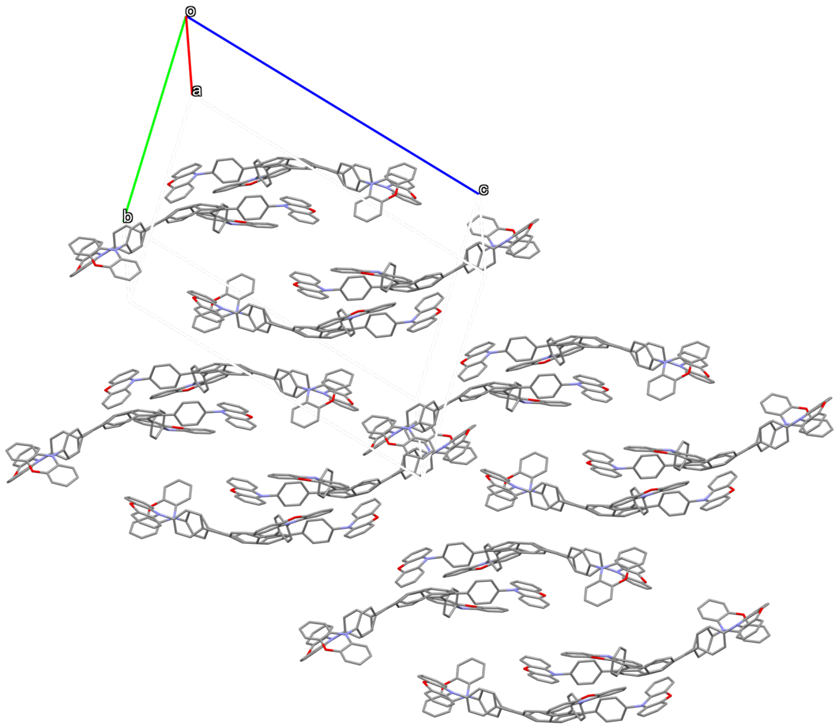

Figure **S32**. Packing of **TPXZPhCor**.

Theoretical calculation

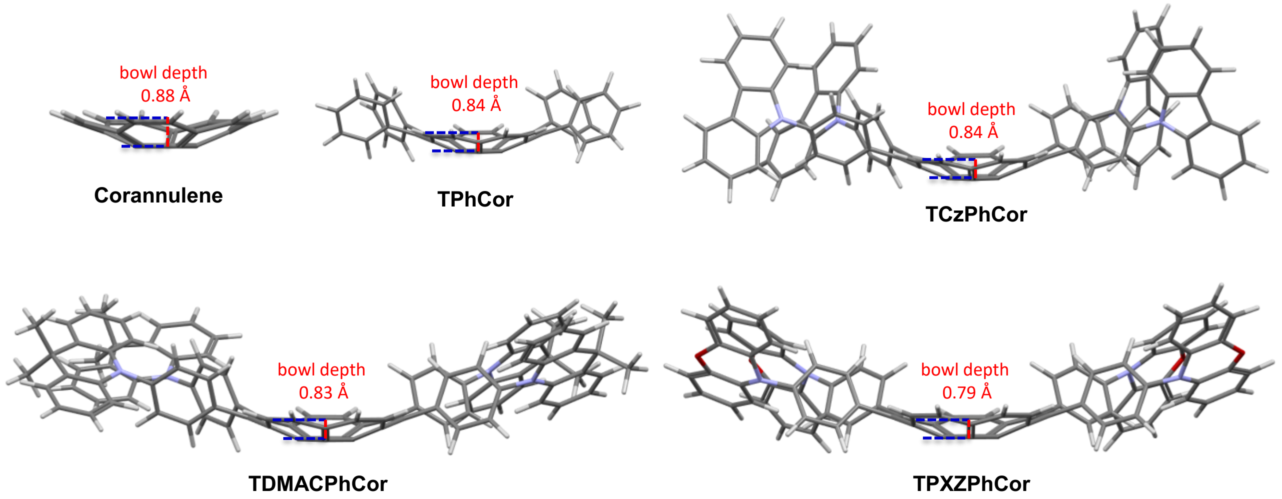

Figure **S33**. Bowl depth of corannulene, **TPhCor**, **TCzPhCor**, **TDMACPhCor** and **TPXZPhCor** at optimized S_0 geometries calculated at the DFT-M062X/6-31G(d,p) level.

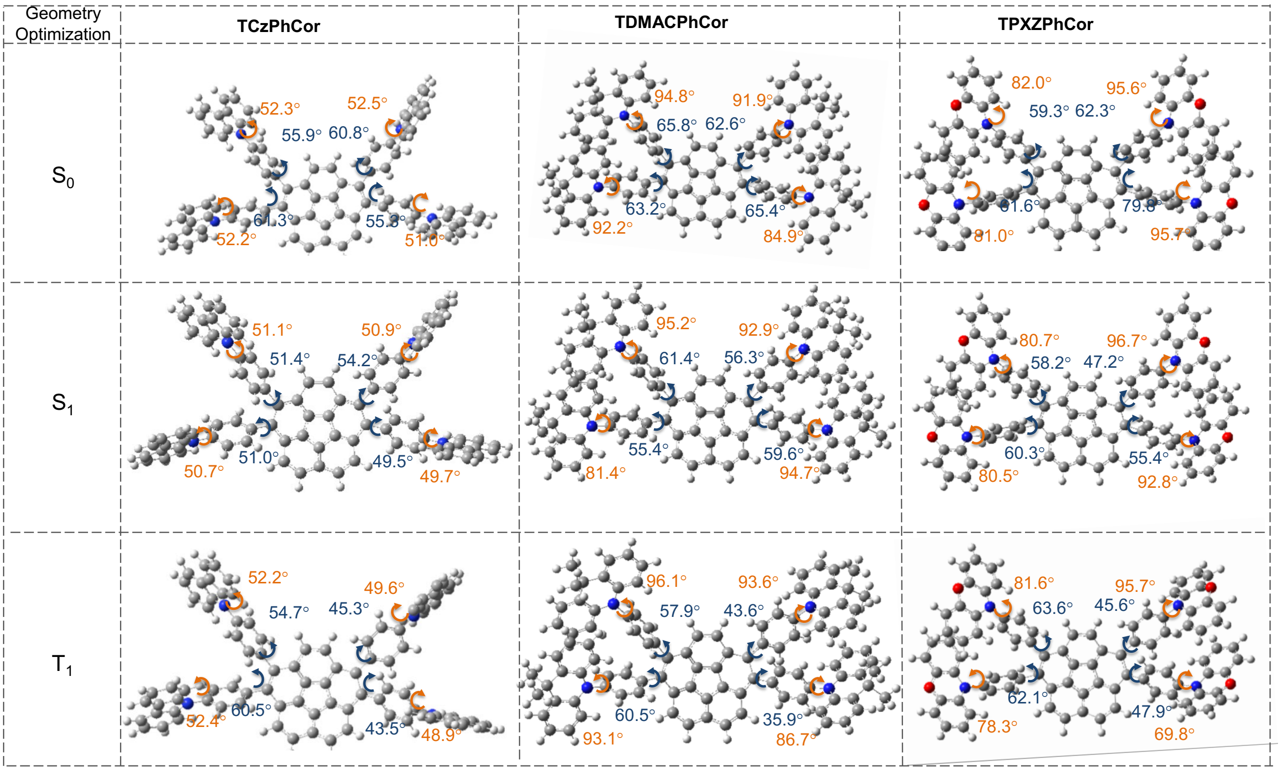

Figure **S34**. Optimized S_0, S_1 and T_1 geometries of **TCzPhCor**, **TDMACPhCor** and **TPXZPhCor** calculated at the DFT-M062X/6-31G(d,p) level (dihedral angles between corannulene and the π bridge in dark blue color; dihedral angles between π bridge and the donor N-heteroaromatics in orange color).

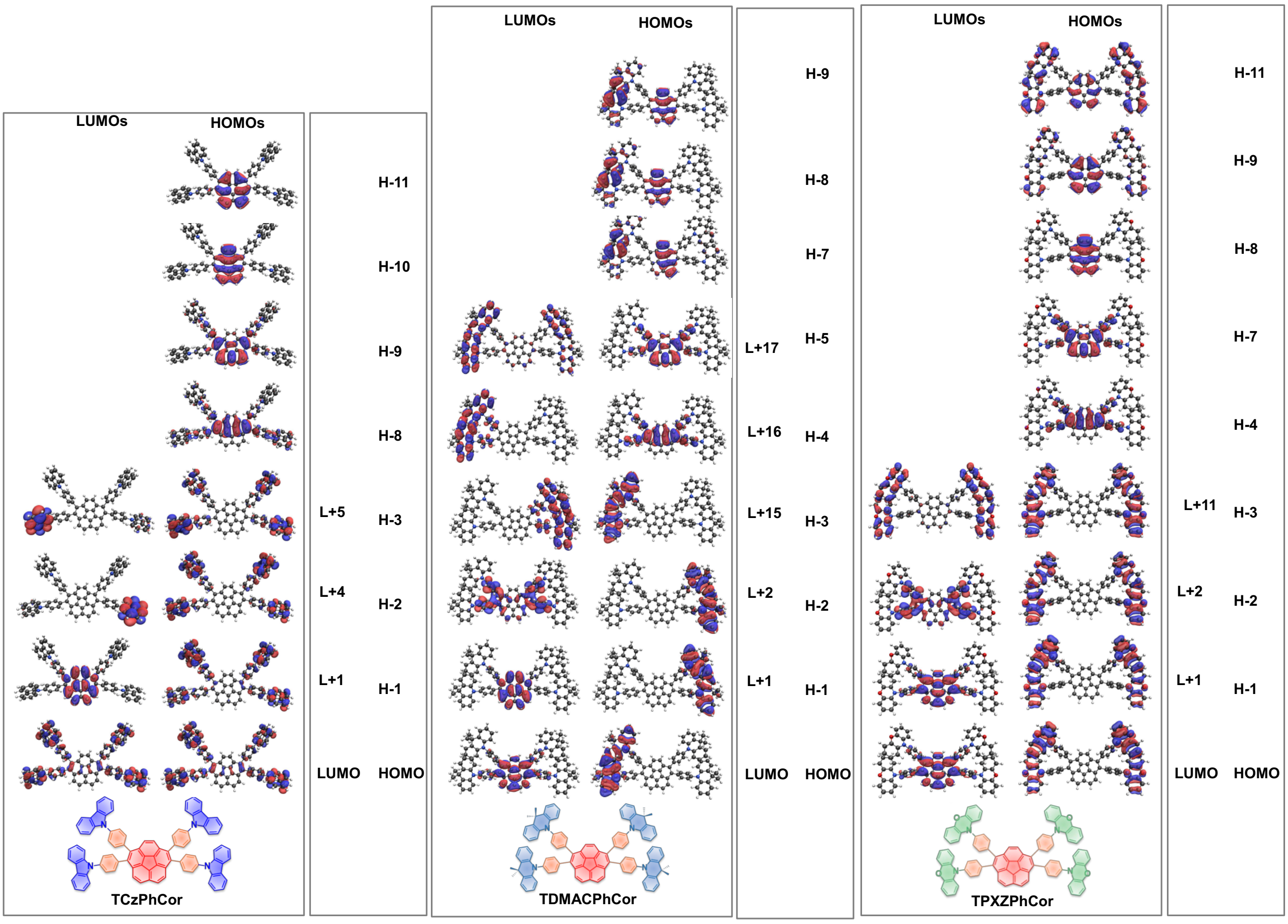

Figure **S35**. Frontier molecular orbital distributions of **TCzPhCor**, **TDMACPhCor** and **TPXZPhCor** (isovalue= 0.02).

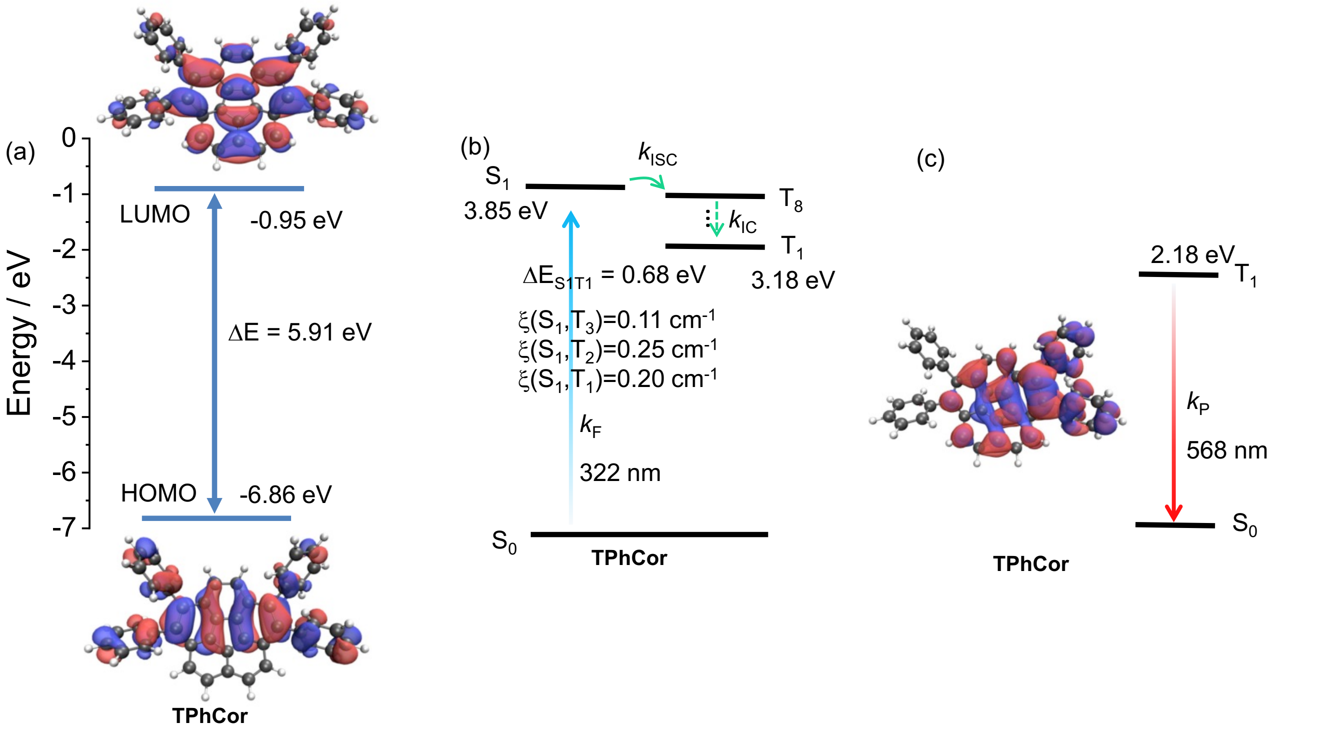

Figure **S36**. (a) HOMOs and LUMOs (isovalue: 0.02) and (b) vertical excitation energy levels of **TPhCor** calculated at the optimized S_0 geometry in the gas phase at the TDA-DFT-M062X/6-31G(d,p) level. (c) Natural transition orbitals (unoccupied (hole) (blue) & occupied (electron) (red), isovalue: 0.02) and T_1 vertical emission energies of **TPhCor** calculated in the gas phase at the T_1 optimized.

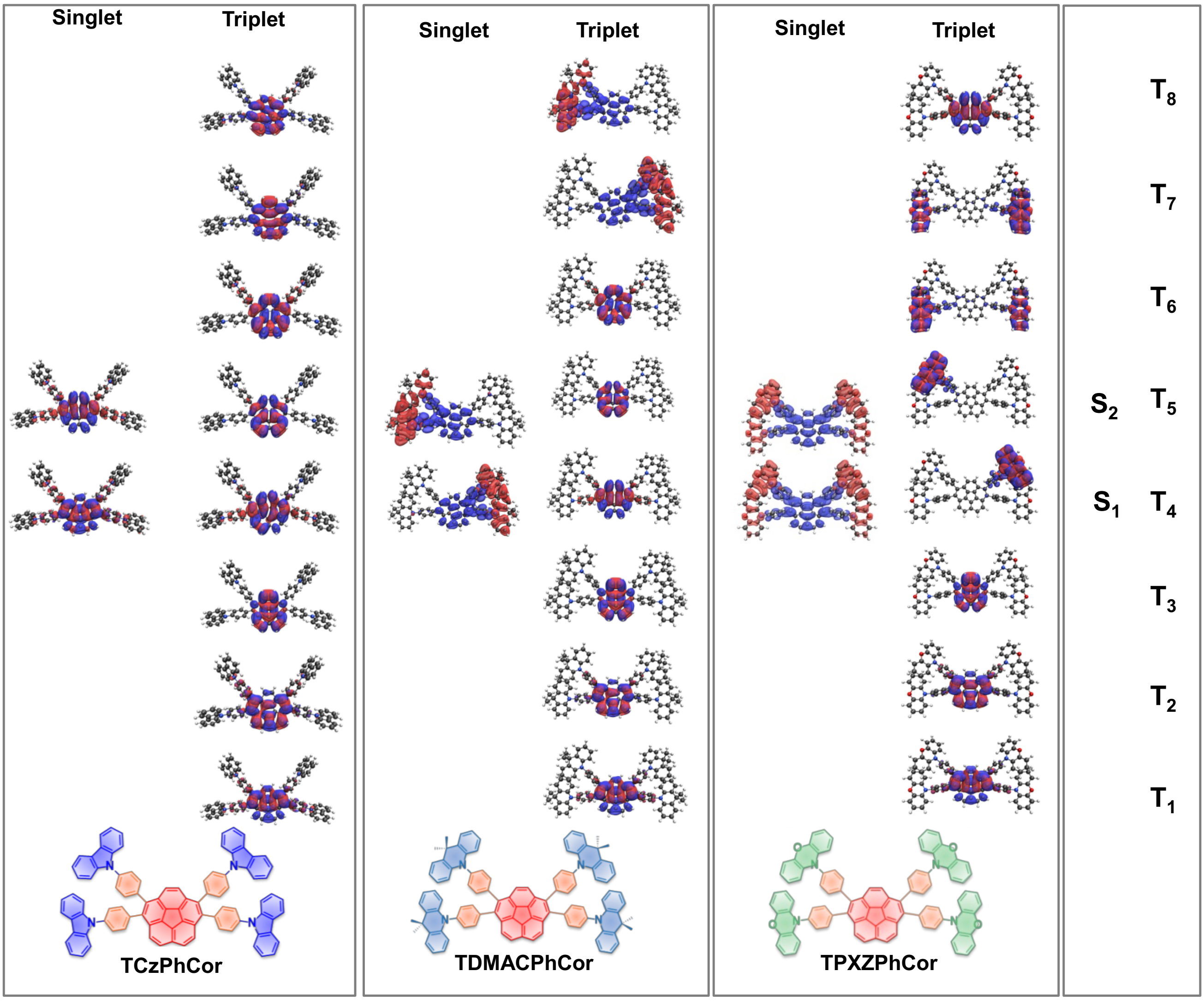

Figure **S37**. Natural Transition Orbitals hole (blue) and electron (red) distributions of **TCzPhCor**, **TDMACPhCor** and **TPXZPhCor** calculated at the optimized S_0 geometry in the gas phase at the TDA-DFT-M062X/6-31G(d,p) level.

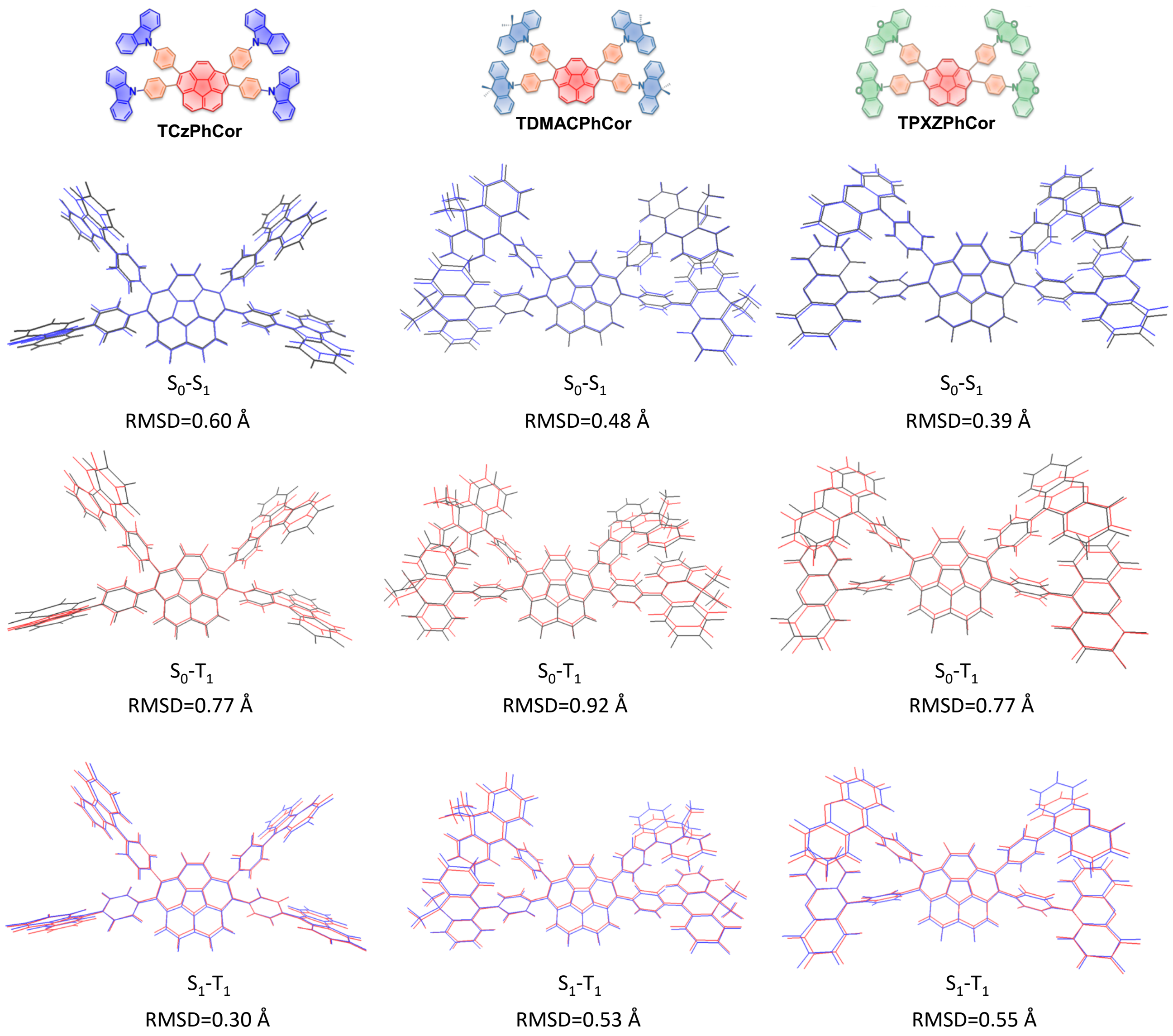

Figure **S38**. Calculated geometric changes between S_0 (black), S_1 (blue) and T_1 (red) states.

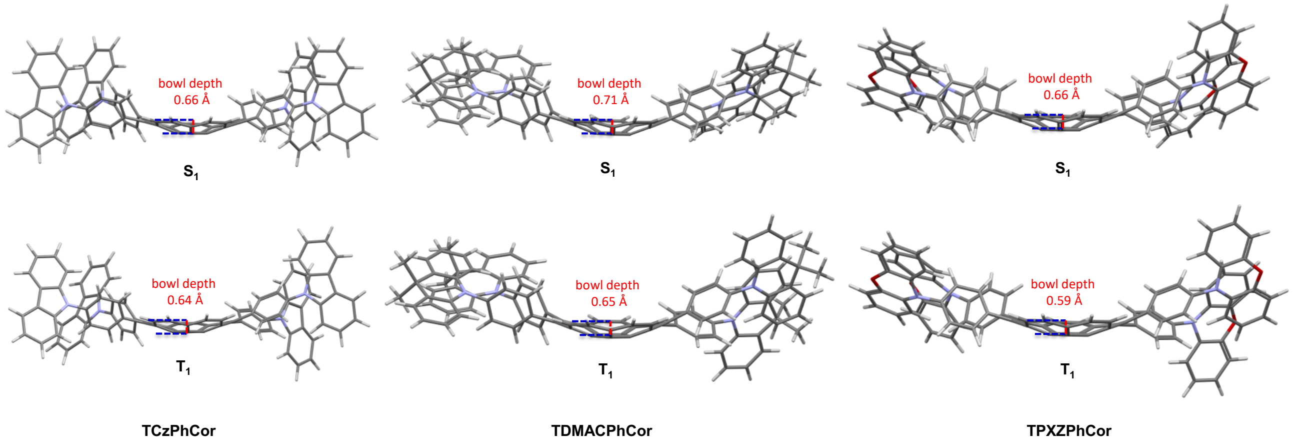

Figure **S39**. Bowl depth of **TCzPhCor**, **TDMACPhCor** and **TPXZPhCor** at optimized S_1 and T_1 geometries calculated at the DFT-M062X/6-31G(d,p) level.

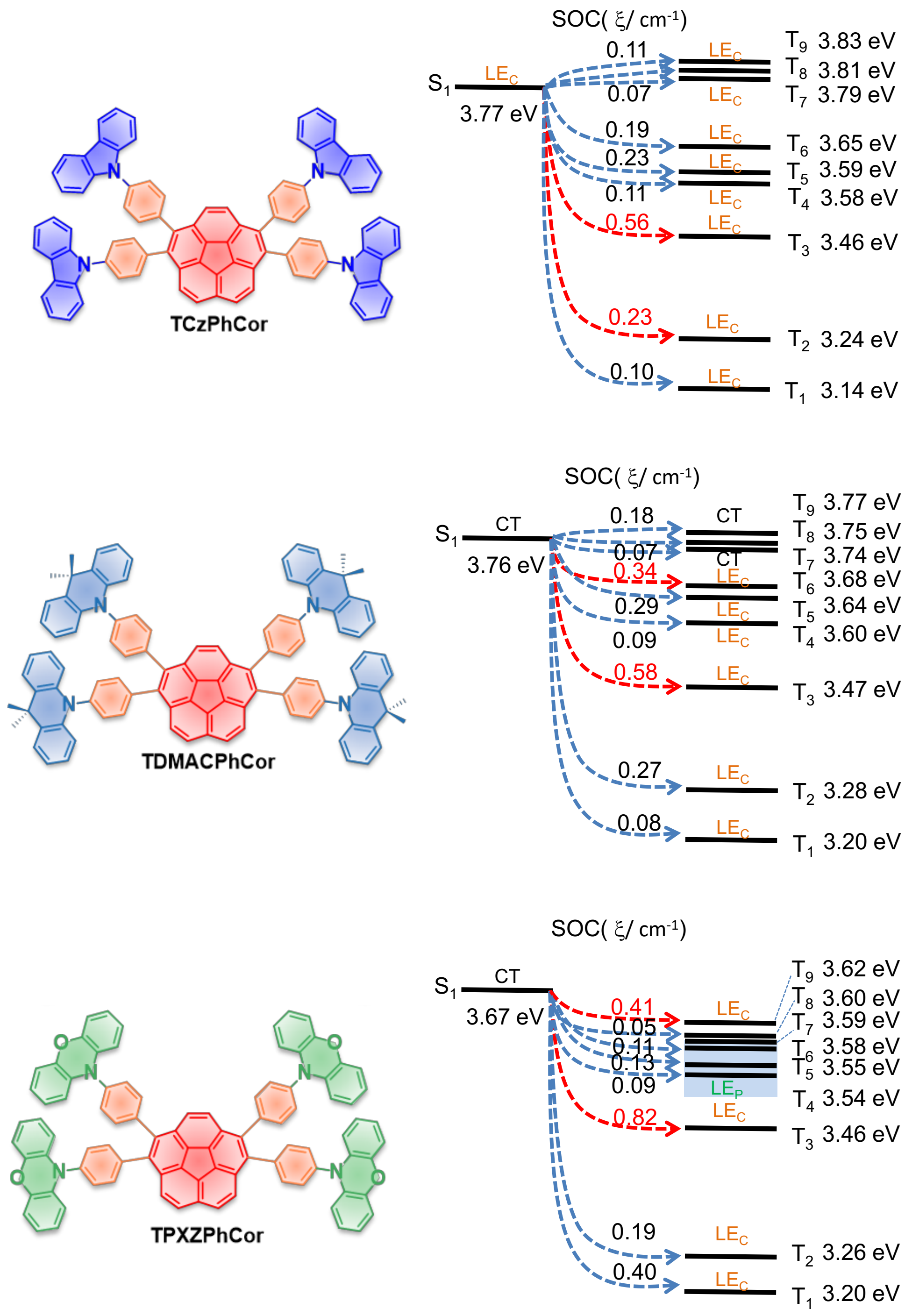

Figure **S40**. Vertical excitation energy levels of **TCzPhCor**, **TDMACPhCor** and **TPXZPhCor** calculated at the S_0 geometry with associated spin-orbit coupling constants calculated at the optimized S_1 geometry.

Electrochemistry

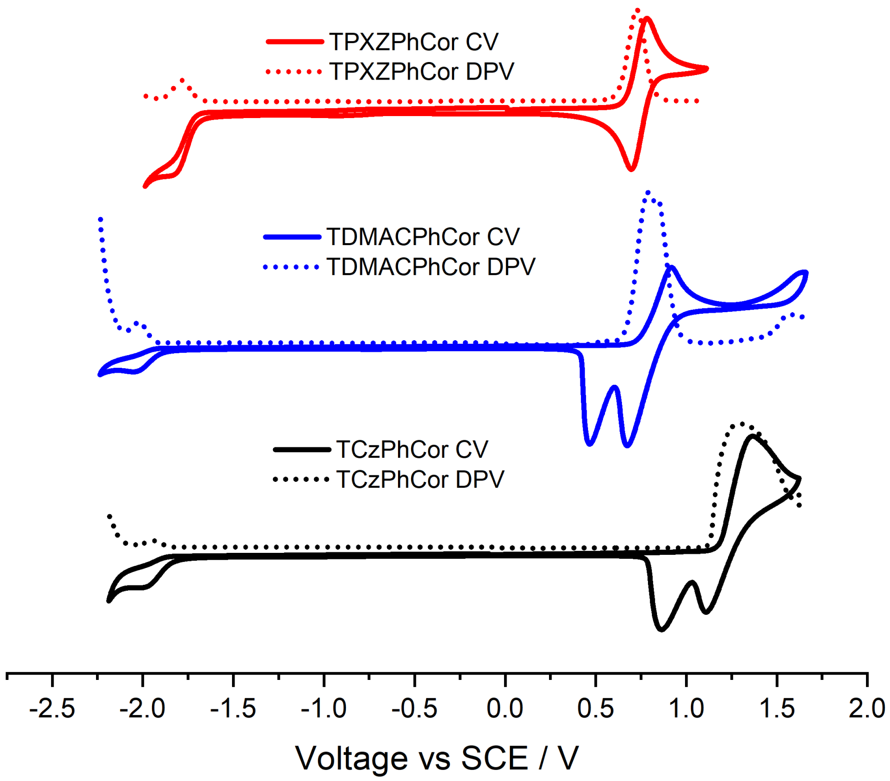

Figure **S41**. Cyclic and differential pulse voltammograms measured in degassed DCM with 0.1 M [*n*Bu_4N]PF_6 as the supporting electrolyte and Fc/Fc^+ as the internal reference (0.46 V vs SCE).[10] Scan rate = 100 mV s^{-1}.

Photophysical characterization

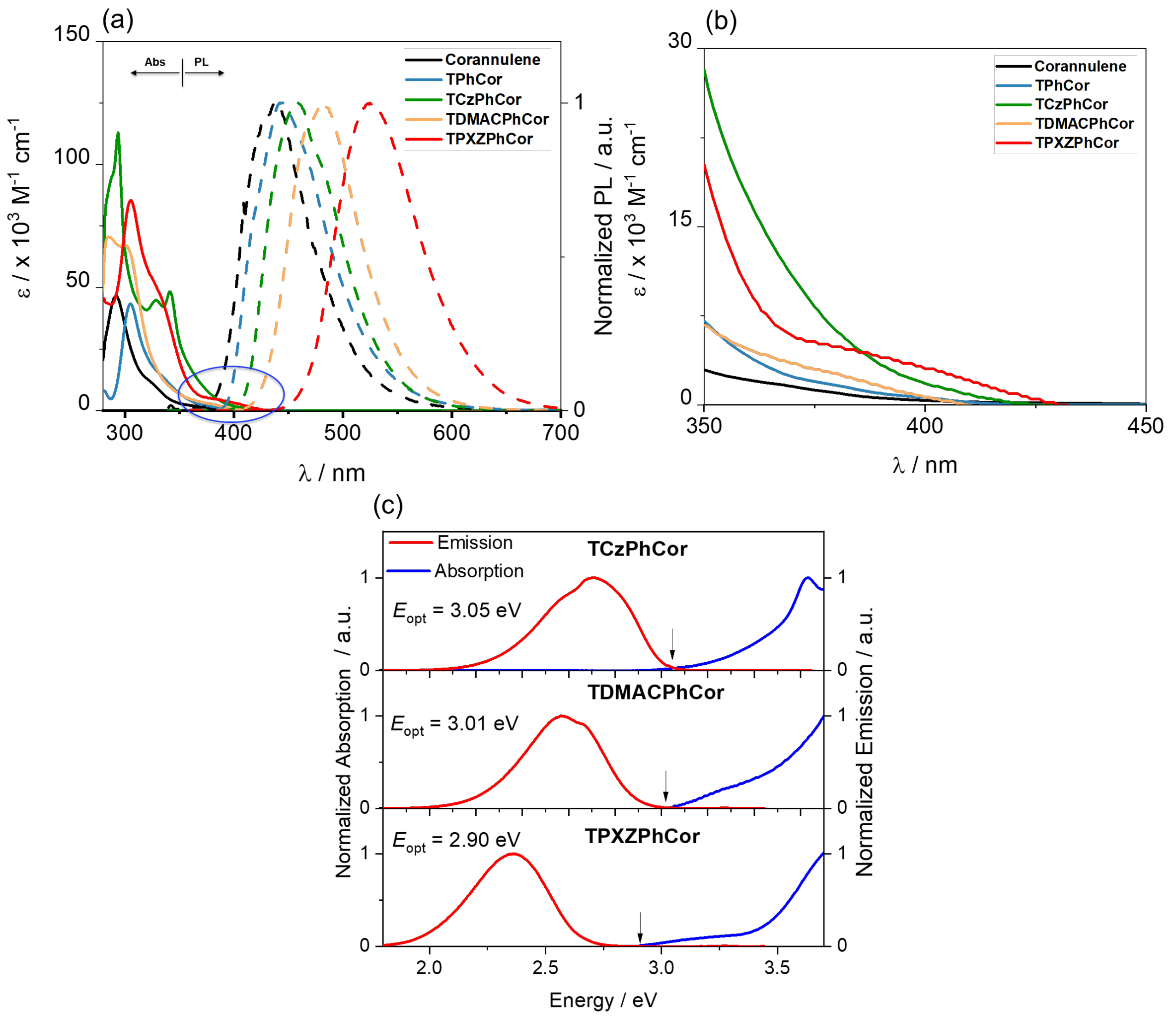

Figure **S42**. (a) UV-vis absorption and steady-state PL spectra for **Corannulene**, **TPhCor**, **TCzPhCor**, **TDMACPhCor** and **TPXZPhCor** in toluene (λ_{exc} = 340 nm); (b) Zoom of the absorption spectra of from 350 to 450 nm; (c) The optical bandgaps were determined from the intersection point of the normalized absorption and emission spectra for **TCzPhCor**, **TDMACPhCor** and **TPXZPhCor**.

Table **S2.** Summary of photophysical properties of corannulene-based compounds

	λ_{abs}/ (ε / ×10^3 M^{-1} cm^{-1})/nm^a	λ_{PL}^{a} / nm	Φ_{PL}^{b} /%	λ_{PL}^{c} /nm	Φ_{PL}^{c} /%	τ_p^{c} /ns	τ_{Ph}^{d} /ms	λ_{PL}^{e} /nm	Φ_{PL}^{e} /%	τ_p^{e} /ns	τ_{Ph}^{H}^{f} /ms	τ_{Ph}^{L}^{f} /ms	S_1/T_1^{g} /eV	ΔE_{ST} /eV
**TPhCor**	305(43), 336 (13)	442	11(10)	443	13.0 (12.0)	15.1	144.2	-	-	-	-	-	3.07/ 2.43	0.64
**TCzPhCor**	238 (211), 229(114), 327(53), 339(64)	458	37 (34)	450	33.9 (33.8)	9.8	573.0	462	36.2 (35.0)	6.5	-	293.7	3.00/ 2.40	0.60
**TDMACPhCor**	261(167), 283(163),303(160)	476	21 (18)	465	28.5 (28.2)	25.2	286.1	485	32.1 (29.4)	19.8	96.8	343.3	2.96/ 2.42	0.54
**TPXZPhCor**	242(133),260(48) 279(38), 303(48), 333(21)	530	19 (16)	500	34.5 (24.5)	30.8	34.6	529	42.5 (24.5)	30.7	82.6	-	2.77/ 2.43	0.34

^{a} In PhMe at 298 K (λ_{exc}=340 nm). ^{b} Quinine sulfate in H_2SO_4 (aq) was used as the reference (Φ_{PL} = 54.6%, λ_{exc}=340 nm).[14] ^{c} Spin-coated 1 wt% **TPhCor**, 1 wt% for **TCzPhCor**, 1 wt% for **TDMACPhCor** and 1 wt% for **TPXZPhCor** doped in PMMA films and values were determined using an integrating sphere (λ_{exc}=340 nm). Values quoted are under N_2. Values in parentheses are in air. ^{d} PL decay detected at an emission wavelength of 580 nm for **TPhCor**, **TCzPhCor**, 530 nm for **TDMACPhCor** and 550 nm for **TPXZPhCor**. ^{e} Spin-coated 1 wt% for **TCzPhCor**, 7 wt% for **TDMACPhCor** and 15 wt% for **TPXZPhCor** doped in mCP films and values were determined using an integrating sphere (λ_{exc}=340 nm). Values quoted are under N_2. Values in parentheses are in air. ^{f} PL decay detected emission wavelength is 585 nm for **TCzPhCor** and **TDMACPhCor** for RTP T_1^L, 490 nm for **TDMACPhCor** and 530 nm for **TPXZPhCor** for RTP (T_1^H). ^{g} S_1 was obtained from the onset of the prompt emission (time-gated window: 1–100 ns) measured in toluene at 77 K and T_1 was obtained from the onset of the phosphorescence spectrum (time-gated window: 1–10 ms) measured in toluene at 77 K (λ_{exc}=343 nm).

The Lippert-Mataga equation, which defines the interactions between the solvent and the dipole moment of the solute, may be used to understand the effect of the solvent environment on the optical property of the three compounds:

$$hc(v_a - v_f) = hc(v_a^0 - v_f^0) + \frac{2(\mu_e - \mu_g)^2}{a^3} f(\varepsilon, n)$$

where f is the orientational polarizability of solvents, $\nu_a^0 - \nu_f^0$ correspond to the Stokes shifts when f is zero, μ_e is the excited-state dipole moment, μ_g is the ground-state dipole moment; a is the solvent cavity radius, derived from the Avogadro number (N), molecular weight (M), and density (d = 1.0 g/cm^3); ε and n are the solvent dielectric and the solvent refractive index, respectively; $f(\varepsilon, n)$ and a can be calculated respectively as follows:

$$f(\varepsilon, n) = \frac{\varepsilon - 1}{2\varepsilon + 1} - \frac{n^2 - 1}{2n^2 + 1}, a = (3M/4N\pi d)^{1/3}$$

The detailed data are listed in Table **S3**.

The ground-state dipoles of the three compounds could be estimated using density functional theory (DFT) calculations at the MO62X/6-31G(d,p) level of theory. Values of 1.31 D, 1.24 D and 2.22 D, for **TCzPhCor**, **TDMACPhCor** and **TPXZPhCor**, were calculated, respectively.

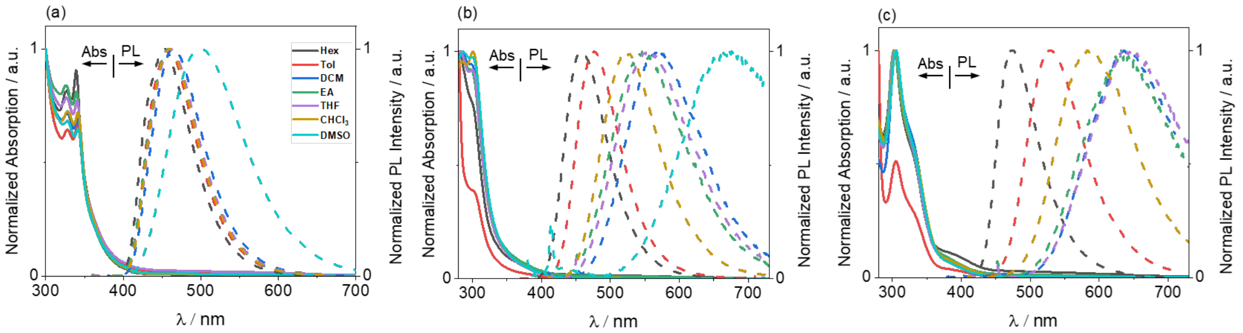

Figure **S43**. UV-vis absorption and solvatochromic PL study for (a) **TCzPhCor**, (b) **TDMACPhCor** and (c) **TPXZPhCor** (λ_{exc} = 340 nm).

Table **S3** Detailed absorption and emission peak positions of the three compounds in different solvents

Solvent	*ε*	*n*	*f* (*ε*, *n*)	**TCzPhCor**			**TDMACPhCor**			**TPXZPhCor**		
				λ_a / nm	λ_f / nm	ν_a-ν_f / cm^{-1}	λ_a / nm	λ_f / nm	ν_a-ν_f / cm^{-1}	λ_a / nm	λ_f / nm	ν_a-ν_f / cm^{-1}
Hexane	1.9	1.375	0.0012	340	454	7385	301	459	11436	303	475	11436
Toluene	2.38	1.494	0.014	340	458	7577	301	476	12214	304	530	12214
Butyl ether	3.08	1.399	0.096	340	456	7482	301	482	12475	304	532	12475
Isopropyl ether	3.88	1.368	0.145	340	455	7433	301	496	13061	304	551	13061
Chloroform	4.81	1.443	0.149	340	460	7672	301	524	14138	305	583	14138
Ethyl ether	4.34	1.352	0.167	340	455	7433	301	507	13498	305	570	13498
Ethyl acetate	6.02	1.372	0.200	340	460	7672	301	546	14907	305	632	14907
Tetrahydrofuran	7.58	1.407	0.210	340	461	7720	301	558	15301	305	639	15301
Methylene chloride	8.93	1.424	0.217	340	465	7906	301	570	15678	305	636	15678
Dimethyl formamide	37	1.427	0.276	340	499	9371	301	672	18341	305	-	-

λ_a: The first absorption band; λ_f Peak value of PL spectra obtained at 298 K, concentration 10^{-5} M, λ_{exc} = 340 nm.

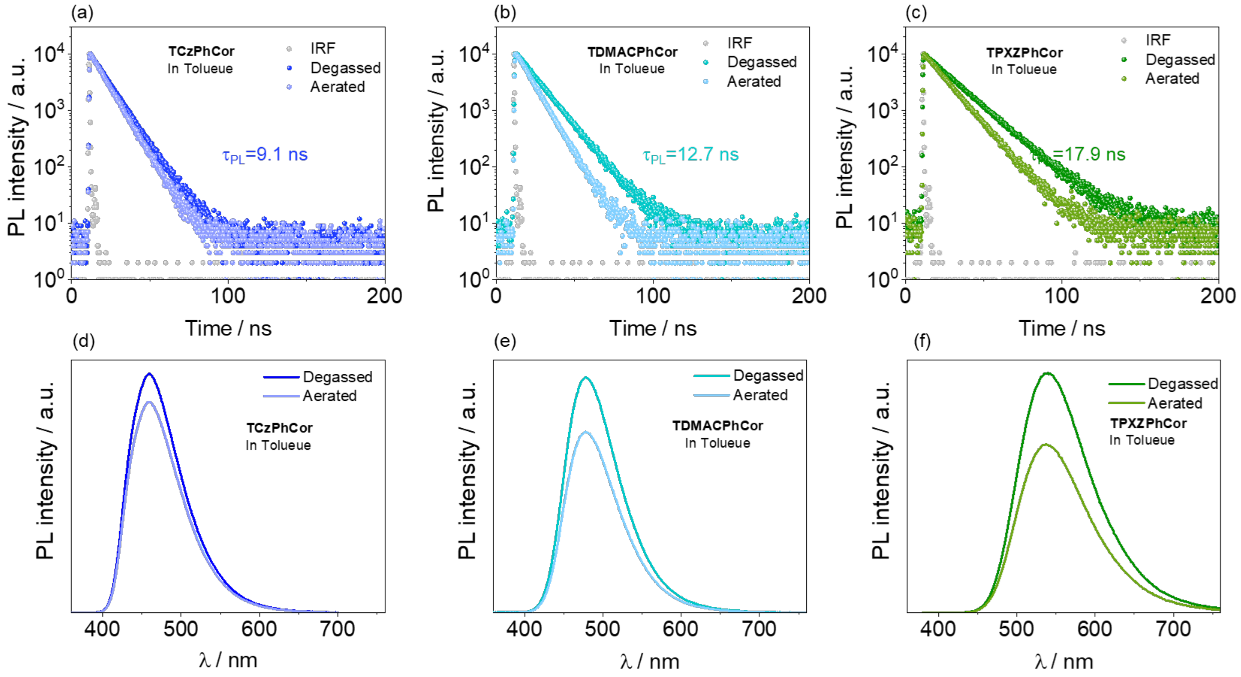

Figure **S44**. Time-resolved PL decay profiles of (a) **TCzPhCor**, (b) **TDMACPhCor** and (c) **TPXZPhCor** in aerated and degassed toluene (λ_{exc}= 379 nm). Steady-state PL spectra of (d) **TCzPhCor**, (e) **TDMACPhCor** and (f) **TPXZPhCor** in degassed and aerated toluene (λ_{exc}= 340 nm).

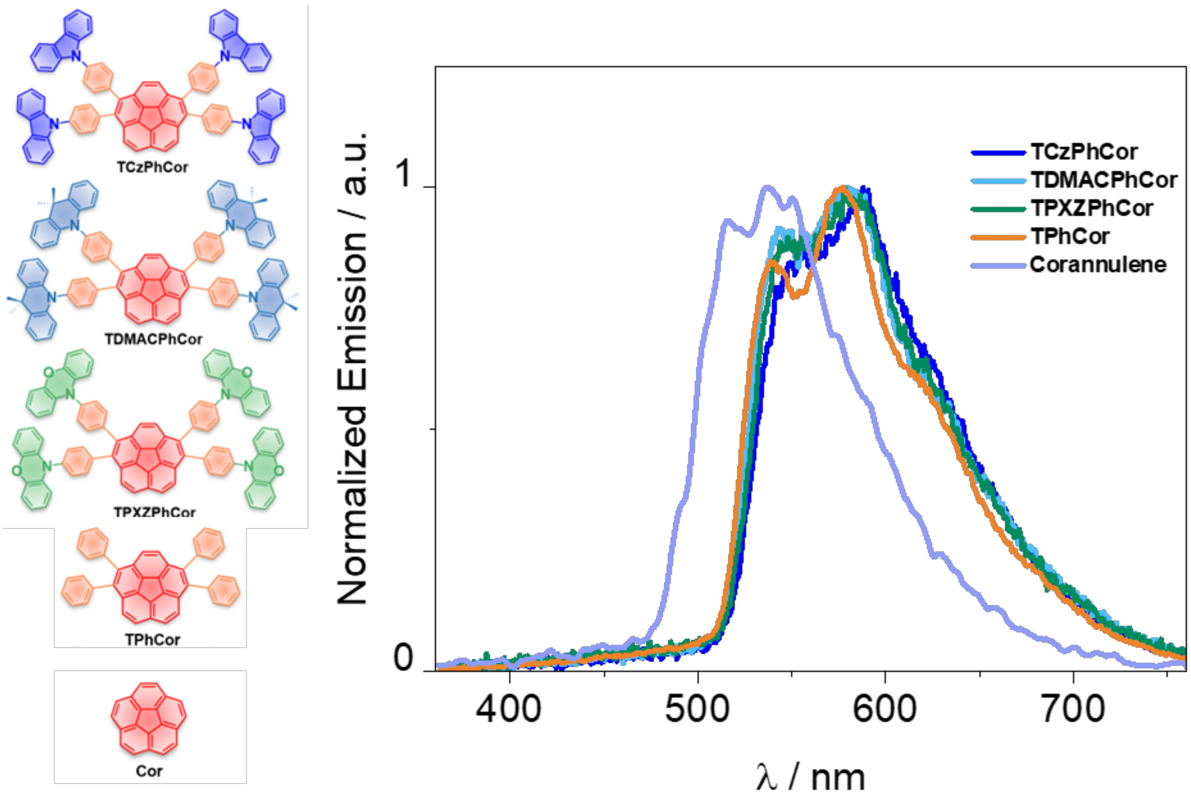

Figure **S45**. Phosphorescence spectra of **Corannulene**, **TPhCor, TCzPhCor**, **TDMACPhCor** and **TPXZPhCor** in toluene at 77 K (λ_{exc} = 343 nm, time-gated window: 1-10 ms).

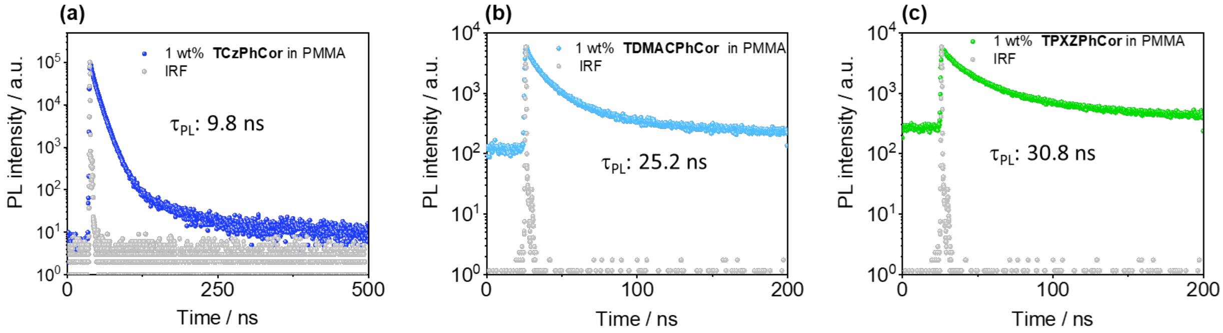

Figure **S46**. Time-resolved PL decay profiles of (a) 1wt% **TCzPhCor**, (b) 1 wt% **TDMACPhCor** and (c) 1wt% **TPXZPhCor** in PMMA under vacuum (λ_{exc} = 379 nm).

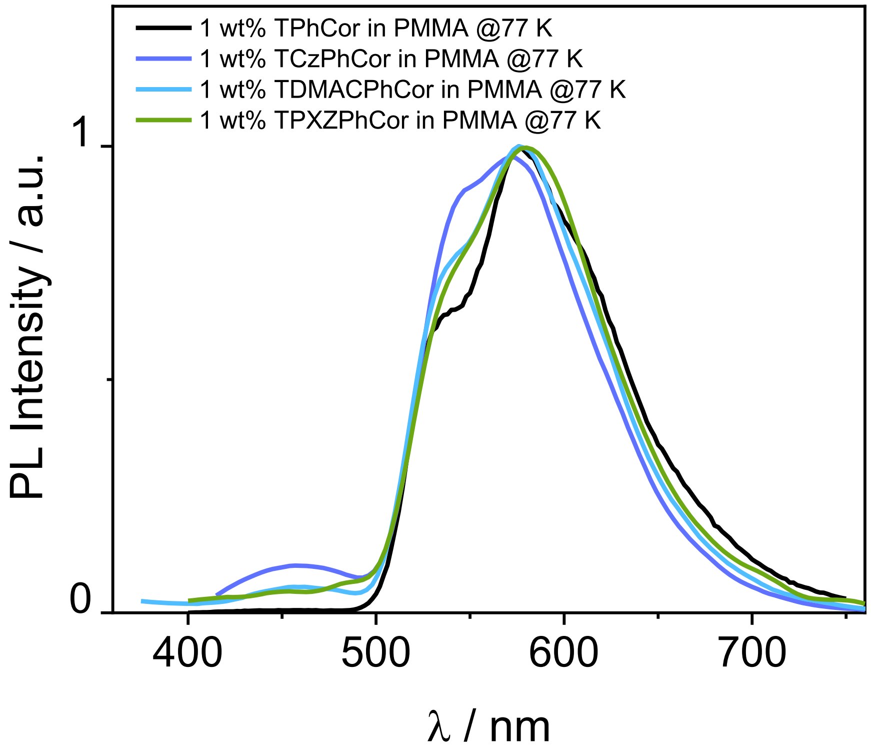

Figure **S47**. Normalized phosphorescence spectra of 1 wt% **TPhCor**, 1 wt% **TCzPhCor**, 1 wt% **TDMACPhCor** and 1 wt% **TPXZPhCor** doped in PMMA at 77 K in vacuum (λ_{exc}= 320 nm).

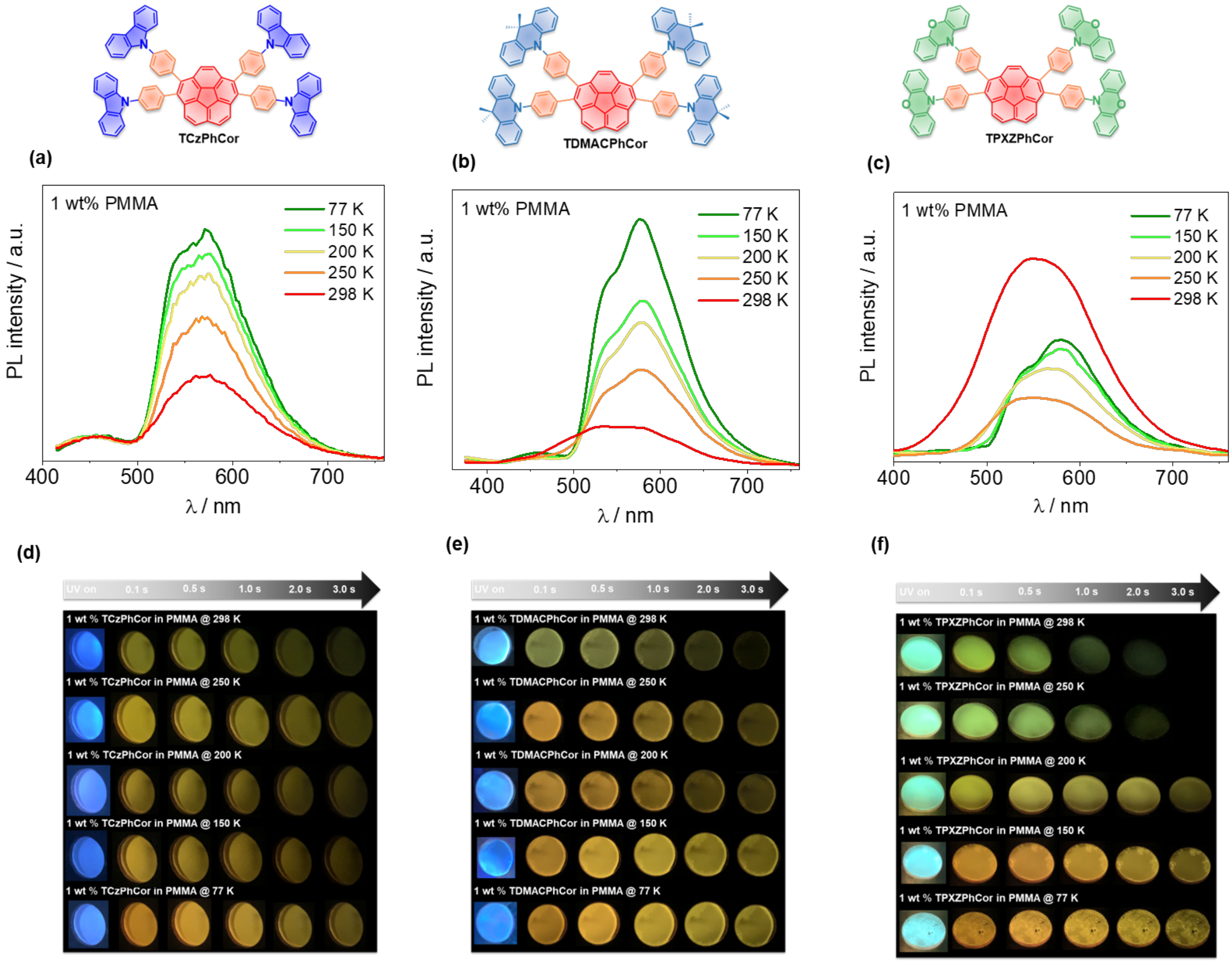

Figure **S48**. Temperature-dependent phosphorescence spectra of (a) 1 wt% **TCzPhCor**, (b) 1 wt% **TDMACPhCor** and (c) 1 wt% **TPXZPhCor** in PMMA in vacuum. (Time-gated window: 30–200 ms, λ_{exc}=320 nm). Images of (d) 1 wt% **TCzPhCor**, (e) 1 wt% **TDMACPhCor**, and (f) 1 wt% **TPXZPhCor** in PMMA showing afterglows at different temperatures (excitation source: 365 nm UV torch).

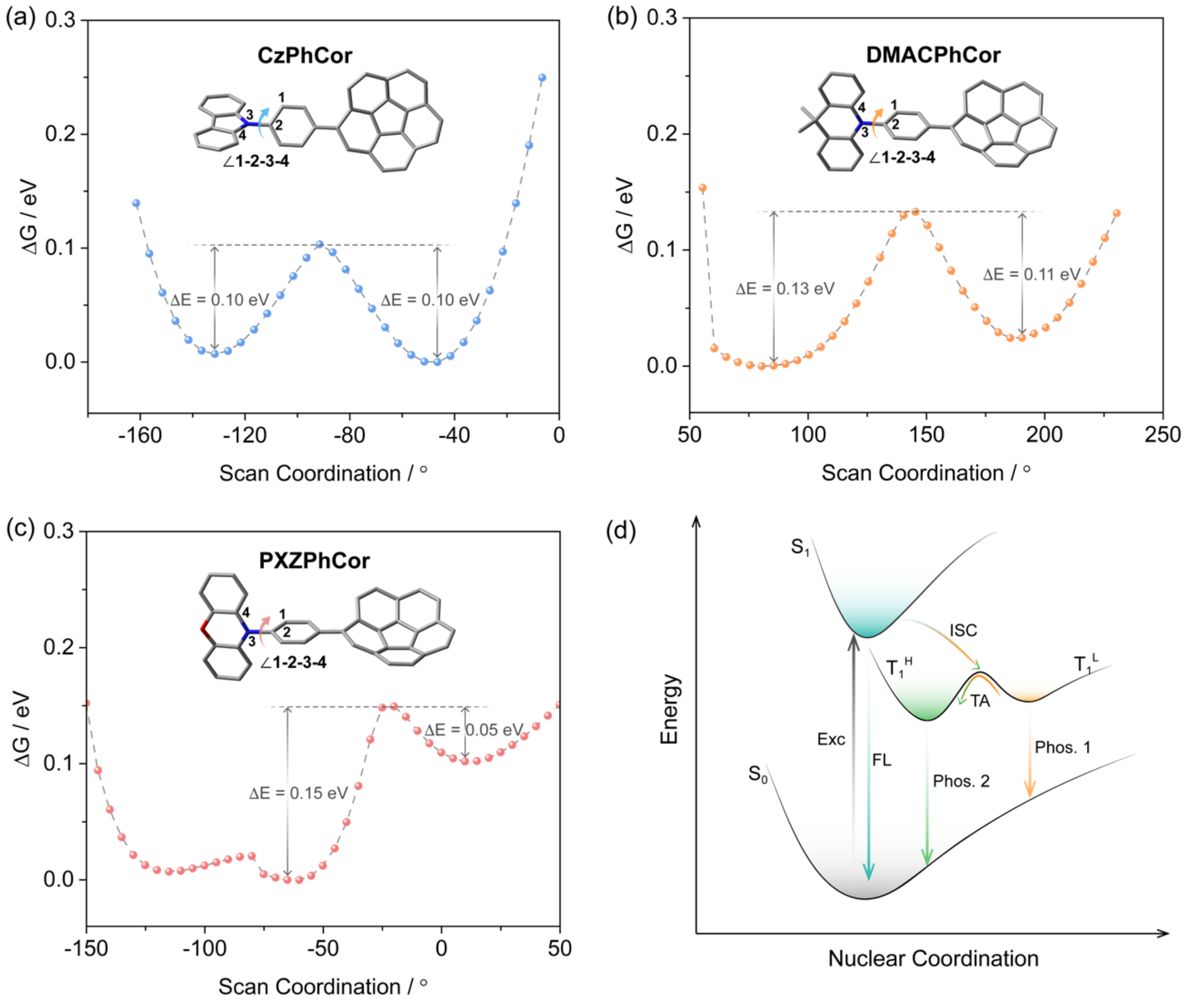

Figure **S49**. Potential energy scan of (a) CzPhCor, (b) DMACPhCor, and (c) PXZPhCor at the optimized T_1 geometry in the gas phase at uM06-2X/6-31G(d,p) level; inset: molecular structures associated with scanned torsion angles (∠1-2-3-4). (d) Mechanistic illustration of dual phosphorescence using a simplified Jablonski diagram; Exc: excitation; FL: fluorescence; TA: thermal activation; Phos.: phosphorescence. The PES modelling was carried out by progressively modulating the torsion angle between the donor (Cz, DMAC and PXZ) and benzene bridge. For CzPhCor, two triplet conformers were acquired showing the almost identical relative Gibbs energy (ΔG) indicates the same triplet energy (Figure S49a), which could explain why dual phosphorescence is not present in TCzPhCor. However, two different triplet conformers co-exist in both DMACPhCor and PXZPhCor. These two conformers are separated by a transition state (TS) geometry. For DMACPhCor, the barrier energies between the TS geometry and the two conformers are 0.13 eV and 0.11 eV (Figure S49b), which indicate that there is a rapid interconversion between the two conformers at ambient temperatures. As Figures 6b and 7e indicate, there are two contributions to the phosphorescence emission of TDMACPhCor, which we have attributed to T_1^H and T_1^L states. For PXZPhCor, the

interconversion mainly occurs from T_1^L to T_1^H conformers at room temperature due to a much smaller energy barrier between T_1^L and TS conformers compared to that between T_1^H and TS conformers (0.05 eV vs 0.15 eV) (Figure S49c). This is consistent with the T_1^H-dominated RTP recorded in TPXZPhCor (Figures 6c and 7f). Assuming these model systems accurately reflect the conformational dynamics of the tetrasubstituted compounds in the study then a plausible Jablonski diagram can be formulated (Figure S49d, also presented in Figure 6d), where at room temperature T_1^H RTP can be thermally populated from the T_1^L state. At low temperatures, T_1^L phosphorescence dominates due to the inhibited thermal activation.

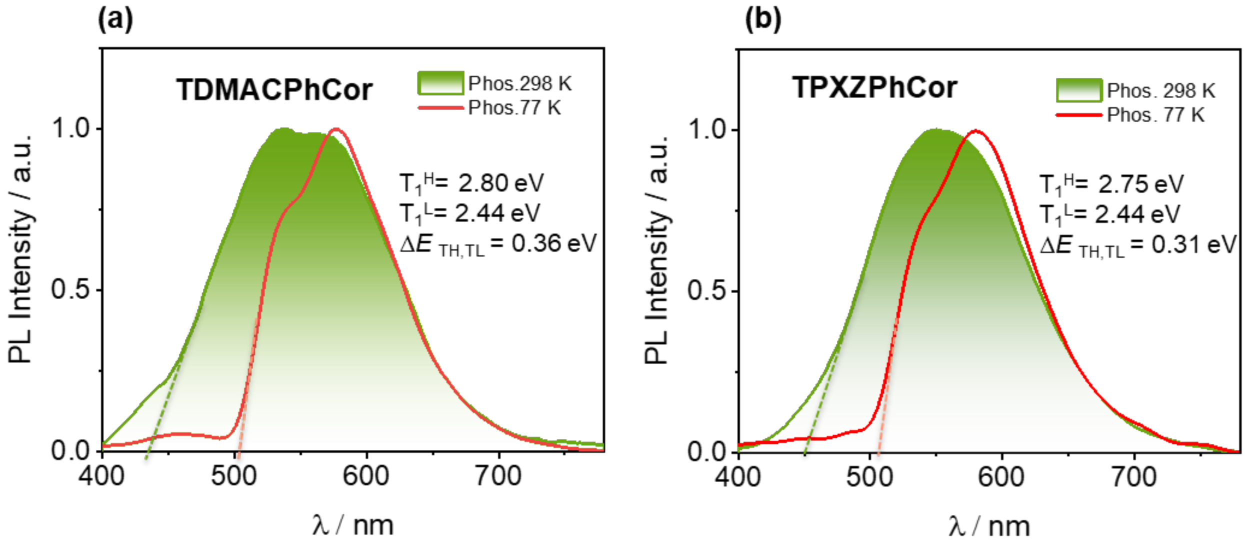

Figure **S50**. Phosphorescence of (a) 1 wt% **TDMACPhCor** and (b) 1 wt% **TPXZPhCor** in PMMA at 298 K and 77 K (time-gated window: 30-200 ms, λ_{exc}=320 nm).

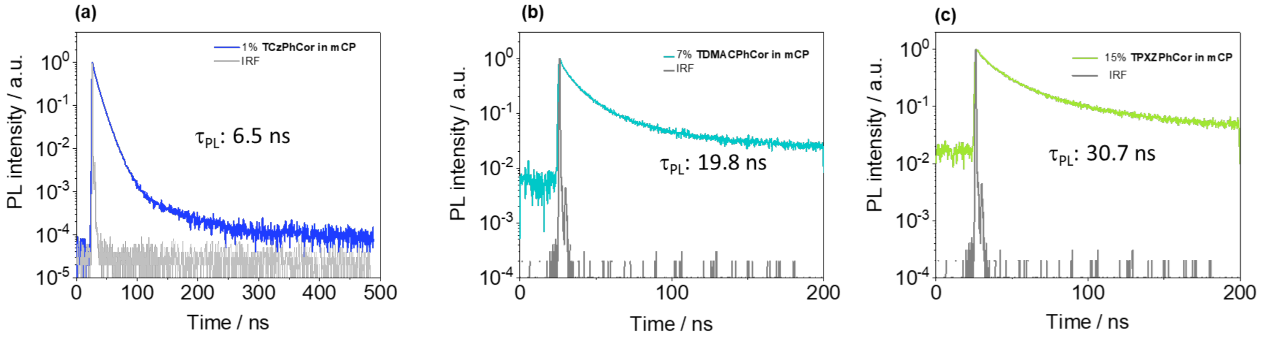

Figure **S49**. Time-resolved PL decay profiles of (a) 1 wt% **TCzPhCor**, (b) 7 wt% **TDMACPhCor** and (c)15 wt% **TPXZPhCor** in mCP in vacuum at 298 K (λ_{exc}= 379 nm).

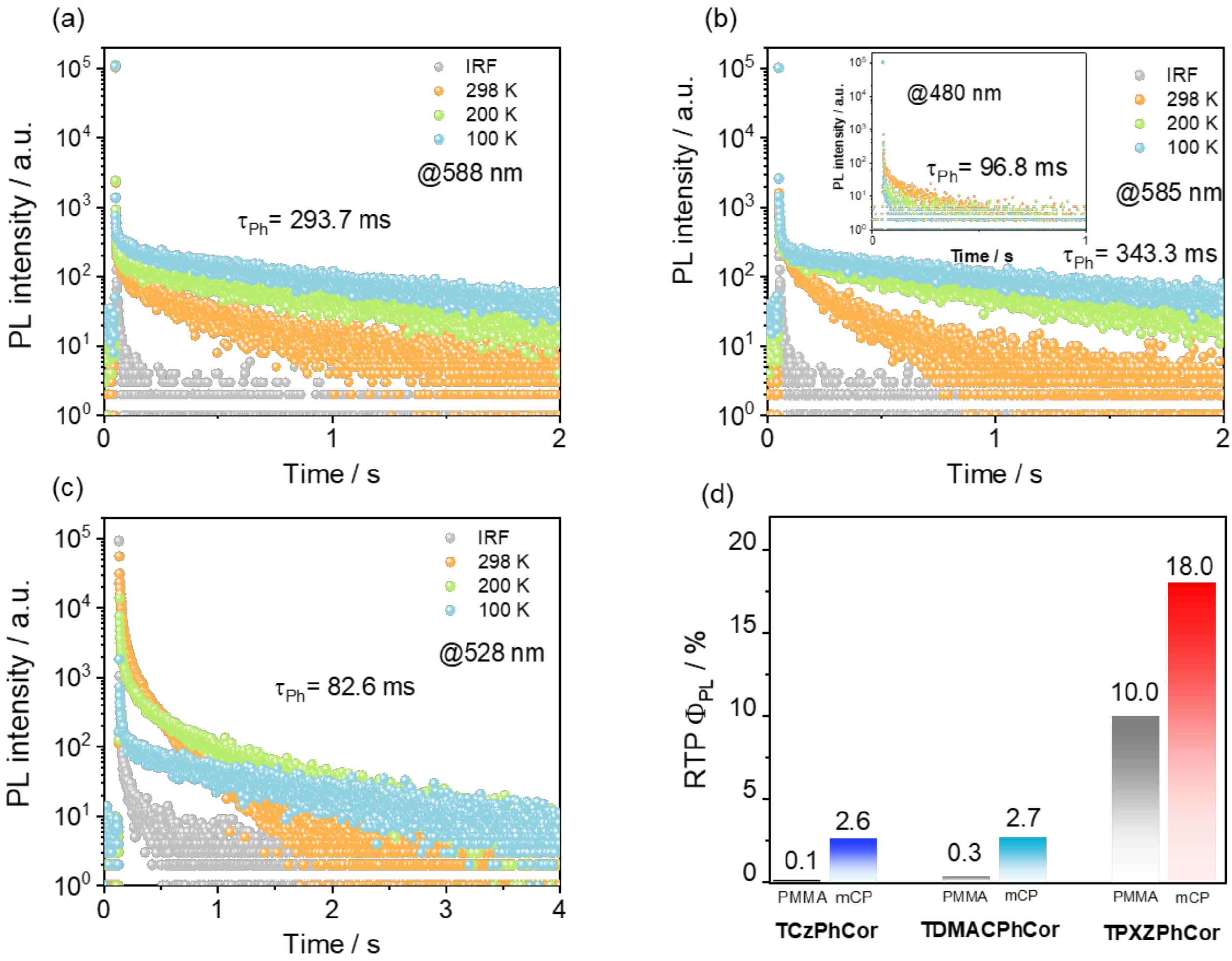

Figure **S50**. Temperature-dependent time-resolved PL decay of (a) 1 wt% **TCzPhCor**, (b) 7 wt% **TDMACPhCor** and (c) 15 wt% **TPXZPhCor** in mCP in vacuum (λ_{exc}= 379 nm); (d) RTP Φ_{PL} values of 1 wt% **TCzPhCor**, 7 wt% **TDMACPhCor** and 15 wt% **TPXZPhCor** in PMMA and mCP.

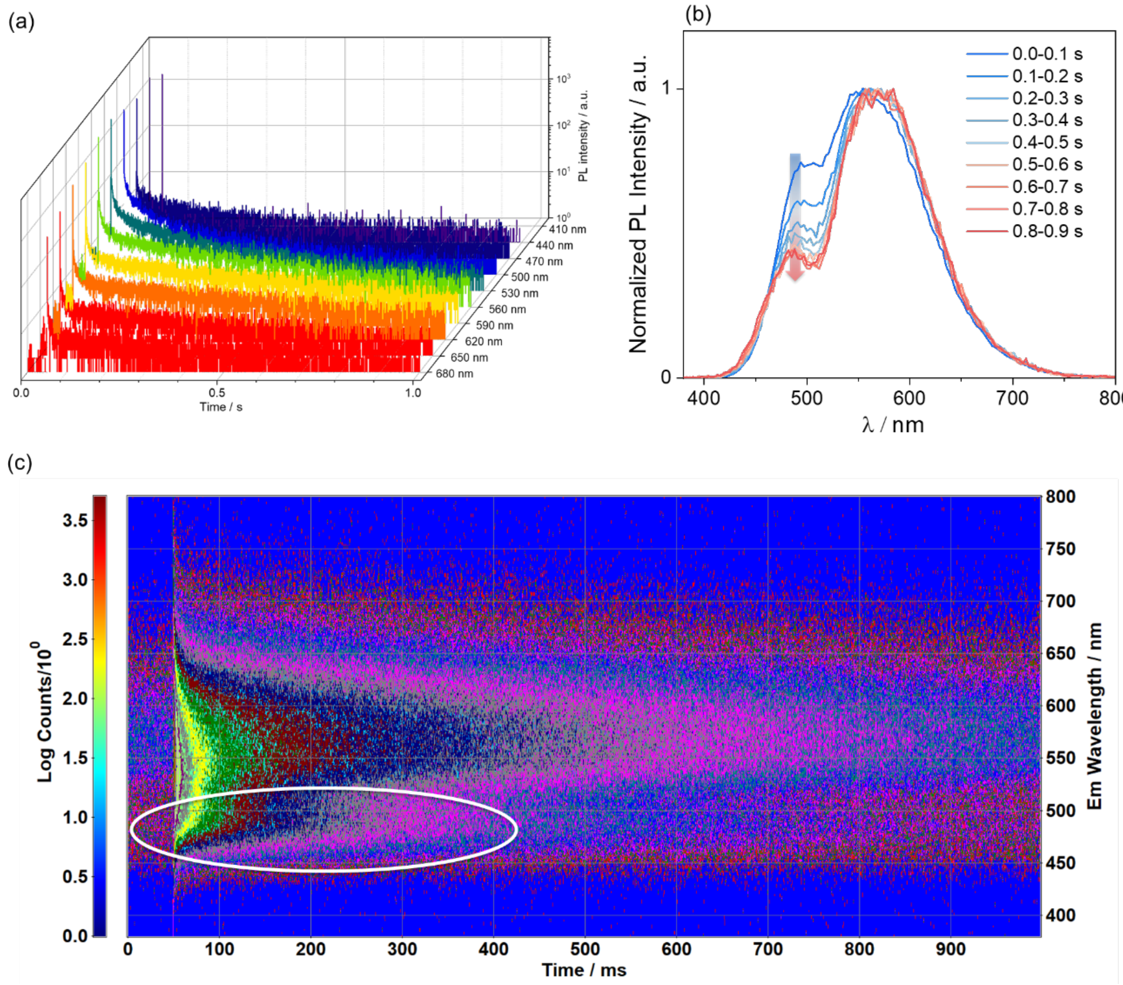

Figure **S51.** (a) Time-resolved PL decay at different emission wavelength of 7wt% TDMACPhCor in mCP (λ_{exc}=320 nm); (b) Phosphorescence of 7 wt% TDMACPhCor in mCP recorded at various time-gated windows at room temperature (λ_{exc}=320 nm); (c) 2D time-resolved PL decay vs emission wavelength mapping (λ_{exc}=320 nm).

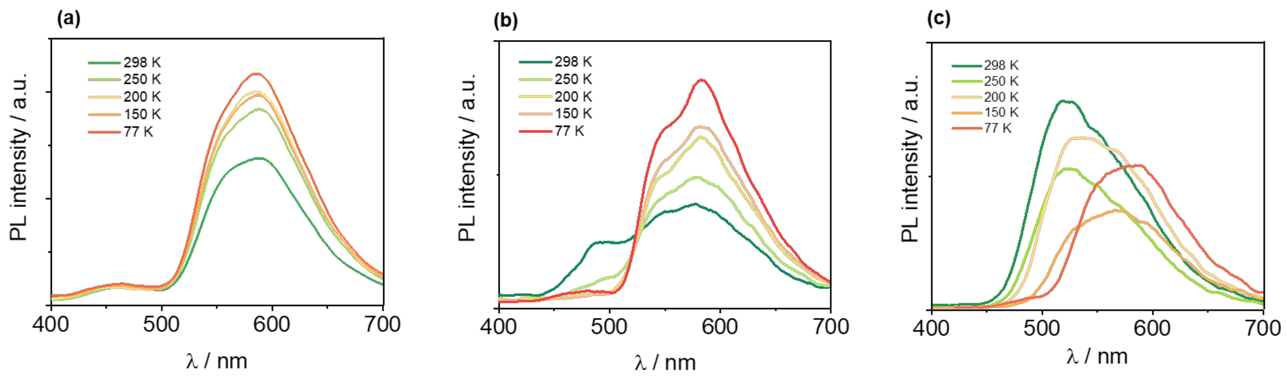

Figure **S52**. Temperature-dependent phosphorescence spectra of (a) 1 wt% **TCzPhCor**, (b) 7 wt% **TDMACPhCor** and (c) 15 wt% **TPXZPhCor** in mCP in vacuum. (Time-gated window: 30-200 ms, λ_{exc}= 320 nm).

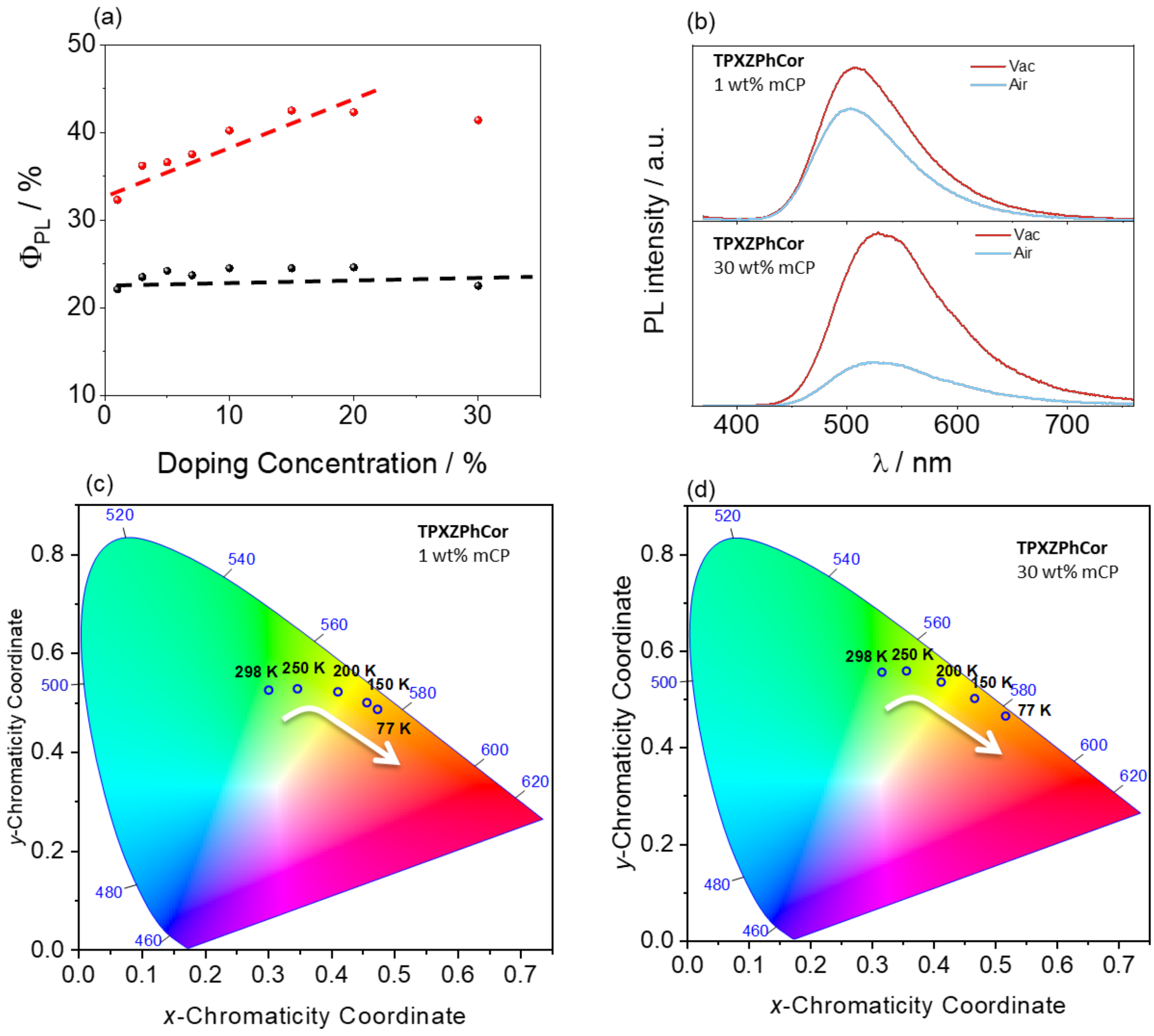

Figure **S53**. (a) Relationship between Φ_{PL} and doping concentration of **TPXZPhCor** in mCP; black dot and red dot represent F_{PL} recorded in air and N_2, respectively. (b) Steady-state PL spectra of 1 wt% and 30 wt% **TPXZPhCor** in mCP at 298 K in vacuum and air, λ_{exc}= 320 nm. Time-dependent changes in CIE coordinates of phosphorescence of (c) 1 wt% **TPXZPhCor** and (d) 30 wt% **TPXZPhCor** in mCP.

References

(1) Reddy, S. S.; Sree, V. G.; Gunasekar, K.; Cho, W.; Gal, Y.-S.; Song, M.; Kang, J.-W.; Jin, S.-H. Highly Efficient Bipolar Deep-Blue Fluorescent Emitters for Solution-Processed Non-Doped Organic Light-Emitting Diodes Based on 9,9-Dimethyl-9,10-Dihydroacridine/Phenanthroimadazole Derivatives. *Adv. Opt. Mater.* **2016**, *4* (8), 1236–1246. https://doi.org/10.1002/adom.201600217.

(2) Butterfield, A. M.; Gilomen, B.; Siegel, J. S. Kilogram-Scale Production of Corannulene. *Org. Process Res. Dev.* **2012**, *16*, 664–676. https://doi.org/10.1021/op200387s.

(3) Zhao, Y.; Truhlar, D. G. The M06 Suite of Density Functionals for Main Group Thermochemistry, Thermochemical Kinetics, Noncovalent Interactions, Excited States, and Transition Elements: Two New Functionals and Systematic Testing of Four M06-Class Functionals and 12 Other Function. *Theor. Chem. Acc.* **2008**, *120* (1–3), 215–241. https://doi.org/10.1007/s00214-007-0310-x.

(4) Grimme, S. Density Functional Calculations with Configuration Interaction for the Excited States of Molecules. *Chem. Phys. Lett.* **1996**, *259* (1–2), 128–137. https://doi.org/10.1016/0009-2614(96)00722-1.

(5) Hirata, S.; Head-Gordon, M. Time-Dependent Density Functional Theory within the Tamm-Dancoff Approximation. *Chem. Phys. Lett.* **1999**, *314* (3–4), 291–299. https://doi.org/10.1016/S0009-2614(99)01149-5.

(6) Gao, X.; Bai, S.; Fazzi, D.; Niehaus, T.; Barbatti, M.; Thiel, W. Evaluation of Spin-Orbit Couplings with Linear-Response Time-Dependent Density Functional Methods. *J. Chem. Theory Comput.* **2017**, *13* (2), 515–524. https://doi.org/10.1021/acs.jctc.6b00915.

(7) Johnson, E. R.; Keinan, S.; Mori-Sánchez, P.; Contreras-García, J.; Cohen, A. J.; Yang, W. Revealing Noncovalent Interactions. *J. Am. Chem. Soc.* **2010**, *132* (18), 6498–6506. https://doi.org/10.1021/ja100936w.

(8) Lu, T.; Chen, F. Multiwfn: A Multifunctional Wavefunction Analyzer. *J. Comput. Chem.* **2012**, *33* (5), 580–592. https://doi.org/10.1002/jcc.22885.

(9) Humphrey, W.; Dalke, A.; Schulten, K. VMD: Visual Molecular Dynamics. *J. Mol. Graph.* **1996**, *14* (1), 33–38. https://doi.org/10.1016/0263-7855(96)00018-5.

(10) Connelly, N. G.; Geiger, W. E. Chemical Redox Agents for Organometallic Chemistry. *Chem. Rev.* **1996**, *96* (2), 877–910. https://doi.org/10.1021/cr940053x.

(11) Pommerehne, J.; Vestweber, H.; Guss, W.; Mahrt, R. F.; Bässler, H.; Porsch, M.; Daub, J. Efficient Two Layer Leds on a Polymer Blend Basis. *Adv. Mater.* **1995**, *7* (6), 551–554. https://doi.org/10.1002/adma.19950070608.

(12) Demas, J. N.; Crosby, G. A. The Measurement of Photoluminescence Quantum Yields.1 A Review2. *J. Phys. Chem.* **1971**, *75* (8), 991–1024.

(13) Fery-Forgues, S.; Lavabre, D. Are Fluorescence Quantum Yields so Tricky to Measure? A Demonstration Using Familiar Stationery Products. *J. Chem. Educ.* **1999**, *76* (9), 1260–1264. https://doi.org/10.1021/ed076p1260.

(14) Melhuish, W. H. Quantum Efficiencies of Fluorescence of Organic Substances. *J. Phys. Chem.* **1961**, *65* (65), 229–235.

(15) *CrystalClear-SM Expert* v2.1. Rigaku Americas, *The Woodlands, Texas, USA,* and Rigaku Corporation, *Tokyo*, *Japan*, **2015**.

(16) *CrysAlisPro* v1.171.38.46, v1.171.41.93a. Rigaku Oxford Diffraction, *Rigaku Corporation, Oxford, U.K.* **2015-2020**.

(17) Sheldrick, G. M. SHELXT–Integrated Space-Group and Crystal-Structure Determination. *Acta Crystallogr. Sect. A Found. Adv.* **2015**, *71* (1), 3–8.

(18) Burla, M. C.; Caliandro, R.; Camalli, M.; Carrozzini, B.; Cascarano, G. L.; Giacovazzo, C.; Mallamo, M.; Mazzone, A.; Polidori, G.; Spagna, R. SIR2011: A New Package for Crystal Structure Determination and Refinement. *J. Appl. Crystallogr.* **2012**, *45* (2), 357–361. https://doi.org/10.1107/S0021889812001124.

(19) Sheldrick, G. M. Crystal Structure Refinement with SHELXL. *Acta Crystallogr. Sect. C Struct. Chem.* **2015**, *71* (Md), 3–8. https://doi.org/10.1107/S2053229614024218.

(20) Spek, A. L. PLATON SQUEEZE: A Tool for the Calculation of the Disordered Solvent Contribution to the Calculated Structure Factors. *Acta Crystallogr. Sect. C Struct. Chem.* **2015**, *71*, 9–18. https://doi.org/10.1107/S2053229614024929.

(21) Spek, A. L. Structure Validation in Chemical Crystallography. *Acta Crystallogr. Sect. D Biol.*

Crystallogr. **2009**, *65* (2), 148–155. https://doi.org/10.1107/S090744490804362X.

(22) Dolomanov, O. V; Bourhis, L. J.; Gildea, R. J.; Howard, J. A. K.; Puschmann, H. OLEX2: A Complete Structure Solution, Refinement and Analysis Program. *J. Appl. Crystallogr.* **2009**, *42* (2), 339–341.

(23) *CrystalStructure* v4.3.0. Rigaku Americas, *The Woodlands, Texas, USA*, and Rigaku Corporation, *Tokyo*, *Japan*, **2018**.

(24) Lv, H.; Ma, R.; Zhang, X.; Li, M.; Wang, Y.; Wang, S.; Xing, G. Surfactant-Modulated Discriminative Sensing of HNO and H2S with a Cu2+-Complex-Based Fluorescent Probe. *Tetrahedron* **2016**, *72* (35), 5495–5501. https://doi.org/10.1016/j.tet.2016.07.039.

(25) Venkatramaiah, N.; Dinesh Kumar, G.; Chandrasekaran, Y.; Ganduri, R.; Patil, S. Efficient Blue and Yellow Organic Light-Emitting Diodes Enabled by Aggregation-Induced Emission. *ACS Appl. Mater. Interfaces* **2018**, *10*, 3838–3847. https://doi.org/10.1021/acsami.7b11025.

(26) Park, I. S.; Lee, S. Y.; Adachi, C.; Yasuda, T. Full-Color Delayed Fluorescence Materials Based on Wedge-Shaped Phthalonitriles and Dicyanopyrazines: Systematic Design, Tunable Photophysical Properties, and OLED Performance. *Adv. Funct. Mater.* **2016**, *26* (11), 1813–1821. https://doi.org/10.1002/adfm.201505106.

(27) Liu, N.; Wang, B.; Chen, W.; Liu, C.; Wang, X.; Hu, Y. A General Route for Synthesis of N-Aryl Phenoxazines via Copper(i)-Catalyzed N-, N-, and O-Arylations of 2-Aminophenols. *RSC Adv.* **2014**, *4* (93), 51133–51139. https://doi.org/10.1039/c4ra09593f.
